# Supplementary material for: Profiling stress-triggered RNA condensation with photocatalytic proximity labeling
Source: Nat Commun. 2023 Nov 15;14:7390. doi: 10.1038/s41467-023-43194-2 (PMC10651888; doi:10.1038/s41467-023-43194-2)
Supplement: Supplementary file 1 — Supplementary information [file 41467_2023_43194_MOESM1_ESM.pdf]

Profiling stress-triggered RNA condensation with photocatalytic proximity labeling

Ziqi Ren[1,4], Wei Tang[2,4], Luxin Peng[1], Peng Zou[1,2,3,*]

1 College of Chemistry and Molecular Engineering, Synthetic and Functional Biomolecules Center, Beijing National Laboratory for Molecular Sciences, Key Laboratory of Bioorganic Chemistry and Molecular Engineering of Ministry of Education, PKU-IDG/McGovern Institute for Brain Research, Peking University, Beijing, 100871, China

2 Academy for Advanced Interdisciplinary Studies, Peking-Tsinghua Center for Life Sciences, Peking University, Beijing, 100871, China

3 Chinese Institute for Brain Research (CIBR), Beijing 102206, China

4 These authors contributed equally

* Correspondence: zoupeng@pku.edu.cn

Key Resources Table

Reagent or resource	Source	Identifier
**Antibodies**		
mouse anti-V5	Biodragon	B1005
rabbit anti-G3BP2	abcam	ab86135; RRID: AB_1925011
rabbit anti-TIA1	abcam	ab140595; RRID: AB_2687963
rabbit anti-G3BP	abcam	ab181150; RRID: AB_2847886
Goat anti-mouse-Alexa Fluor 488	ThermoFisher	A-11029; RRID: AB_2534088
Goat anti-rabbit-Alexa Fluor 568	ThermoFisher	A-11011; RRID: AB_143157
Goat anti-rabbit-Alexa Fluor 647	ThermoFisher	A-21244; RRID: AB_2535812
Streptavidin-Alexa Fluor 568	ThermoFisher	S21374
Streptavidin-Alexa Fluor 647	ThermoFisher	S11226
Rabbit Anti-Goat IgG H&L (HRP)	Biodragon	BF03008
rabbit anti-eIF2α	CST	5324
rabbit-anti-EIF2S1 (phospho S51)	abcam	ab32157; RRID: AB_732117
**Chemicals, peptides and recombinant proteins**		
Dulbecco's Modified Eagle's Medium (DMEM)	Gibco	C11995500BT
McCoy's 5A medium	BI	01-075-1ACS
0.25% Trypsin-EDTA	Life Technologies	25200056
Fetal bovine serum	Gibco	10099141
Lipofectamine 2000	Invitrogen	11668019

Opti-Minimal Essential Medium (Opti-MEM)	Gibco	31985062
Blasticidin	Selleck	S7419
TRIzol reagent	Invitrogen	15596018
Biotin-PEG$_3$-azide	Click Chemistry Tools	AZ104
DAPI	ThermoFisher	D1306
THPTA	Click Chemistry Tools	1010
BTTAA	Click Chemistry Tools	1236
Sodium ascorbate	Aladdin	S105024
Copper sulfate pentahydrate	Aladdin	C112401
Sodium arsenite	Sigma	S7400-100G
Propargylamine (PA)	Accela	SY002930
D-sorbitol	Sigma	S1876-100G
Urea	Sigma	U5378-100G
10% SDS solution	Life Technologies	AM9822
1 M Tris-HCl buffer, pH 7.5	Invitrogen	15567027
5 M NaCl, RNase free	Ambion	AM9759
Tween-20	Solarbio	T8200
Ultrapure water	Beyotime	ST872
Matrigel matrix	Corning	356234
PBS	Solarbio	P1020
10X PBS, RNase free	Life Technologies	AM9624
HBSS	Gibco	14025092

1N NaOH	Sigma	S2770
Formamide	Sigma	F9037
D-biotin	Invitrogen	B20656
0.5 M EDTA, pH 8.0	Life Technologies	AM9260G
Tris-EDTA buffer solution	Sigma	93283
DNase I	NEB	M0303
Triton X-100	Sigma	T8787
Yeast tRNA	Gibco	15401011
Glycogen, RNA grade	Fermentas	R0551
20×SSC buffer	Invitrogen	AM9770
Vanadyl ribonucleoside complex (VRC)	NEB	S1402S
1 M $MgCl_2$, RNase free	Invitrogen	AM9530G
tRNA from E. coli MRE 600	Roche	10109541001
UltraPure Salmon Sperm DNA Solution	Invitrogen	15632011
Dextran sulfate sodium salt	Sigma	D6001
Fluoromount-G Anti-Fade	SouthernBiotech	0100-35
Glycine	Solarbio	G8200
Paraformaldehyde	Sigma-Aldrich	V900894
Dynabeads MyOne Streptavidin C1	Life Technologies	65002
Chloroform	Tongguang	112049
Isopropanol	Tongguang	106030
VAHTS DNA Clean Beads	Vazyme	N411-02

Clarity Western ECL Substrate	Bio-Rad	1705061
RIPA lysis buffer	CWBIO	CW2334S
cOmplete™ (EDTA-free)	Roche	4693032001
Phosphatase Inhibitor Cocktail	bimake	B15001
**Critical commercial assays**		
RNA Clean and concentrator-100	Zymo Research	R1019
NEBNext Ultra II RNA Library Prep Kit for Illumina	NEB	E7770
Standard Sensitivity RNA Analysis Kit	AATI	DNF-471-0500
High Sensitivity NGS Fragment Analysis Kit	AATI	DNF-474-0500
**Experimental models: Cell lines**		
HEK293T	American Type Culture Collection, ATCC	
HEK293T/17	National Science & Technology Infrastructure--National BioMedical Cell-Line Resource, NSTI-BMCR	
U-2 OS	National Science & Technology Infrastructure--National BioMedical Cell-Line Resource, NSTI-BMCR	
**Software and algorithms**		
HISAT2 v2.1.0	Kim et al., 2015	RRID: SCR_015530
HTSeq v0.7.2	Anders et al., 2015	RRID: SCR_005514
cutadapt v1.18	Martin, 2011	RRID: SCR_011841
DESeq2 v1.34.0	Love et al., 2014	RRID: SCR_015687
pheatmap v1.0.12	pheatmap package in R	RRID: SCR_016418
OriginPro 2019	OriginLab	RRID: SCR_014212
R studio v4.1.2	Rstudio	https://www.rstudio.com/
MATLAB v9.10.0.1602886 (R2021a)	MathWorks	RRID: SCR_001622

**Oligonucleotides**	
smFISH oligos	Supplementary Data 4
G3BP1-miniSOG	BstbI-V5-AgeI-G3BP1-GS linker-BamH1-miniSOG-NheI
G3BP1-EGFP	BstbI-V5-AgeI-G3BP1-GS linker-BamH1-EGFP-NheI
miniSOG (untargeted)	BstbI-V5-AgeI-miniSOG-NES-NheI
**Others**	
smFISH analysis & SG co-localization script	https://github.com/PKUCHEMZouLab/CAP-seq_stress-granule

Supplemantary Figures

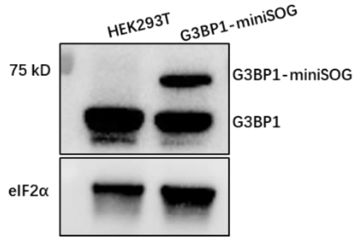

Supplementary Figure 1. Western blot analysis of G3BP1 in HEK293T cells stably expressing G3BP1-miniSOG. eIF2α was used as loading control.

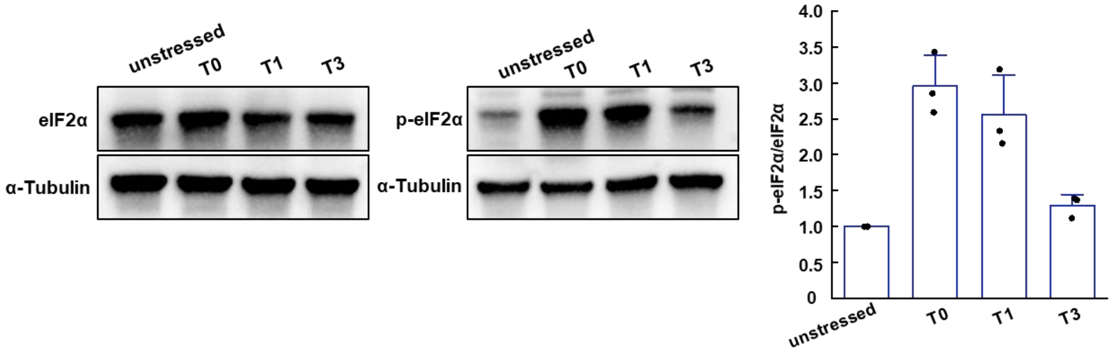

Supplementary Figure 2. Western blot analysis of eIF2α phosphorylation (p- eIF2α) in G3BP1-miniSOG HEK293T cells in unstressed (basal), stressed (T0), and recovery (T1 and T3) stages. The intensities of eIF2α phosphorylation are normalized with respect to eIF2α expression. Bars indicate mean values, lines indicate standard deviations (SD). The gel images presented here are representative examples from three independent experiments.

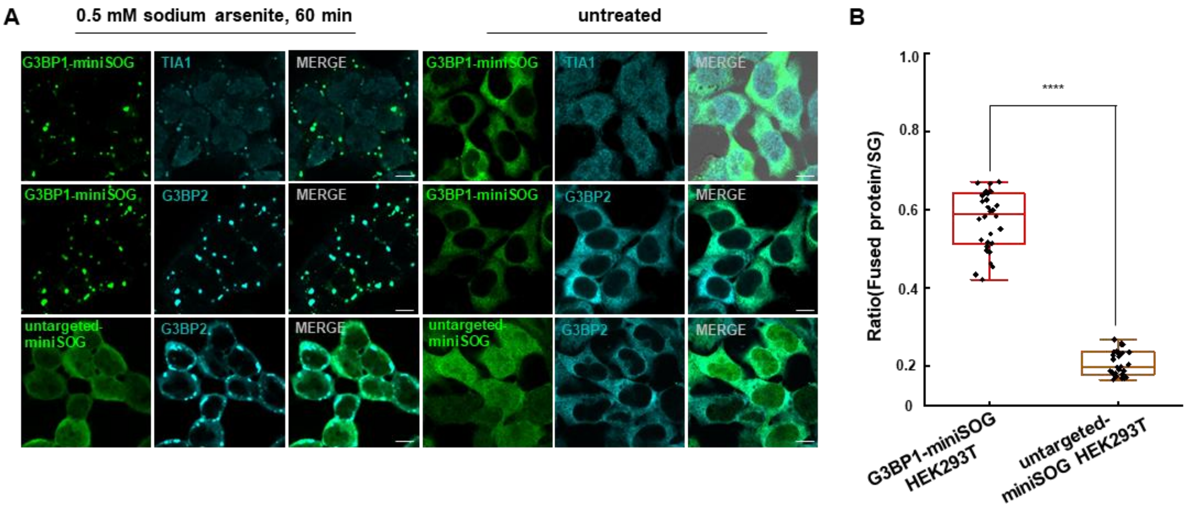

Supplementary Figure 3. Co-localization of G3BP1-miniSOG and untargeted-miniSOG with SG. **(A)** Immunofluorescence microscopy of HEK293T cells expressing G3BP1-miniSOG and untargeted miniSOG (green). Stressed cells were treated with 0.5 mM sodium arsenite for 60 min. Endogenous SG marker G3BP2 or TIA1 signal are shown in cyan. Fluorescence images are representative examples from three independent experiments. Scale bars, 10 μm. **(B)** Co-localization of G3BP1-miniSOG and untargeted-miniSOG with the SG marker G3BP2 (n = 30 from three independent experiments). The boxes mark the first and third quartiles; whiskers indicate the minima and maxima; the central lines represent the median. An unpaired Mann-Whitney U test (two-sided) was performed to evaluate the statistical significance. **** $p < 0.0001$.

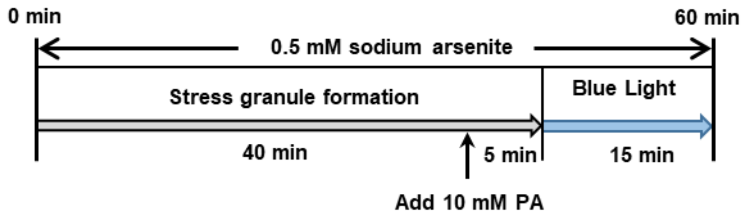

Supplementary Figure 4. Scheme of CAP-seq labeling under arsenite stress.

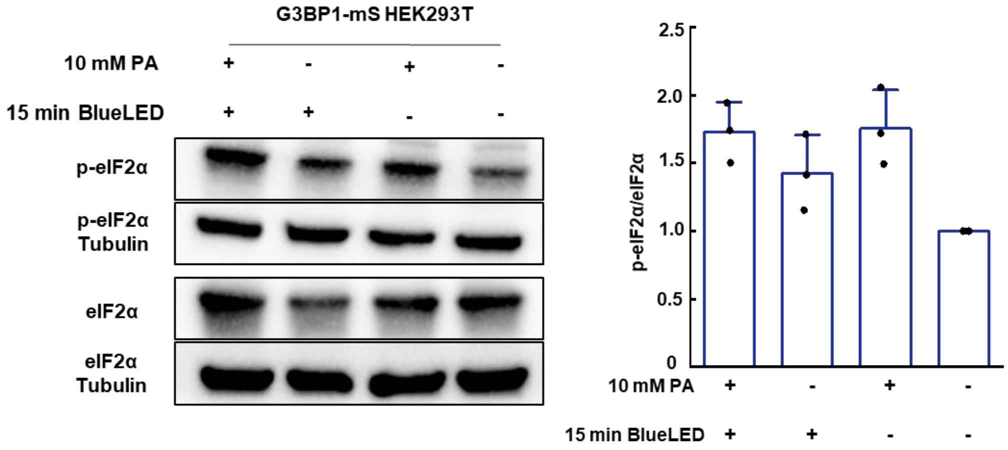

Supplementary Figure 5. Western blot analysis of eIF2α phosphorylation (p-eIF2α) in G3BP1-miniSOG HEK293T cells after CAP-seq labeling. The intensities of p-eIF2α are normalized with respect to eIF2α expression. The bars indicate mean values, lines indicate SD. The gel image presented here is a representative example from three independent experiments.

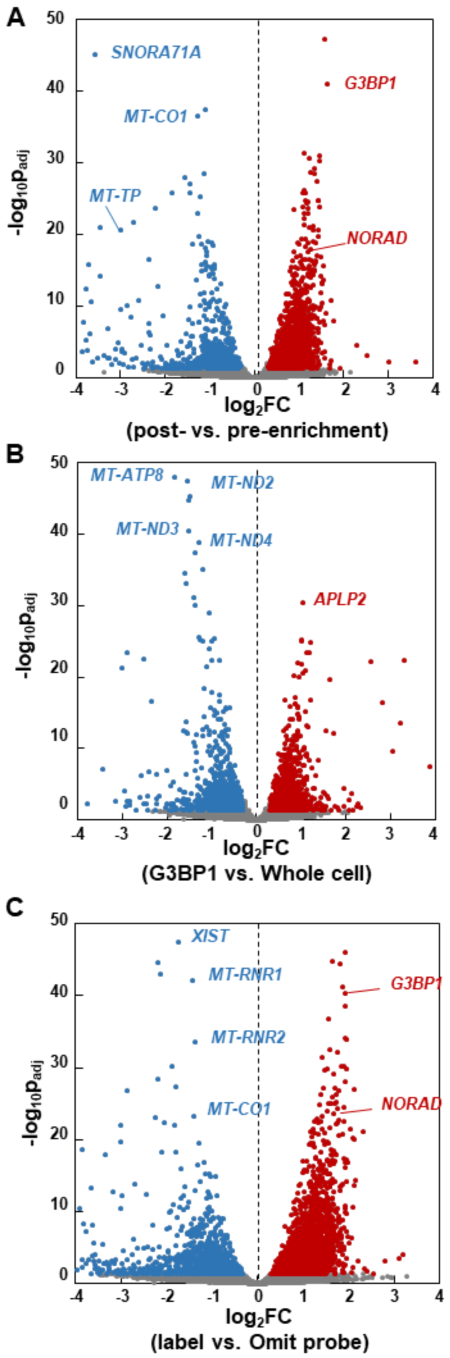

Supplementary Figure 6. Volcano plot showing enriched (red) and depleted (blue) RNA under arsenite-stressed HEK293T cells. **(A)** post- vs. pre-enrichment of G3BP1-miniSOG CAP-seq. **(B)** G3BP1-miniSOG vs. untargeted miniSOG. **(C)** G3BP1-miniSOG labeling vs. negative control omitting PA.

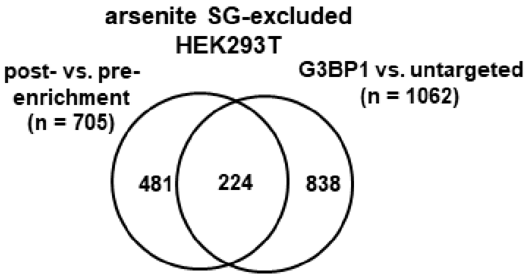

Supplementary Figure 7. Venn diagram comparing RNAs depleted in DESeq2 analysis between: 1) post- vs. pre-enrichment of G3BP1-miniSOG CAP-seq; 2) G3BP1-miniSOG vs. untargeted miniSOG. SG excluded RNAs in arsenite treated HEK293T are defined as the overlap between the two datasets.

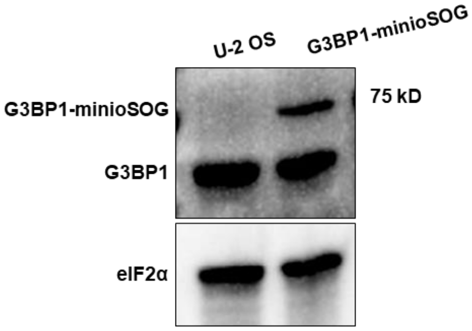

Supplementary Figure 8. Western blot analysis of G3BP1 in U-2 OS cells stably expressing G3BP1-miniSOG. eIF2α was used as loading control. The gel image presented here is a representative example from three independent experiments.

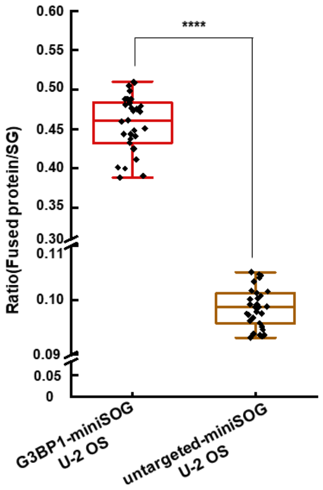

Supplementary Figure 9. Co-localization of G3BP1-miniSOG and untargeted-miniSOG with the SG marker G3BP2 (n = 30 from three independent experiments). The boxes mark the first and third quartiles; whiskers indicate the minima and maxima; the central lines represent the median. An unpaired Mann-Whitney U test (two-sided) was performed to evaluate the statistical significance. **** $p < 0.0001$.

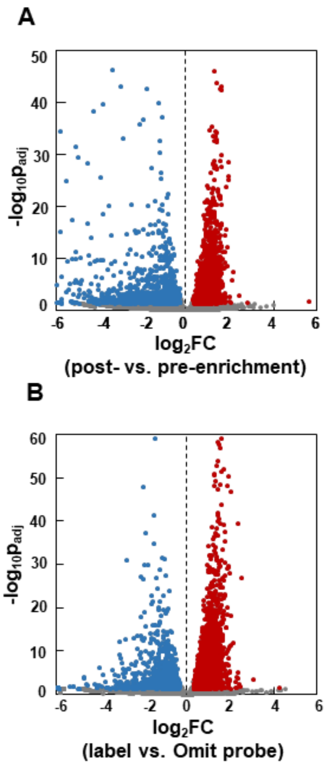

Supplementary Figure 10. Volcano plots showing enriched (red) and depleted (blue) RNA under arsenite-stressed U-2 OS cells. **(A)** post- vs. pre-enrichment of G3BP1-miniSOG CAP-seq. **(B)** G3BP1-miniSOG labeling vs. negative control omitting PA.

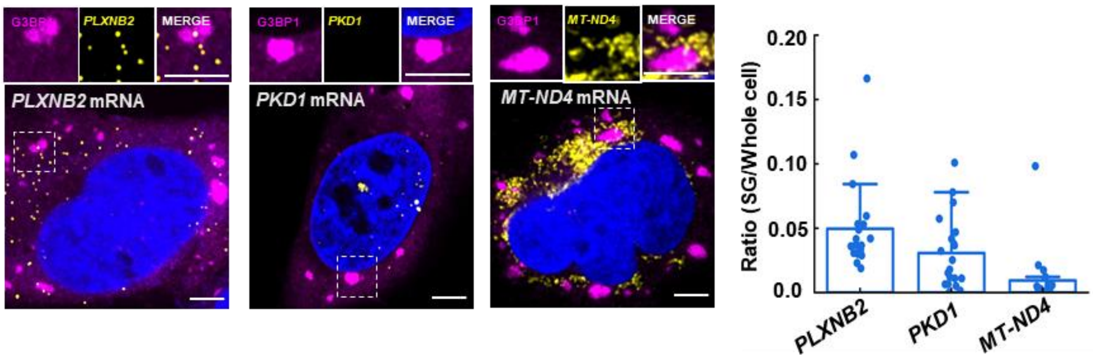

Supplementary Figure 11. smFISH validation of CAP-seq defined SG-excluded mRNAs (*PLXNB2*, *PKD1*, *MT-ND4*) in arsenite-stressed U-2 OS cells. Scale bars, 5 μm. Quantitation of the ratio of smFISH within SG vs. whole cell is shown on the right (n = 20 cells from three independent experiments). The bars represent the mean values, lines indicate SD.

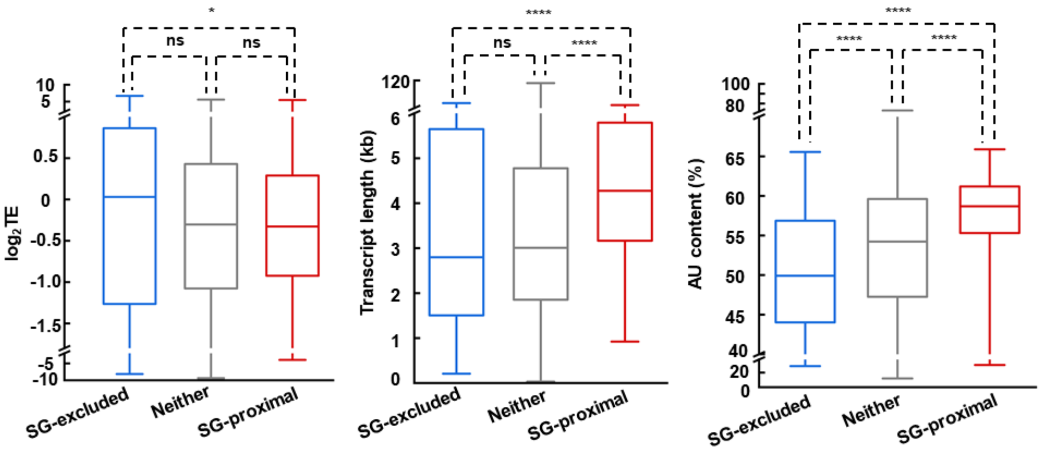

Supplementary Figure 12. Box plot comparing the translation efficiencies (left), transcript length (middle) and AU content (right) in SG-proximal and SG-excluded mRNAs in U-2 OS cells. The boxes mark the first and third quartiles; whiskers indicate the minima and maxima; the central lines represent the median. An unpaired Mann-Whitney U test (two-sided) was performed to evaluate the statistical significance. ns, not significant ($p > 0.05$); * $p < 0.05$; **** $p < 0.0001$. Translation efficiencies are provided by a previous study[1]. AU content and transcript length features are referenced from Ensembl website.

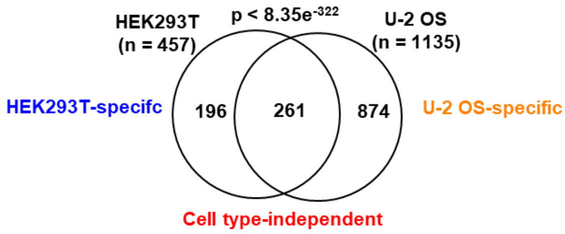

Supplementary Figure 13. Venn diagram comparing SG-proximal RNAs from arsenite-stressed HEK293T and U-2 OS cells. The p value was calculated by hypergeometric test.

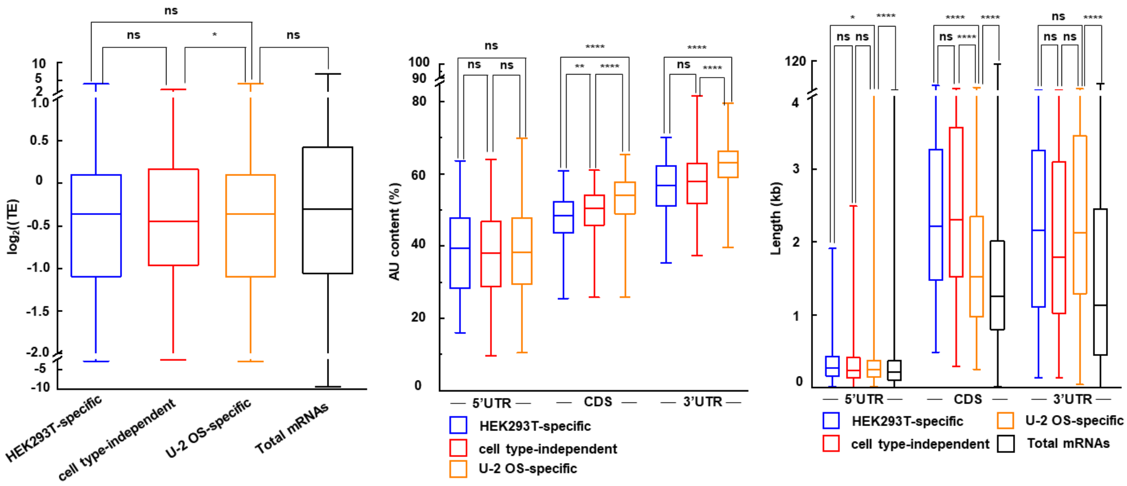

Supplementary Figure 14. Box plot comparing the translation efficiencies (left), AU content (middle) and transcript length (right) between HEK293T-specific, cell type-independent and U-2 OS-specific mRNAs. The boxes mark the first and third quartiles; whiskers indicate the minima and maxima; the central lines represent the median. An unpaired Mann-Whitney U test (two-sided) was performed to evaluate the statistical significance. ns, not significant ($p > 0.05$); * $p < 0.05$; ** $p < 0.01$; *** $p < 0.001$; **** $p < 0.0001$. Translation efficiencies are provided by a previous study[1]. AU content and transcript length features are referenced from Ensembl website.

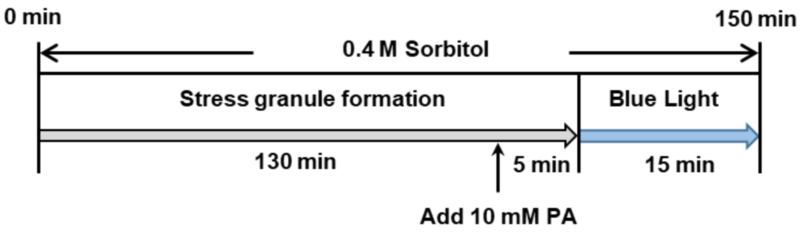

Supplementary Figure 15. Scheme of CAP-seq labeling under sorbitol stress.

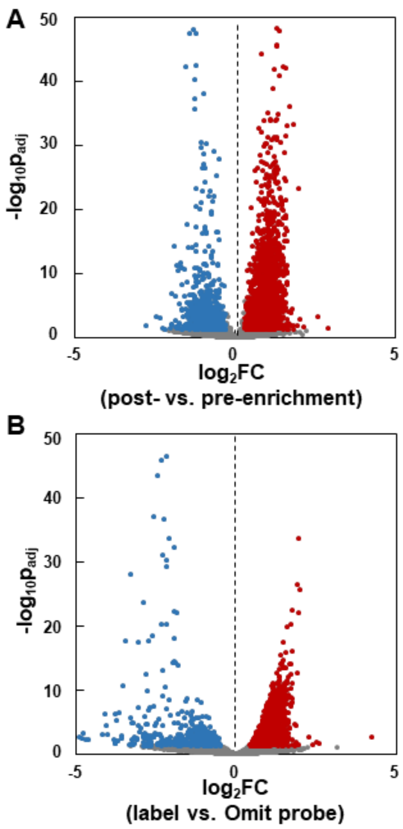

Supplementary Figure 16. Volcano plots of RNAs upregulated (red) and downregulated (blue) under sorbitol stressed HEK293T in DESeq2 analysis between **(A)** post- vs. pre-enrichment of G3BP1-miniSOG CAP-seq. **(B)** G3BP1-miniSOG labeling vs. negative control omitting PA.

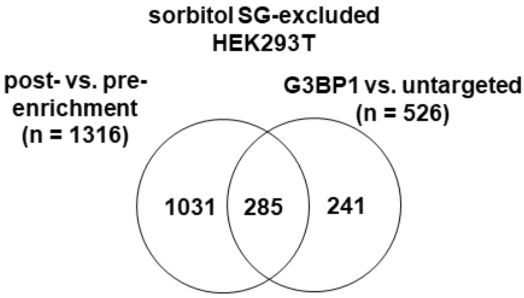

Supplementary Figure 17. Venn diagram comparing RNAs depleted in DESeq2 analysis between 1) post- vs. pre-enrichment of G3BP1-miniSOG CAP-seq; 2) G3BP1-miniSOG vs. untargeted miniSOG. SG-excluded RNAs in sorbitol treated HEK293T are defined as the overlap of the two datasets.

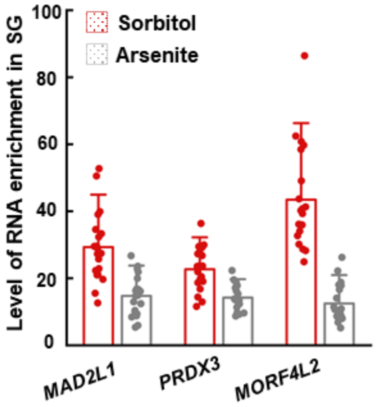

Supplementary Figure 18. Quantitation of the level of smFISH targets (*MAD2L1*, *PRDX3*, *MORF4L2*) enrichmnent in SGs (n = 20 cells from three independent experiments). The bars represent the mean values, lines indicate SD.

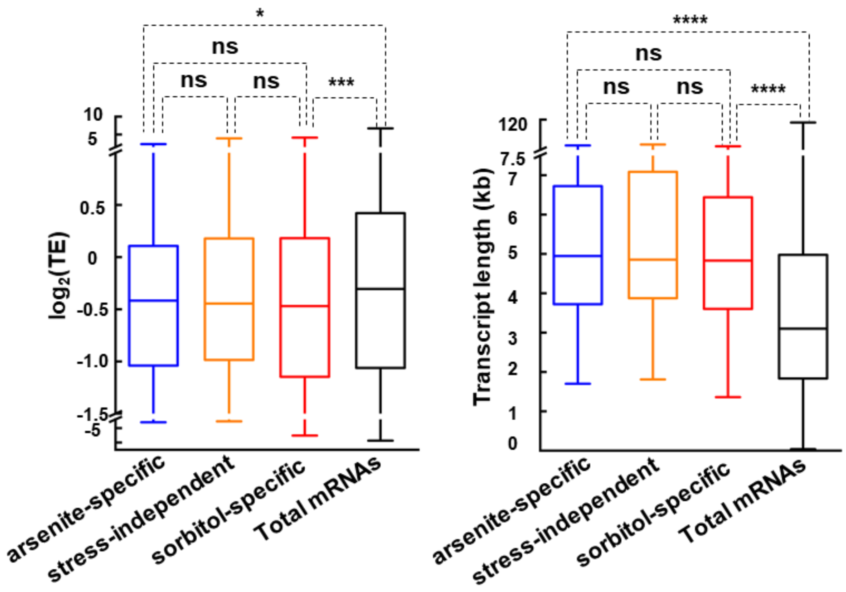

Supplementary Figure 19. Box plot comparing the translation efficiencies (left) and transcript length (right) in arsenite-specific, stress-independent, sorbitol-specific SG-proximal mRNAs in HEK293T cells. The boxes mark the first and third quartiles; whiskers indicate the minima and maxima; the central lines represent the median. An unpaired Mann-Whitney U test (two-sided) was performed to evaluate the statistical significance. ns, not significant ($p > 0.05$); * $p < 0.05$; ** $p < 0.01$; *** $p < 0.001$; **** $p < 0.0001$. Translation efficiencies are provided by a previous study[1]. AU content and transcript length features are referenced from Ensembl website.

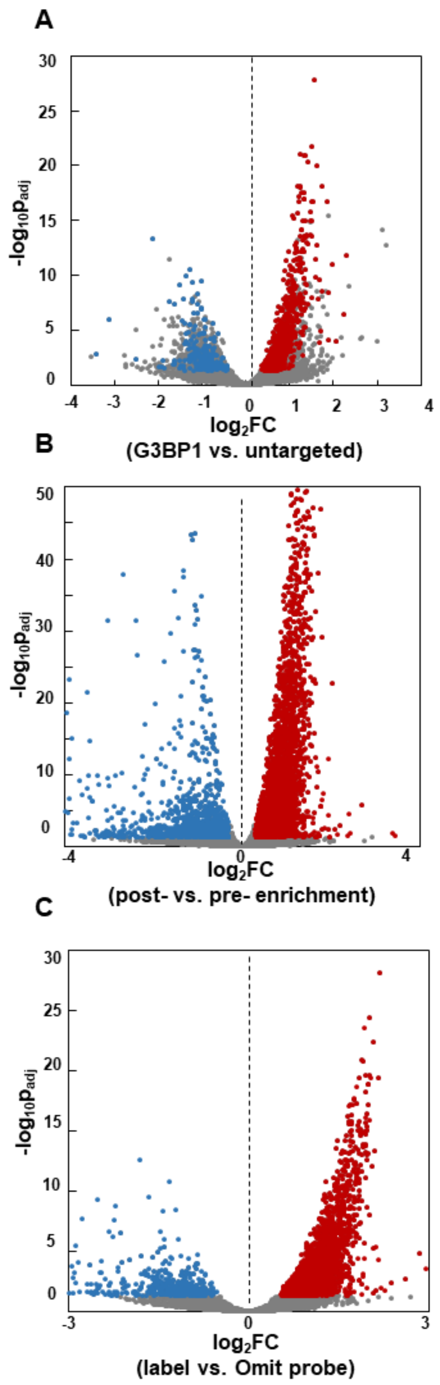

Supplementary Figure 20. Volcano plots showing enriched (red) and depleted (blue) RNAs in unstressed HEK293T cells. **(A)** G3BP1-miniSOG vs. untargeted miniSOG. Red and blue dots represent SG-proximal and SG-excluded transcripts captured by CAP-seq, respectively. **(B)** post- vs. pre-enrichment of G3BP1-miniSOG CAP-seq. **(C)** G3BP1-miniSOG labeling vs. negative control omitting PA.

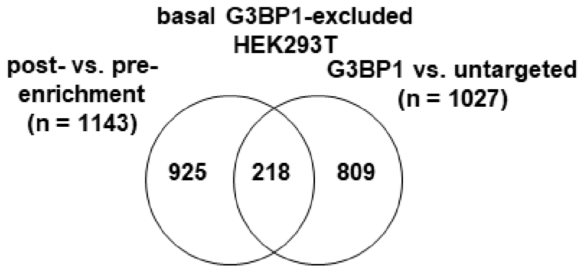

Supplementary Figure 21. Venn diagram comparing RNAs depleted in DESeq2 analysis between: 1) post- vs. pre-enrichment of G3BP1-miniSOG CAP-seq; 2) G3BP1-miniSOG vs. untargeted miniSOG. G3BP1-excluded RNAs in unstressed HEK293T are defined as the overlap of the two datasets.

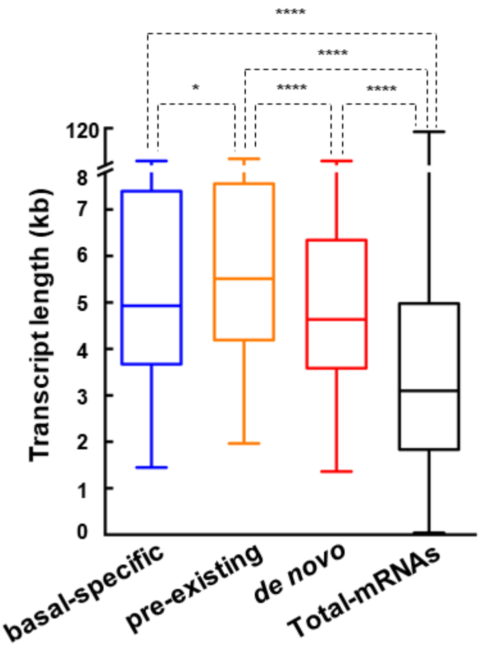

Supplementary Figure 22. Box plot comparing the transcript length in basal-specific, pre-existing and *de novo* mRNAs in HEK293T cells. ns, not significant (p > 0.05); The boxes mark the first and third quartiles; whiskers indicate the minima and maxima; the central lines represent the median. An unpaired Mann-Whitney test (two-sided) was performed to evaluate the statistical significance. * p < 0.05; **** p < 0.0001.Transcript length features are referenced from Ensembl website.

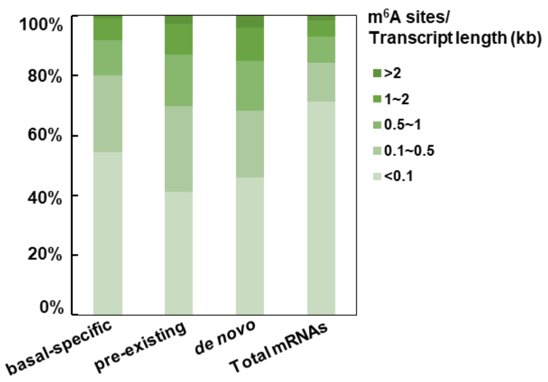

Supplementary Figure 23. Comparison of m^6A densities (m^6A sites per kilobase) in G3BP1-proximal mRNAs captured in basal-specific, pre-existing and *de novo* datasets.

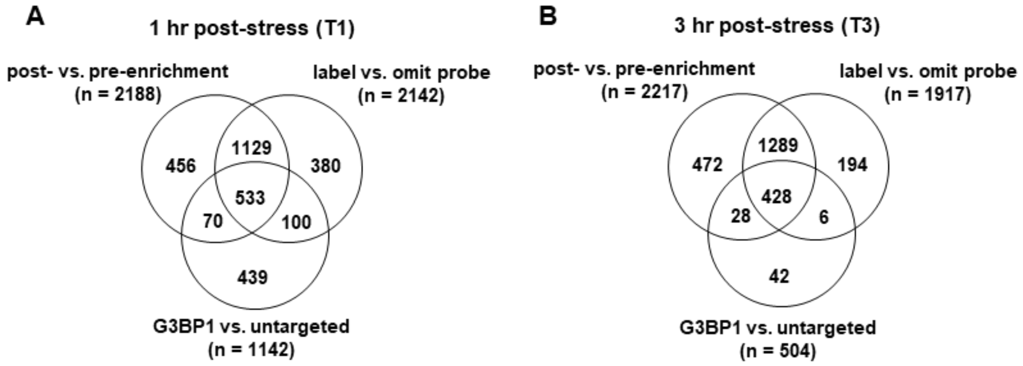

Supplementary Figure 24. Venn diagram comparing RNAs enriched in DESeq2 analysis between: 1) post- versus pre-enrichment of RNA labeled with G3BP1-miniSOG; 2) RNA labeled with G3BP1-miniSOG versus RNA from negative control omitting PA; 3) RNA labeled with G3BP1-miniSOG versus untargeted miniSOG. G3BP1-proximal RNAs under T1 **(A)** and T3 **(B)** in HEK293T cells are defined as the overlap of the three datasets.

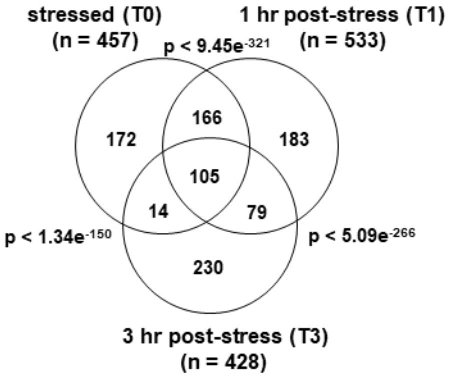

Supplementary Figure 25. Venn diagram comparing the CAP-seq defined SG-proximal dataset under T0 and G3BP1-proximal datasets under T1 and T3. p values were calculated by hypergeometric test.

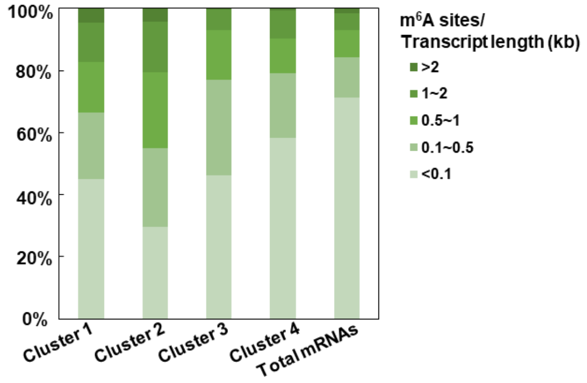

Supplementary Figure 26. Comparison of m^6A densities (m^6A sites per kilobase) in SG-proximal mRNAs across 4 clusters.
