# Supplementary material for: Profiling stress-triggered RNA condensation with photocatalytic proximity labeling
Source: Nat Commun. 2023 Nov 15;14:7390. doi: 10.1038/s41467-023-43194-2 (PMC10651888; doi:10.1038/s41467-023-43194-2)
Supplement: Supplementary file 3 — Description of Additional Supplementary Files [file 41467_2023_43194_MOESM3_ESM.pdf]

Description of Additional Supplementary Files

File Name: Supplementary Data 1
Description: DESeq2 analysis of HEK293T treated with arenite stress, sorbitol stress, without stress, and recovered 1 hr or 3 hr post arenite stress.

File Name: Supplementary Data 2
Description: DESeq2 analysis of U-2 OS cells treated with arsenite stress and mRNA features used for statistical analysis between CAPseq datasets in U-2 OS.

File Name: Supplementary Data 3
Description: mRNA features used for statistical analysis between CAPseq datasets in HEK293T.

File Name: Supplementary Data 4
Description: sequence of smFISH targets.
