# Supplementary material for: Profiling stress-triggered RNA condensation with photocatalytic proximity labeling
Source: Nat Commun. 2023 Nov 15;14:7390. doi: 10.1038/s41467-023-43194-2 (PMC10651888; doi:10.1038/s41467-023-43194-2)
Supplement: Supplementary file 8 — Reporting Summary [file 41467_2023_43194_MOESM8_ESM.pdf]

nature portfolio

Corresponding author(s): Peng Zou

Last updated by author(s): Oct 24, 2023

Reporting Summary

Nature Portfolio wishes to improve the reproducibility of the work that we publish. This form provides structure for consistency and transparency in reporting. For further information on Nature Portfolio policies, see our Editorial Policies and the Editorial Policy Checklist.

Statistics

For all statistical analyses, confirm that the following items are present in the figure legend, table legend, main text, or Methods section.

n/a	Confirmed	
☐	☒	The exact sample size (*n*) for each experimental group/condition, given as a discrete number and unit of measurement
☐	☒	A statement on whether measurements were taken from distinct samples or whether the same sample was measured repeatedly
☐	☒	The statistical test(s) used AND whether they are one- or two-sided<br>*Only common tests should be described solely by name; describe more complex techniques in the Methods section.*
☐	☒	A description of all covariates tested
☐	☒	A description of any assumptions or corrections, such as tests of normality and adjustment for multiple comparisons
☐	☒	A full description of the statistical parameters including central tendency (e.g. means) or other basic estimates (e.g. regression coefficient) AND variation (e.g. standard deviation) or associated estimates of uncertainty (e.g. confidence intervals)
☐	☒	For null hypothesis testing, the test statistic (e.g. *F*, *t*, *r*) with confidence intervals, effect sizes, degrees of freedom and *P* value noted<br>*Give P values as exact values whenever suitable.*
☒	☐	For Bayesian analysis, information on the choice of priors and Markov chain Monte Carlo settings
☒	☐	For hierarchical and complex designs, identification of the appropriate level for tests and full reporting of outcomes
☒	☐	Estimates of effect sizes (e.g. Cohen's *d*, Pearson's *r*), indicating how they were calculated

Our web collection on statistics for biologists contains articles on many of the points above.

Software and code

Policy information about availability of computer code

Data collection	Lab view 2015; Image lab.
Data analysis	The adaptors sequence in reads were removed by Cutadapt (v.1.18) and quality controlled by FastQC (v0.11.8). Sequencing reads were mapped by hisat2 (v2.1.0) . The mapped reads were counted by htseq-count (v0.7.2). Differential analysis were performed by R package DESeq2 (v1.34.0) and volcano plots were generated by Excel 2019. Heatmap was generated by R script pheatmap (v1.0.12).Mann-Whitney test used in statistical analysis by OriginPro (2019). Quantification of smFISH results were used by a script in MATLAB v9.10.0.1602886. (R2021a).

For manuscripts utilizing custom algorithms or software that are central to the research but not yet described in published literature, software must be made available to editors and reviewers. We strongly encourage code deposition in a community repository (e.g. GitHub). See the Nature Portfolio guidelines for submitting code & software for further information.

Data

Policy information about availability of data

All manuscripts must include a data availability statement. This statement should provide the following information, where applicable:

- Accession codes, unique identifiers, or web links for publicly available datasets
- A description of any restrictions on data availability
- For clinical datasets or third party data, please ensure that the statement adheres to our policy

Raw data files and processed data files have been uploaded to GEO with accession number GSE223295. GRCh38 (hg38) is available at https://ftp.ensembl.org/pub/release-87/gtf/homo_sapiens/. For smFISH analysis and SG co-localization analysis script in MATLAB, go to https://github.com/PKUCHEMZouLab/CAP-seq_stress-granlue. The raw data of uncropped gel and bar charts are provided in the Source Data file.

Research involving human participants, their data, or biological material

Policy information about studies with human participants or human data. See also policy information about sex, gender (identity/presentation), and sexual orientation and race, ethnicity and racism.

Reporting on sex and gender	not related
Reporting on race, ethnicity, or other socially relevant groupings	not related
Population characteristics	not related
Recruitment	not related
Ethics oversight	not related

Note that full information on the approval of the study protocol must also be provided in the manuscript.

Field-specific reporting

Please select the one below that is the best fit for your research. If you are not sure, read the appropriate sections before making your selection.

☒ Life sciences ☐ Behavioural & social sciences ☐ Ecological, evolutionary & environmental sciences

For a reference copy of the document with all sections, see nature.com/documents/nr-reporting-summary-flat.pdf

Life sciences study design

All studies must disclose on these points even when the disclosure is negative.

Sample size	No sample size calculation was performed. Each biological replicate of HEK293T and U-2 OS cells under basal, arsenite stress and sorbitol stress were from one 15-cm dish for next-generation sequencing; Each biological replicate for HEK293T cells under 1 hr or 3 hr post arsenite were from one 10-cm dish for next-generation sequencing. Sample size were chosen based on the statistical analysis in differential analysis by DESeq2.Western bolt used one cell of six-well plate. Immunofluorescence and smFISH used one cell of 24-well plate of HEK293T or U-2 OS cells.
Data exclusions	No data were excluded.
Replication	HEK293T stably expressing G3BP1-miniSOG under basal, arsenite stress, sorbitol stress or 3 hr post-arsentie stress were prepared in two biological replicates for sequencing; HEK293T stably expressing G3BP1-miniSOG under 1 hr post-arsentie stress were prepared in three biological replicates for sequencing; HEK293T stably expressing untargeted miniSOG were prepared in four replicates. U-2 OS cells stably expressing untargeted miniSOG and G3BP1-miniSOG were prepared in two replicates. Replication were chosen for statistical analysis. Western blot and imaging experiments were taken for at least 3 replicates. The replicates of each experiment showed good reproducibility.
Randomization	Cells were from one dish of cells of the previous generation then were randomly distributed to each condition.
Blinding	NGS sample preparation and analysis were performed by same experimenter and blind checked by another experimenter. The NGS instrument operator was not informed of the details of experimental design (control experiments, sample preparation workflow, etc.), thus providing an unbiased analysis of the sequencing data.

Reporting for specific materials, systems and methods

We require information from authors about some types of materials, experimental systems and methods used in many studies. Here, indicate whether each material, system or method listed is relevant to your study. If you are not sure if a list item applies to your research, read the appropriate section before selecting a response.

Materials & experimental systems

n/a	Involved in the study
☐	☒ Antibodies
☐	☒ Eukaryotic cell lines
☒	☐ Palaeontology and archaeology
☒	☐ Animals and other organisms
☒	☐ Clinical data
☒	☐ Dual use research of concern
☒	☐ Plants

Methods

n/a	Involved in the study
☒	☐ ChIP-seq
☒	☐ Flow cytometry
☒	☐ MRI-based neuroimaging

Antibodies

Antibodies used

mouse anti-V5, V5-Tag Monoclonal Antibody(3C8), (Biodragon, B1005, 1:1000 for IF)
rabbit anti-G3BP2, Rabbit polyclonal to G3BP2 (abcam, ab86135, 1:200 for IF)
rabbit anti-TIA1, Rabbit monoclonal [EPR9304] to TIA1, (abcam, ab140595, 1:200 for IF)
rabbit anti-G3BP, Rabbit monoclonal [EPR13986(B)] to G3BP, (abcam, ab181150, 1:1000 for Western blot)
Goat anti-mouse-Alexa Fluor 488, (ThermoFisher, A-11029, 1:1000 for IF)
Goat anti-rabbit-Alexa Fluor 568, (ThermoFisher, A-11011, 1:1000 for IF)
Streptavidin-Alexa Fluor 647, (ThermoFisher, S21374, 1:1000-2000 for IF)
Streptavidin-Alexa Fluor 568, (ThermoFisher, S11225, 1:1000-2000 for IF)
Goat anti-rabbit-Alexa Fluor 647, (ThermoFisher, A-21244, 1:1000 for IF)
Rabbit Anti-Goat IgG H&L (HRP), (Biodragon, BF03008, 1:4000 for Western blot)
rabbit anti-eIF2α, eIF2α (D7D3) XP® Rabbit mAb, (CST, 5324, 1:1000 for Western blot)
rabbit-anti-EIF2S1 (phospho S51), Rabbit monoclonal [E90] to EIF2S1 (phospho S51), (abcam, ab32157, 1:1000 for Western blot)

Validation

All antibodies were validated by manufacturer and validation are available from the website. mouse anti-V5 https://www.biodragon.cn/plus/view.php?aid=74653;rabbit anti-G3BP2 https://www.abcam.cn/products/primary-antibodies/g3bp2-antibody-ab86135.html; rabbit anti-TIA1 https://www.abcam.cn/products/primary-antibodies/tia1-antibody-epr9304-ab140595.html; rabbit anti-G3BP https://abcam.cn/products/primary-antibodies/g3bp-antibody-epr13986b-ab181150.html; Goat anti-mouse-Alexa Fluor 488 https://www.thermofisher.cn/cn/zh/antibody/product/Goat-anti-Mouse-IgG-H-L-Highly-Cross-Adsorbed-Secondary-Antibody-Polyclonal/A-11029; Goat anti-rabbit-Alexa Fluor 568 https://www.thermofisher.cn/cn/zh/antibody/product/Goat-anti-Rabbit-IgG-H-L-Cross-Adsorbed-Secondary-Antibody-Polyclonal/A-11011; Streptavidin-Alexa Fluor 647 https://www.thermofisher.cn/order/catalog/product/S21374?SID=srch-srp-S21374; Streptavidin-Alexa Fluor 568 https://www.thermofisher.cn/order/catalog/product/S11226?SID=srch-srp-S11226; Goat anti-rabbit-Alexa Fluor 647 https://www.thermofisher.cn/cn/zh/antibody/product/Goat-anti-Rabbit-IgG-H-L-Cross-Adsorbed-Secondary-Antibody-Polyclonal/A-21244; rabbit-anti-EIF2S1 (phospho S51) https://www.abcam.cn/products/primary-antibodies/eif2s1-phospho-s51-antibody-e90-ab32157.html; Rabbit Anti-Goat IgG H&L (HRP) https://www.biodragon.cn/hrpbjek/74422.html; rabbit anti-eIF2α https://www.cellsignal.cn/products/primary-antibodies/eif2a-d7d3-xp-rabbit-mab/5324;

Eukaryotic cell lines

Policy information about cell lines and Sex and Gender in Research

Cell line source(s)

HEK293T cells were from American Type Culture Collection (ATCC)
HEK293T/17 cells were from National Science & Technology Infrastructure--National BioMedical Cell-Line Resource (NSTI-BMCR)
U-2 OS cells were from National Science & Technology Infrastructure--National BioMedical Cell-Line Resource (NSTI-BMCR)
Stable cell lines were generated by lenti virus infection.

Authentication

Stable cells expressing fusion protein were confirmed by fluorescence imaging and Western blot.

Mycoplasma contamination

Cell lines are PCR-tested positive for mycoplasma contamination.

Commonly misidentified lines
(See ICLAC register)

No commonly misidentified lines were used in this study.

Plants

Seed stocks	This study does not involve plate samples.
Novel plant genotypes	This study does not involve plate samples.
Authentication	This study does not involve plate samples.
