# Supplementary figures and images for: Profiling stress-triggered RNA condensation with photocatalytic proximity labeling
Source: Nat Commun. 2023 Nov 15;14:7390. doi: 10.1038/s41467-023-43194-2 (PMC10651888; doi:10.1038/s41467-023-43194-2)

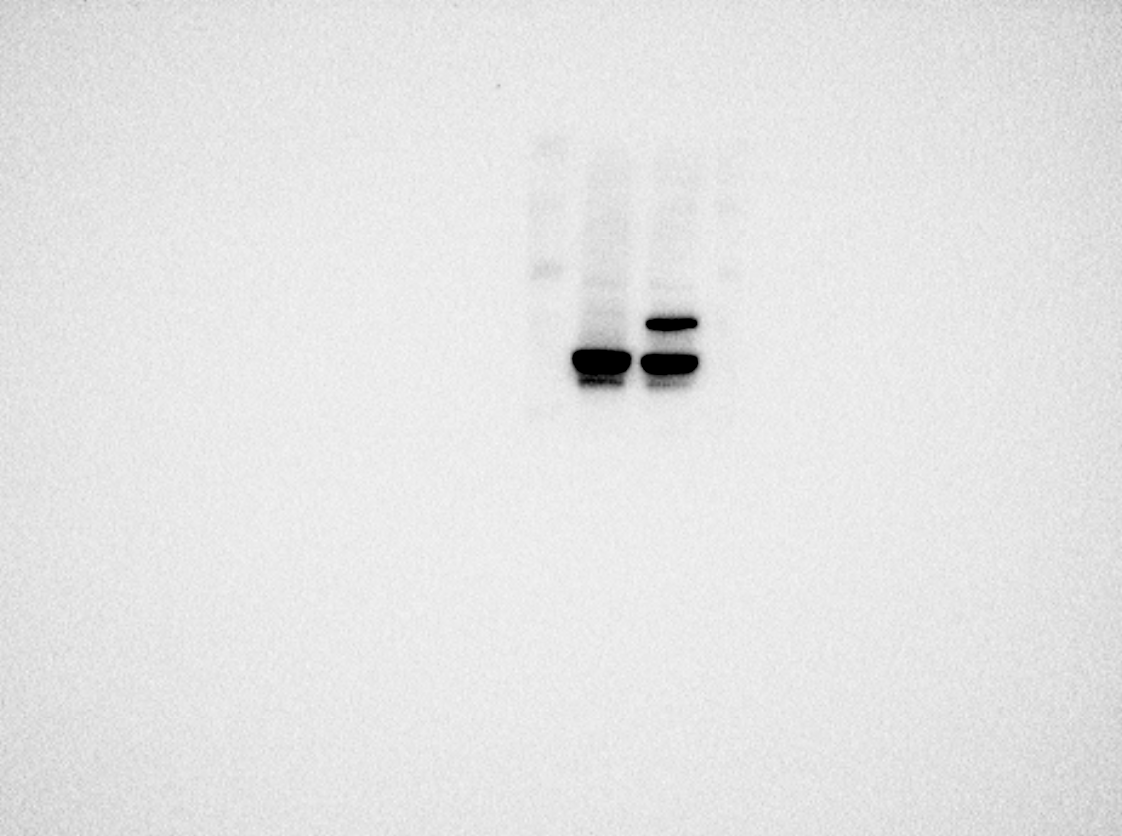

Supplement: Supplementary file 9 — Source Data File [file 41467_2023_43194_MOESM9_ESM.zip › Source Data File/Raw Data of Western blot/Supplementary Figure 1/replicate 1-display in Supplementary Figure 1/G3BP1/HEK293T_WT-G3BP1-mS HEK293T.tif]

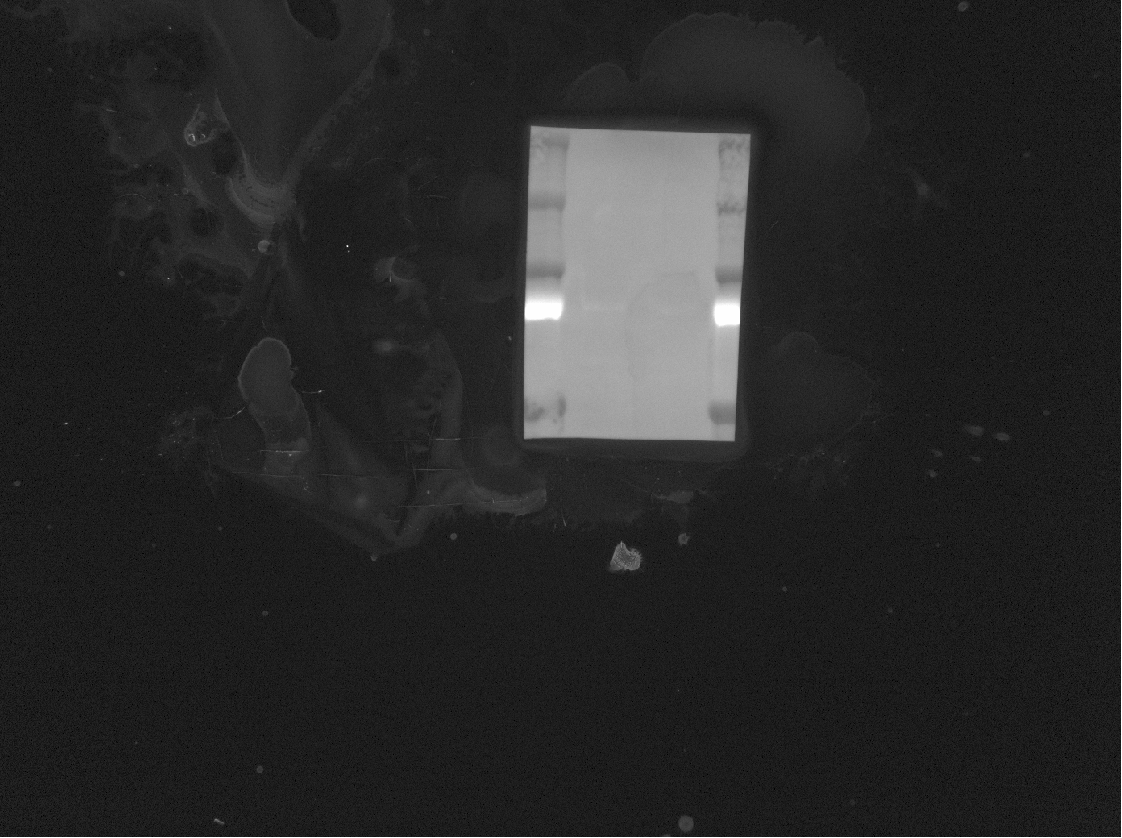

Supplement: Supplementary file 9 — Source Data File [file 41467_2023_43194_MOESM9_ESM.zip › Source Data File/Raw Data of Western blot/Supplementary Figure 1/replicate 1-display in Supplementary Figure 1/G3BP1/marker.tif]

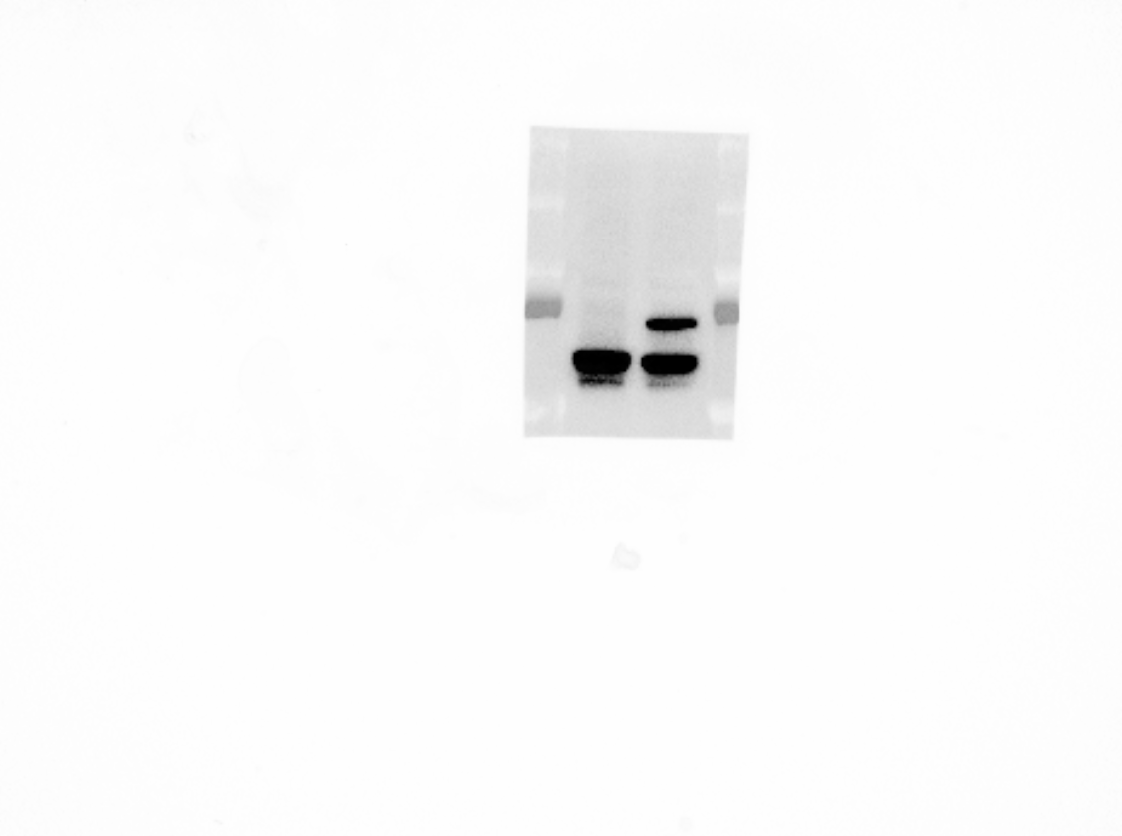

Supplement: Supplementary file 9 — Source Data File [file 41467_2023_43194_MOESM9_ESM.zip › Source Data File/Raw Data of Western blot/Supplementary Figure 1/replicate 1-display in Supplementary Figure 1/G3BP1/merge.tif]

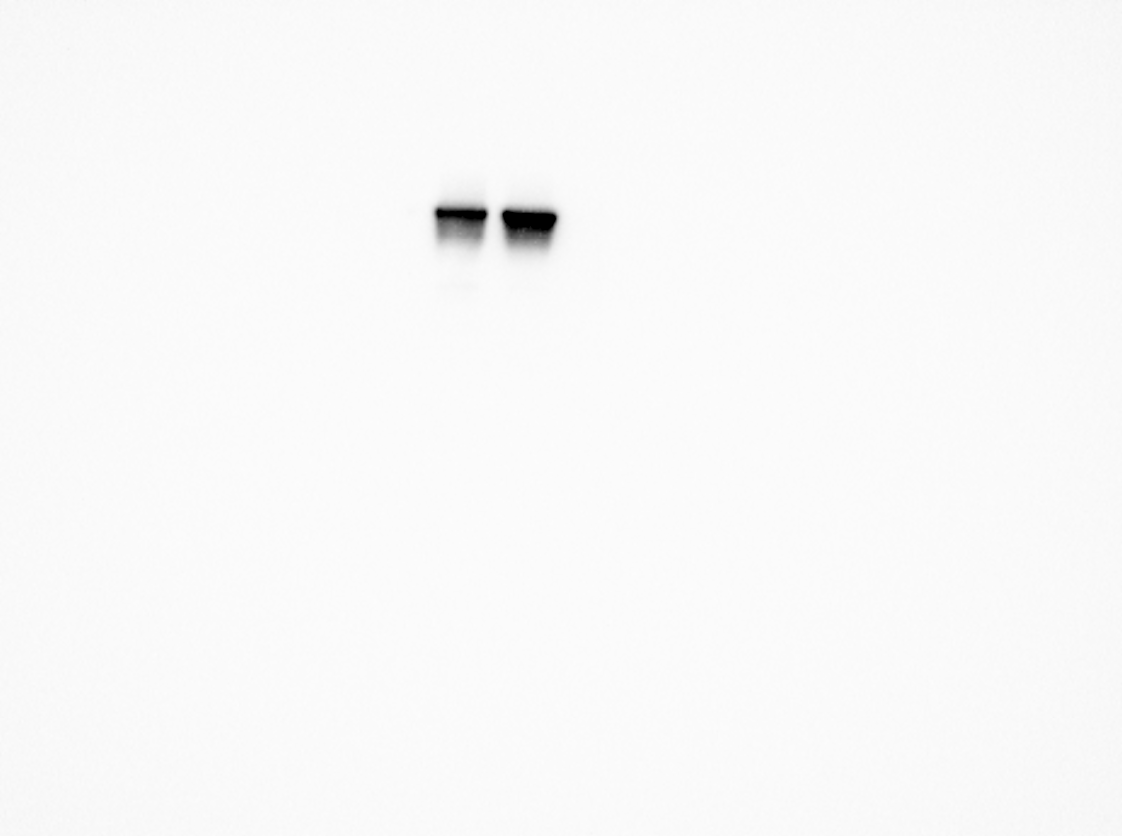

Supplement: Supplementary file 9 — Source Data File [file 41467_2023_43194_MOESM9_ESM.zip › Source Data File/Raw Data of Western blot/Supplementary Figure 1/replicate 1-display in Supplementary Figure 1/loading control-eIF2alpha/HEK293T_WT-G3BP1-mS HEK293T.tif]

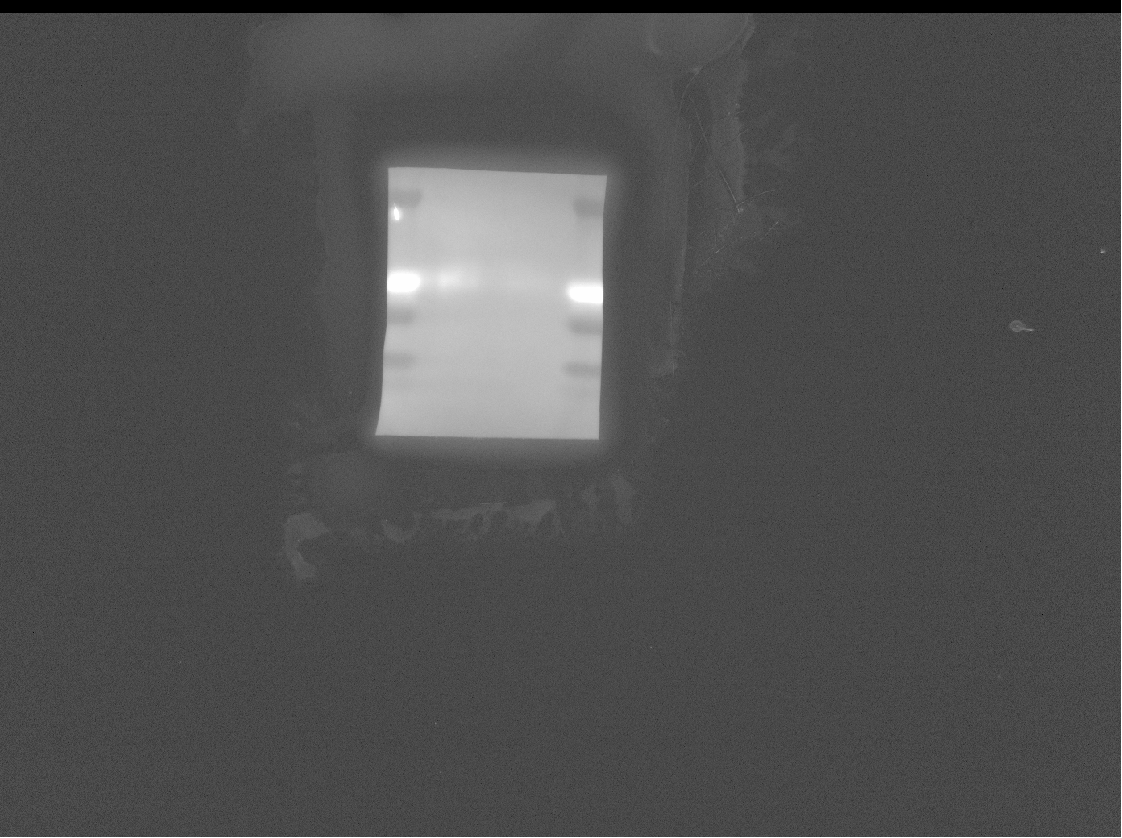

Supplement: Supplementary file 9 — Source Data File [file 41467_2023_43194_MOESM9_ESM.zip › Source Data File/Raw Data of Western blot/Supplementary Figure 1/replicate 1-display in Supplementary Figure 1/loading control-eIF2alpha/marker.tif]

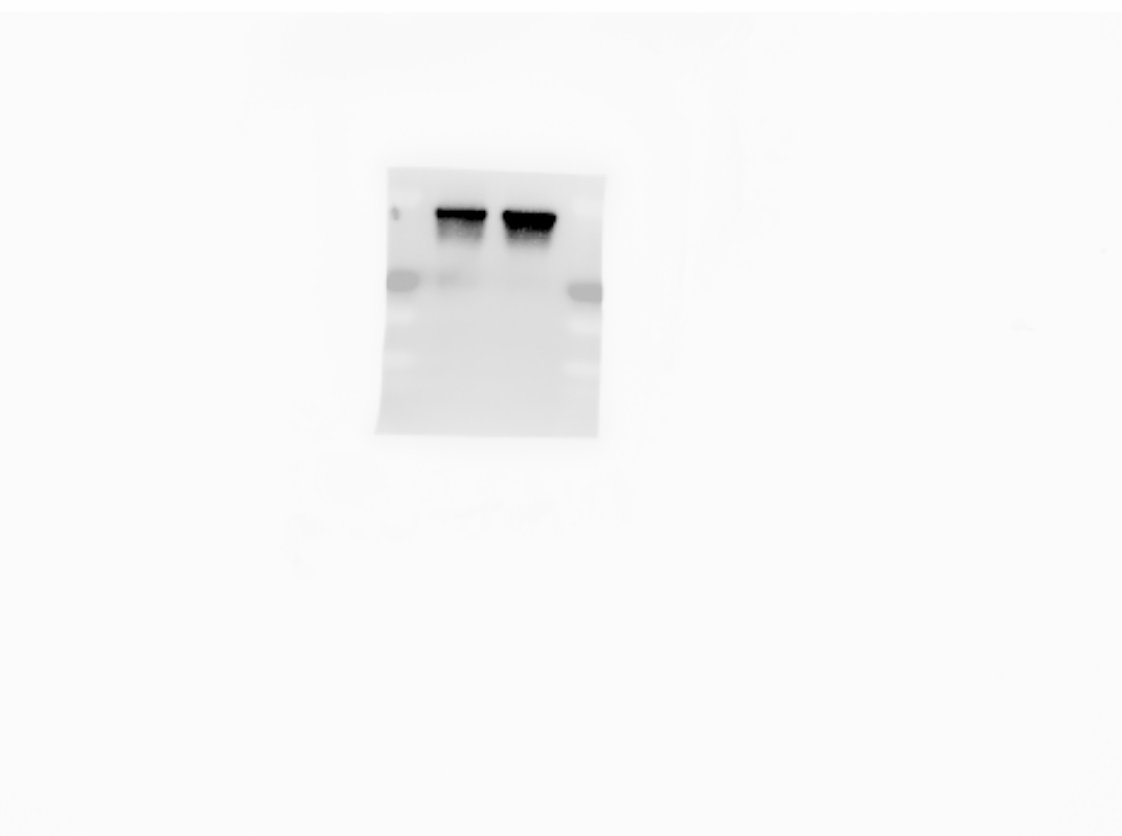

Supplement: Supplementary file 9 — Source Data File [file 41467_2023_43194_MOESM9_ESM.zip › Source Data File/Raw Data of Western blot/Supplementary Figure 1/replicate 1-display in Supplementary Figure 1/loading control-eIF2alpha/merge.tif]

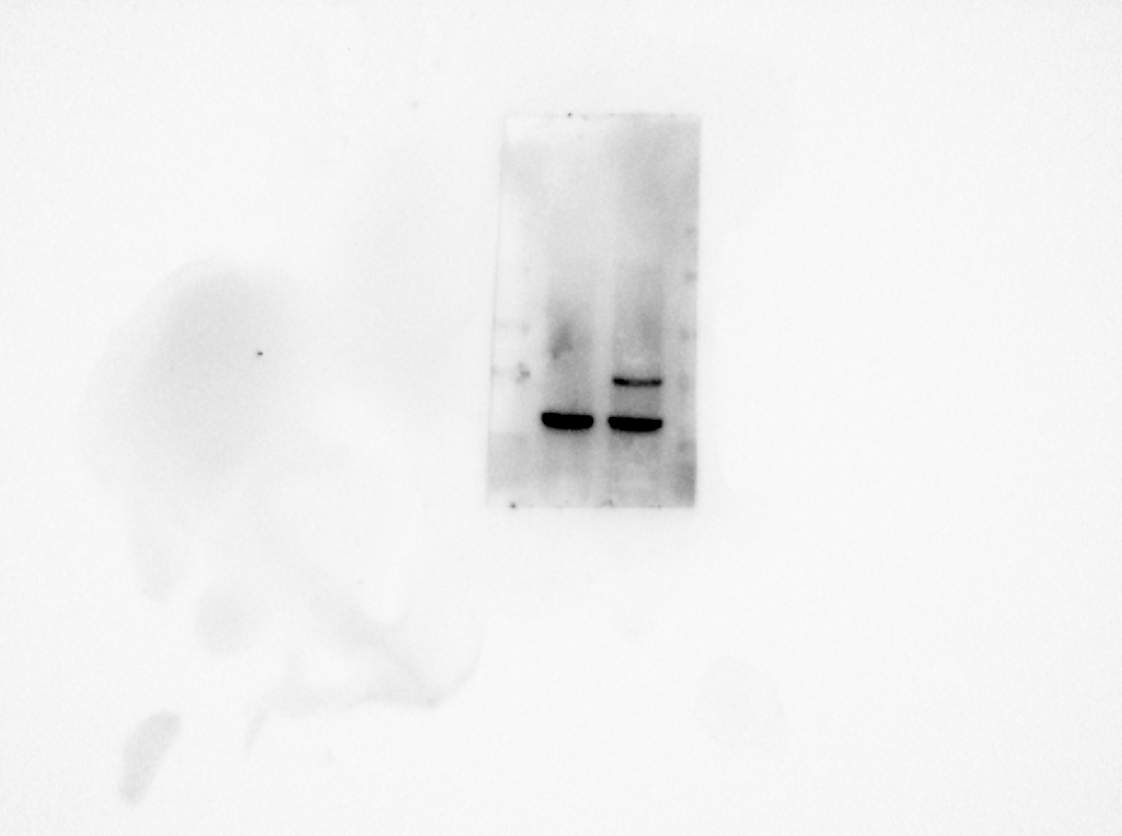

Supplement: Supplementary file 9 — Source Data File [file 41467_2023_43194_MOESM9_ESM.zip › Source Data File/Raw Data of Western blot/Supplementary Figure 1/replicate 2/G3BP1/HEK293T WT-G3BP1-mS HEK293T.tif]

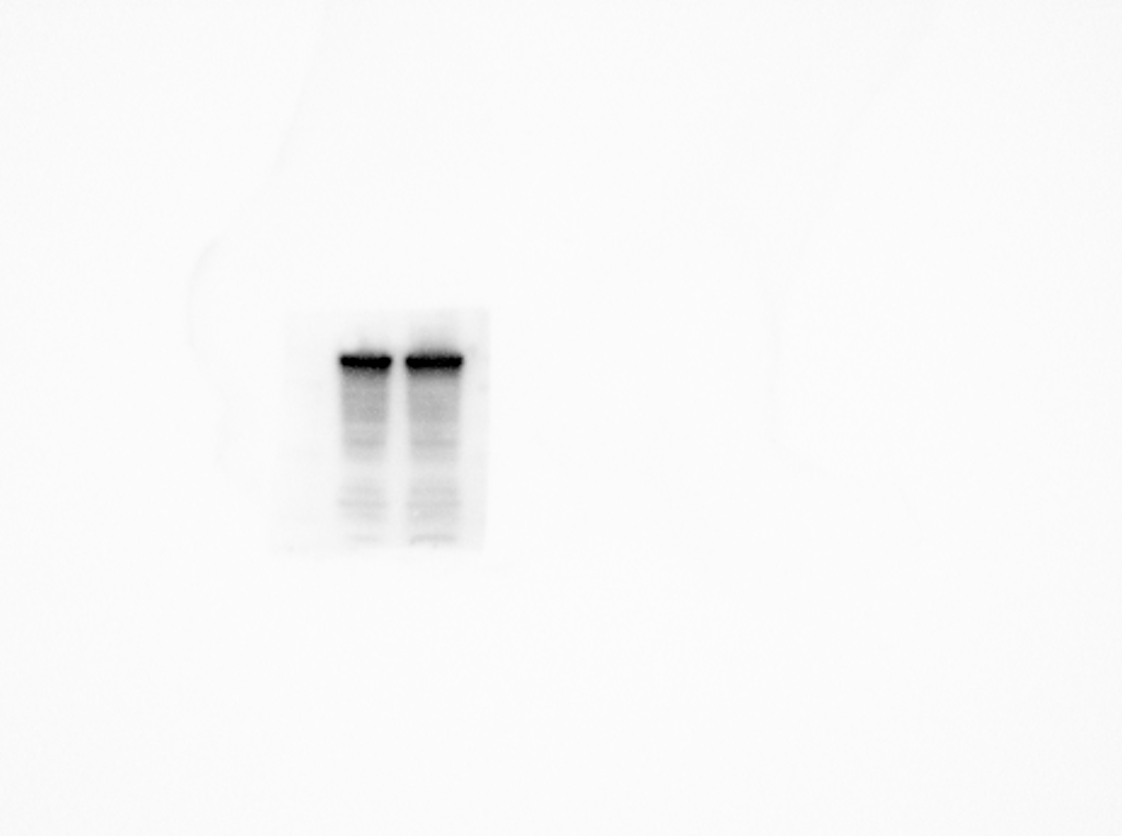

Supplement: Supplementary file 9 — Source Data File [file 41467_2023_43194_MOESM9_ESM.zip › Source Data File/Raw Data of Western blot/Supplementary Figure 1/replicate 2/loading control-eIF2alpha/HEK293T WT-G3BP1-mS HEK293T.tif]

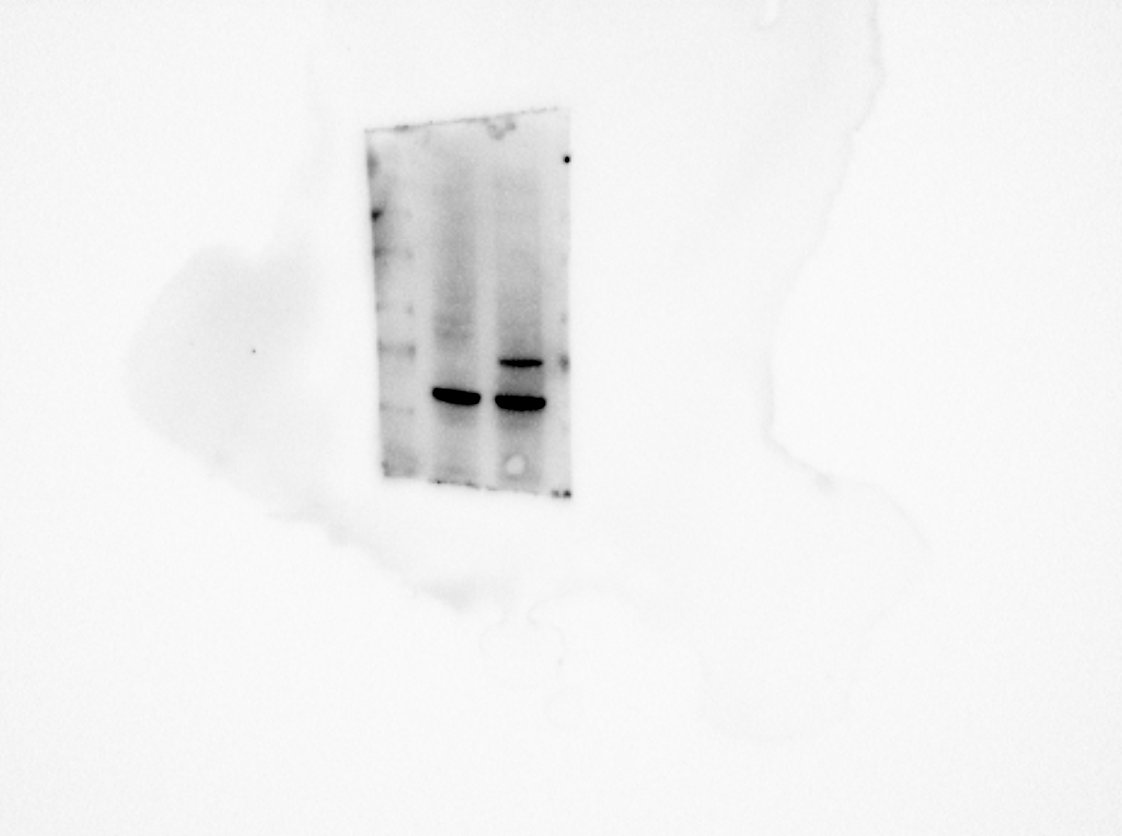

Supplement: Supplementary file 9 — Source Data File [file 41467_2023_43194_MOESM9_ESM.zip › Source Data File/Raw Data of Western blot/Supplementary Figure 1/replicate 3/G3BP1/HEK293T WT-G3BP1-mS HEK293T.tif]

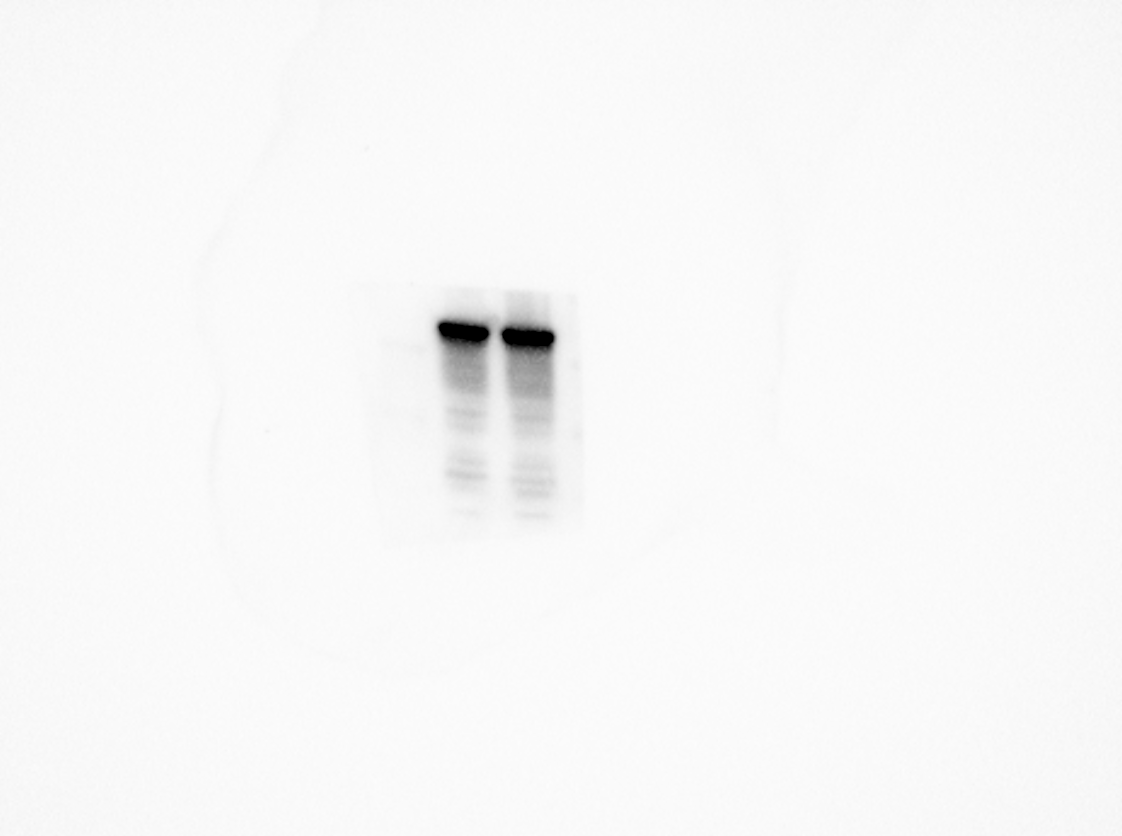

Supplement: Supplementary file 9 — Source Data File [file 41467_2023_43194_MOESM9_ESM.zip › Source Data File/Raw Data of Western blot/Supplementary Figure 1/replicate 3/loading control-eIF2alpha/HEK293T WT-G3BP1-mS HEK293T.tif]

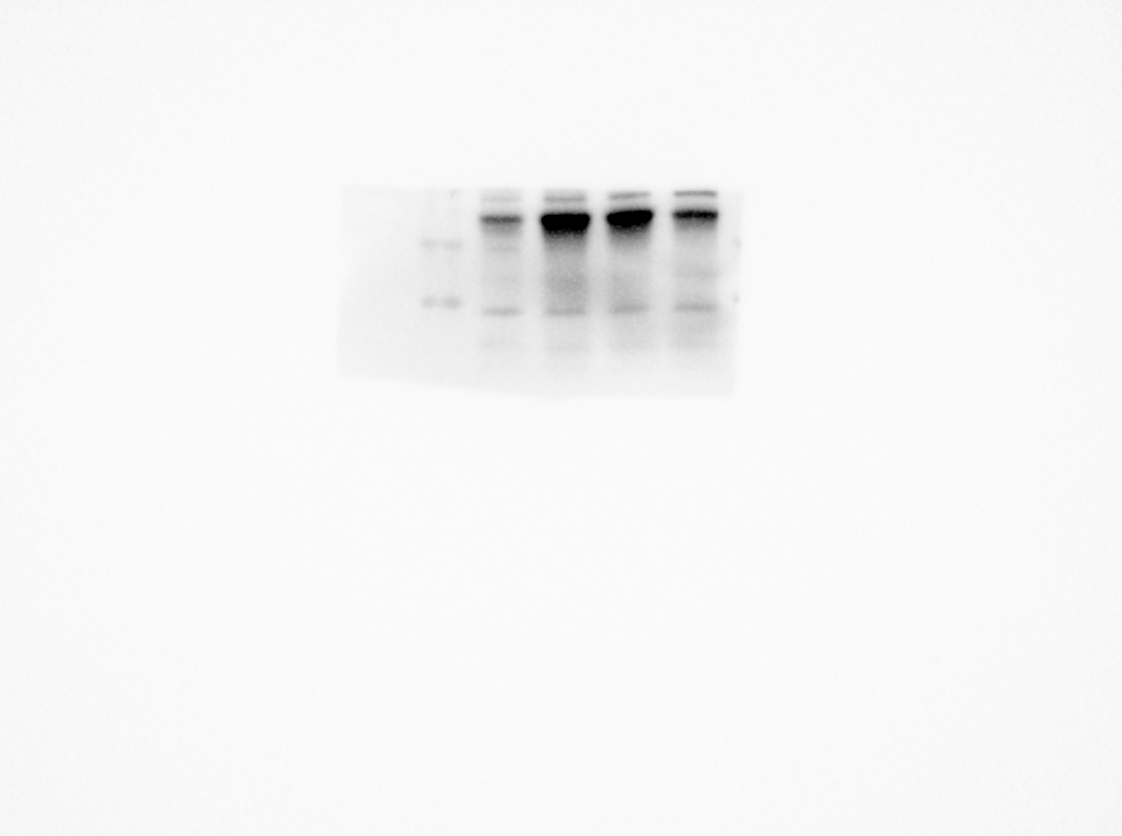

Supplement: Supplementary file 9 — Source Data File [file 41467_2023_43194_MOESM9_ESM.zip › Source Data File/Raw Data of Western blot/Supplementary Figure 2/replicate 1_display in Supplementary Figure2/eIF2alpha-phorspho/eIF2alpha/basal_T0_T1_T3.tif]

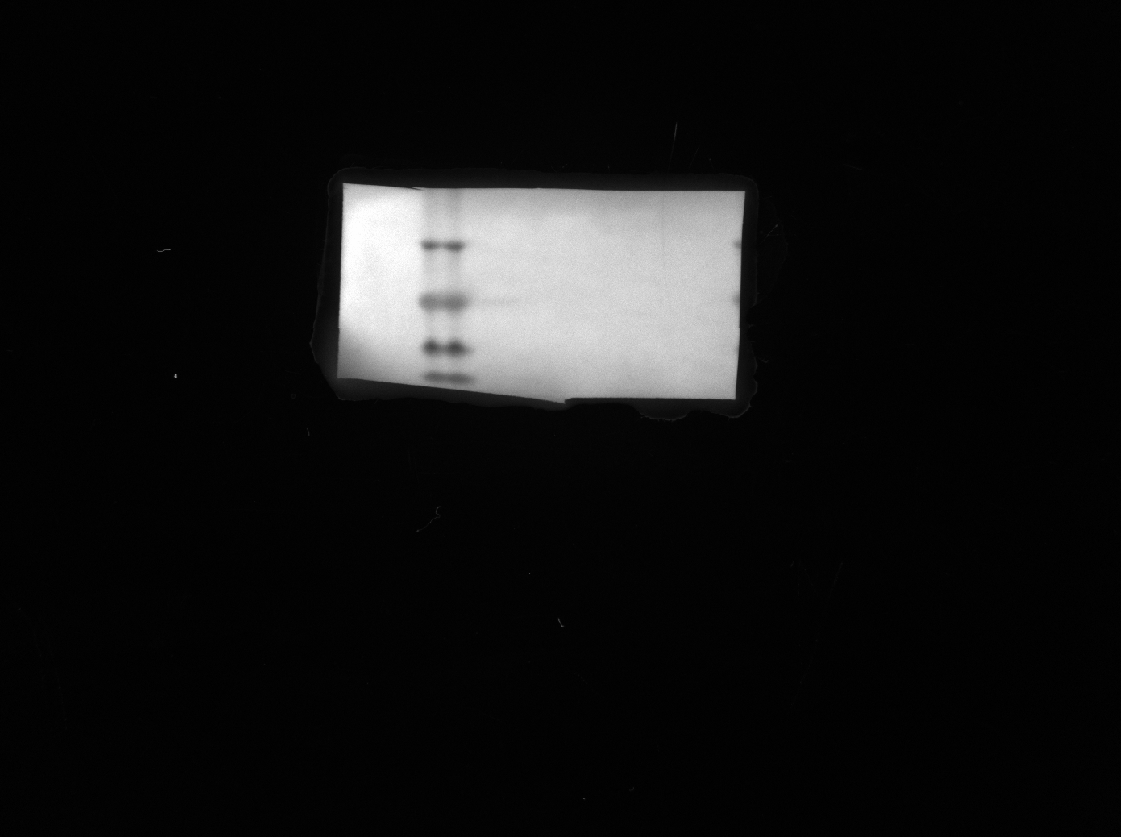

Supplement: Supplementary file 9 — Source Data File [file 41467_2023_43194_MOESM9_ESM.zip › Source Data File/Raw Data of Western blot/Supplementary Figure 2/replicate 1_display in Supplementary Figure2/eIF2alpha-phorspho/eIF2alpha/marker.tif]

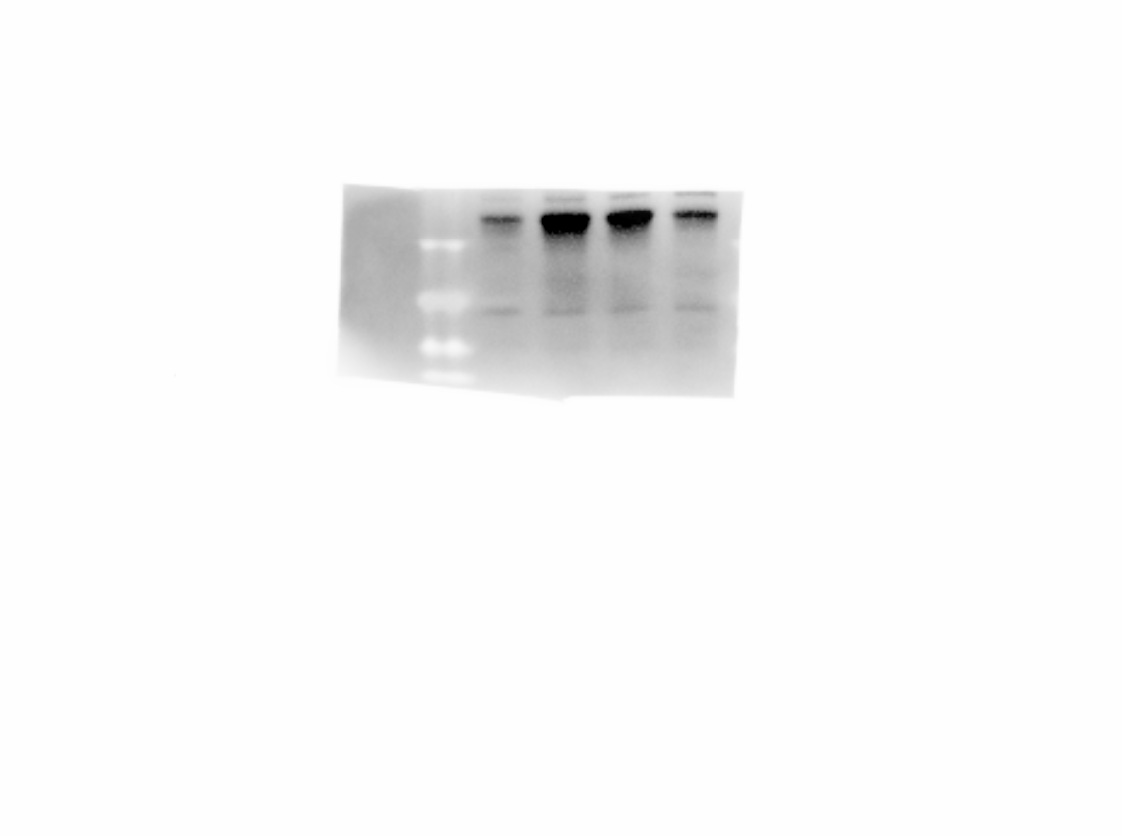

Supplement: Supplementary file 9 — Source Data File [file 41467_2023_43194_MOESM9_ESM.zip › Source Data File/Raw Data of Western blot/Supplementary Figure 2/replicate 1_display in Supplementary Figure2/eIF2alpha-phorspho/eIF2alpha/merge.tif]

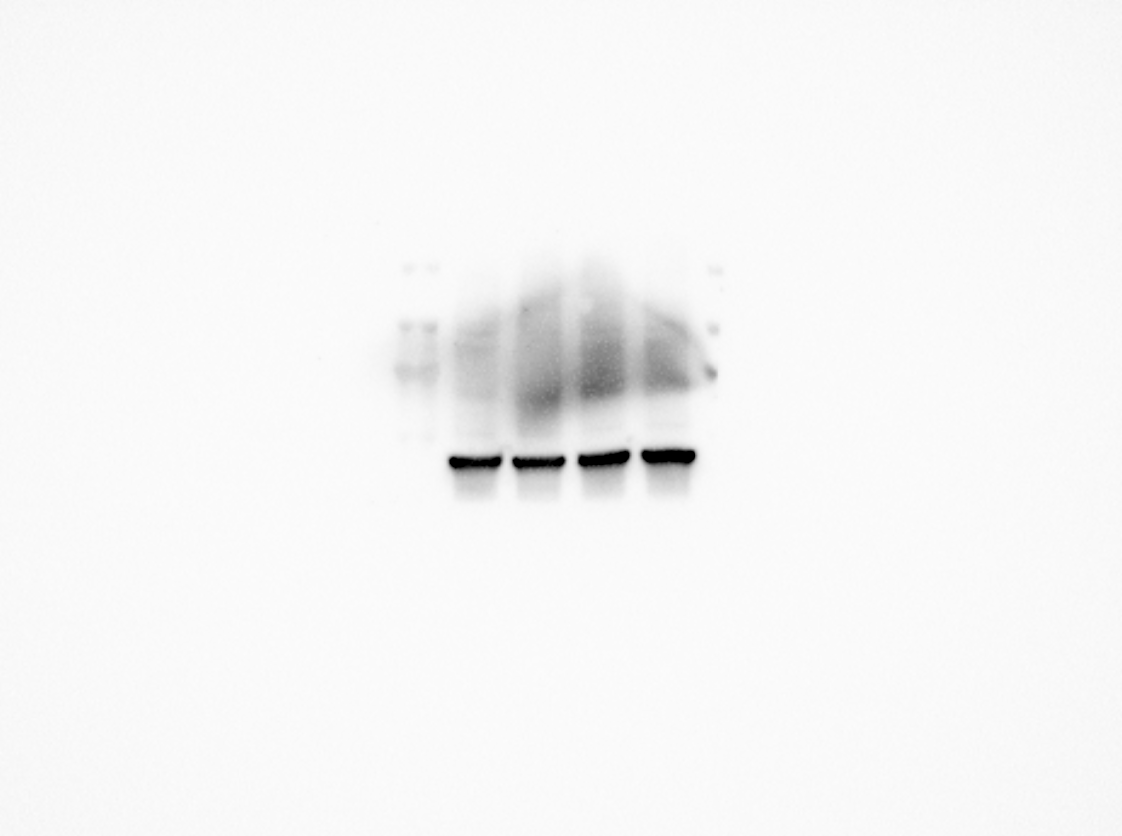

Supplement: Supplementary file 9 — Source Data File [file 41467_2023_43194_MOESM9_ESM.zip › Source Data File/Raw Data of Western blot/Supplementary Figure 2/replicate 1_display in Supplementary Figure2/eIF2alpha-phorspho/loading control-alpha Tubulin/basal_T0_T1_T3.tif]

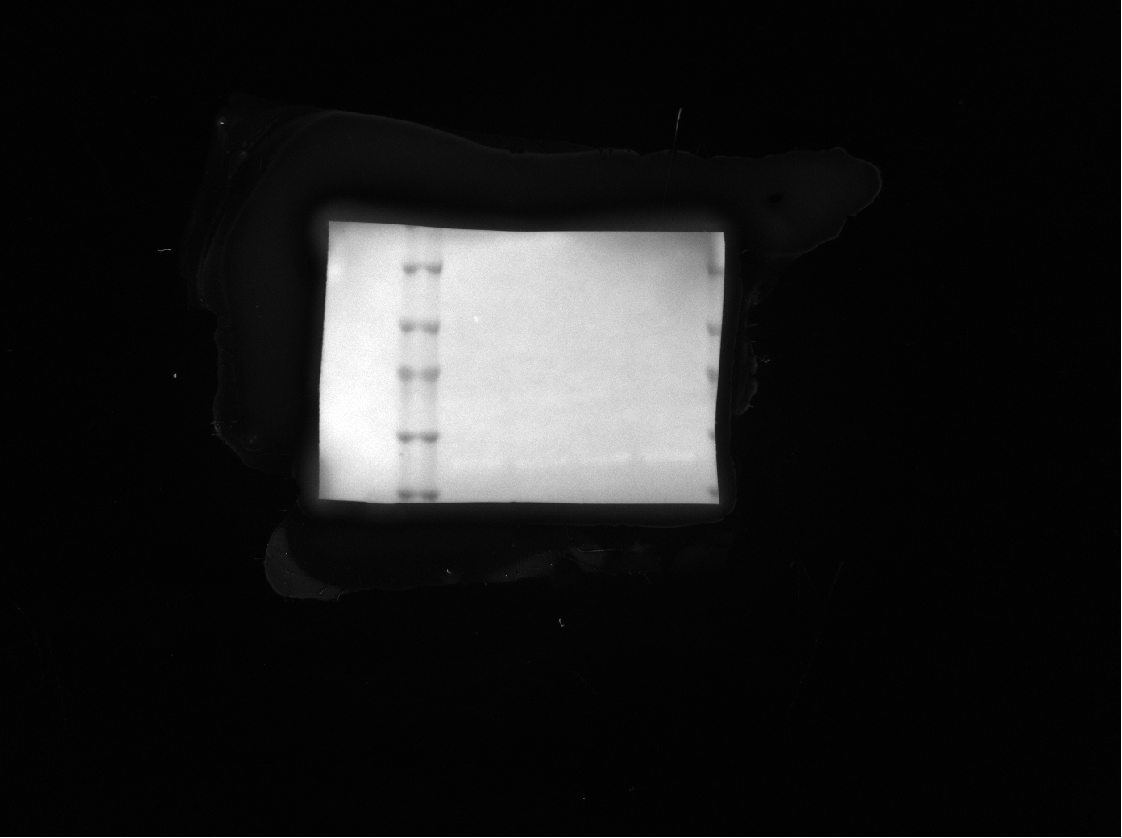

Supplement: Supplementary file 9 — Source Data File [file 41467_2023_43194_MOESM9_ESM.zip › Source Data File/Raw Data of Western blot/Supplementary Figure 2/replicate 1_display in Supplementary Figure2/eIF2alpha-phorspho/loading control-alpha Tubulin/marker_Tubulin.tif]

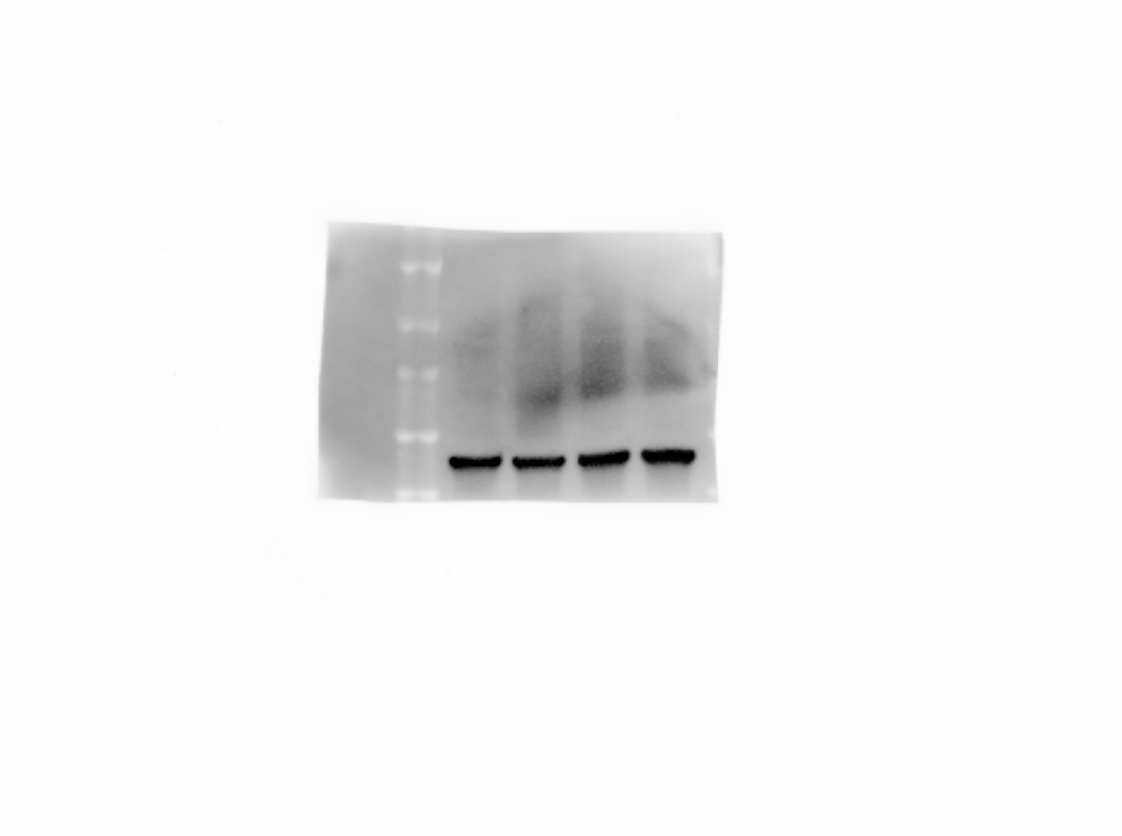

Supplement: Supplementary file 9 — Source Data File [file 41467_2023_43194_MOESM9_ESM.zip › Source Data File/Raw Data of Western blot/Supplementary Figure 2/replicate 1_display in Supplementary Figure2/eIF2alpha-phorspho/loading control-alpha Tubulin/merge.tif]

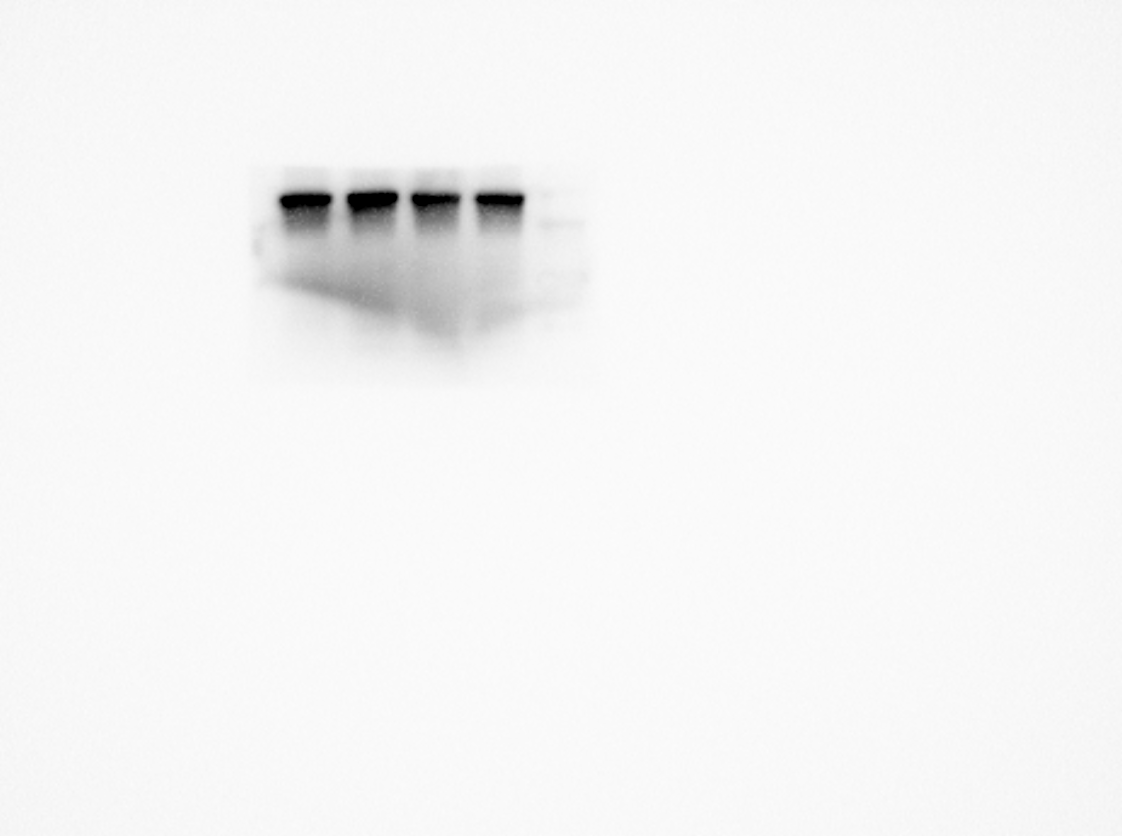

Supplement: Supplementary file 9 — Source Data File [file 41467_2023_43194_MOESM9_ESM.zip › Source Data File/Raw Data of Western blot/Supplementary Figure 2/replicate 1_display in Supplementary Figure2/eIF2alpha/eIF2alpha/basal_T0_T1_T3.tif]

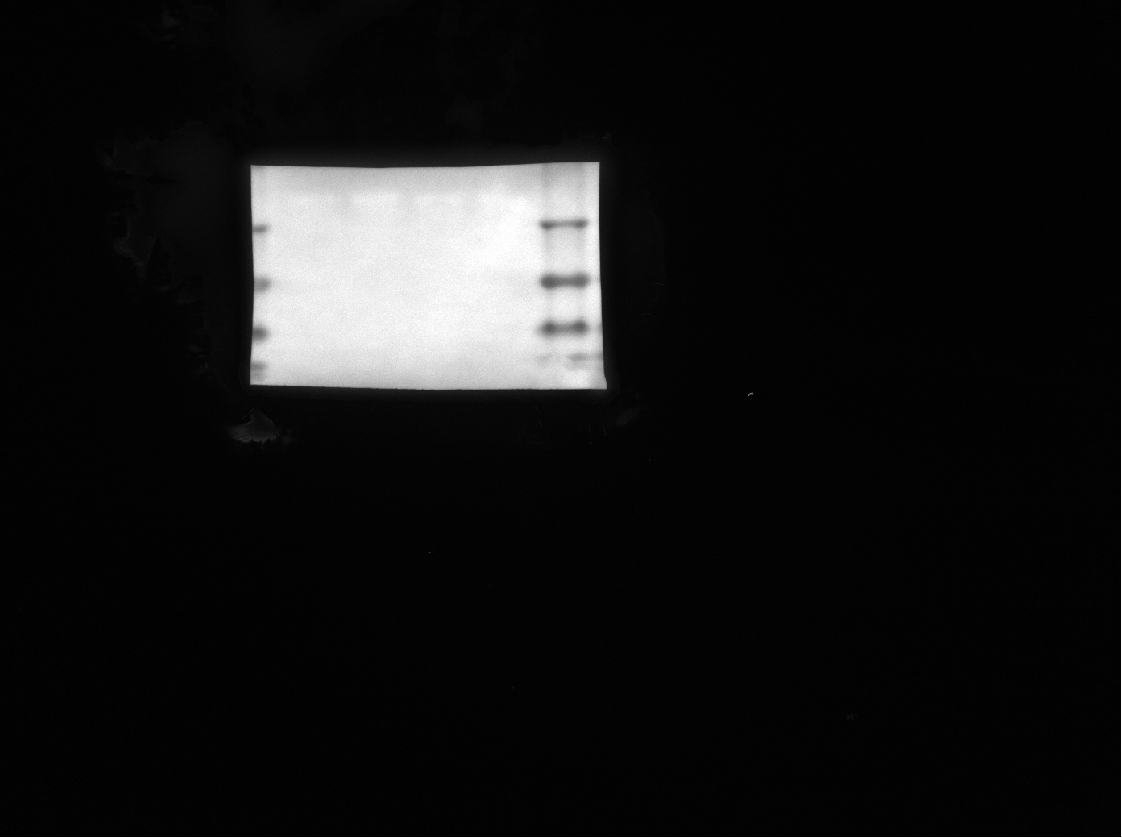

Supplement: Supplementary file 9 — Source Data File [file 41467_2023_43194_MOESM9_ESM.zip › Source Data File/Raw Data of Western blot/Supplementary Figure 2/replicate 1_display in Supplementary Figure2/eIF2alpha/eIF2alpha/marker.tif]

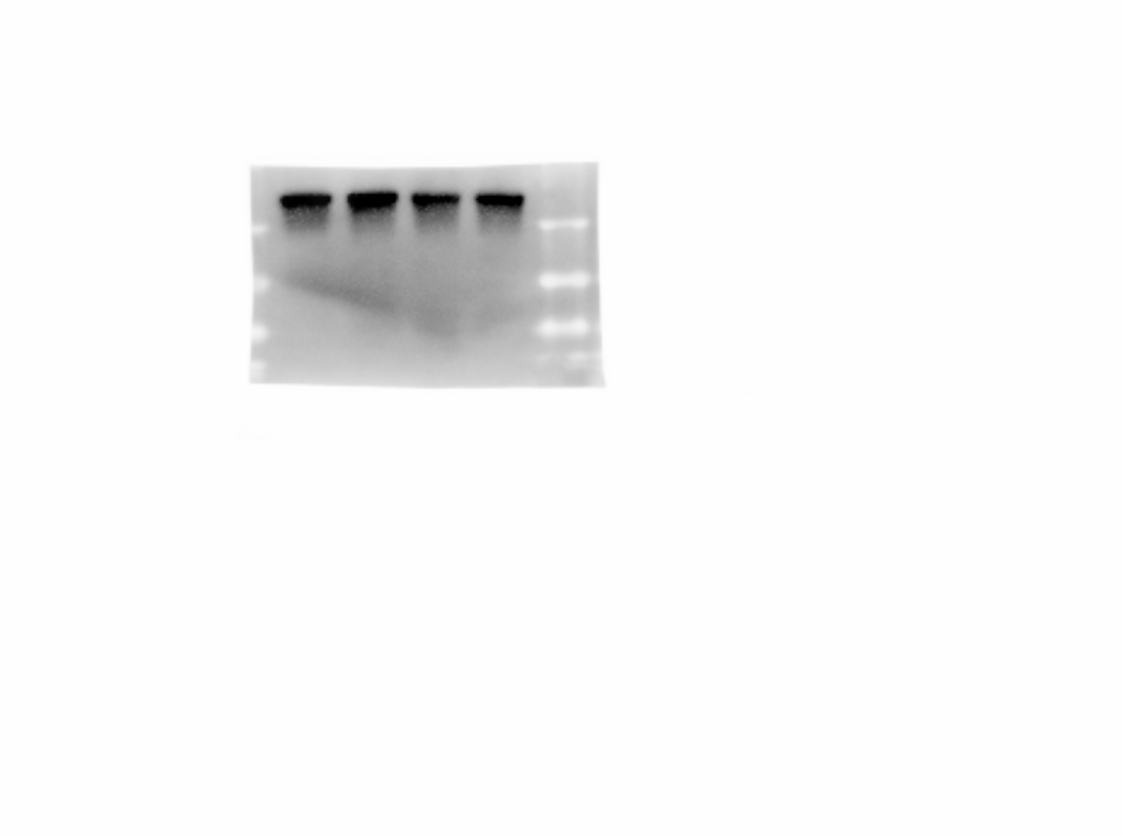

Supplement: Supplementary file 9 — Source Data File [file 41467_2023_43194_MOESM9_ESM.zip › Source Data File/Raw Data of Western blot/Supplementary Figure 2/replicate 1_display in Supplementary Figure2/eIF2alpha/eIF2alpha/merge.tif]

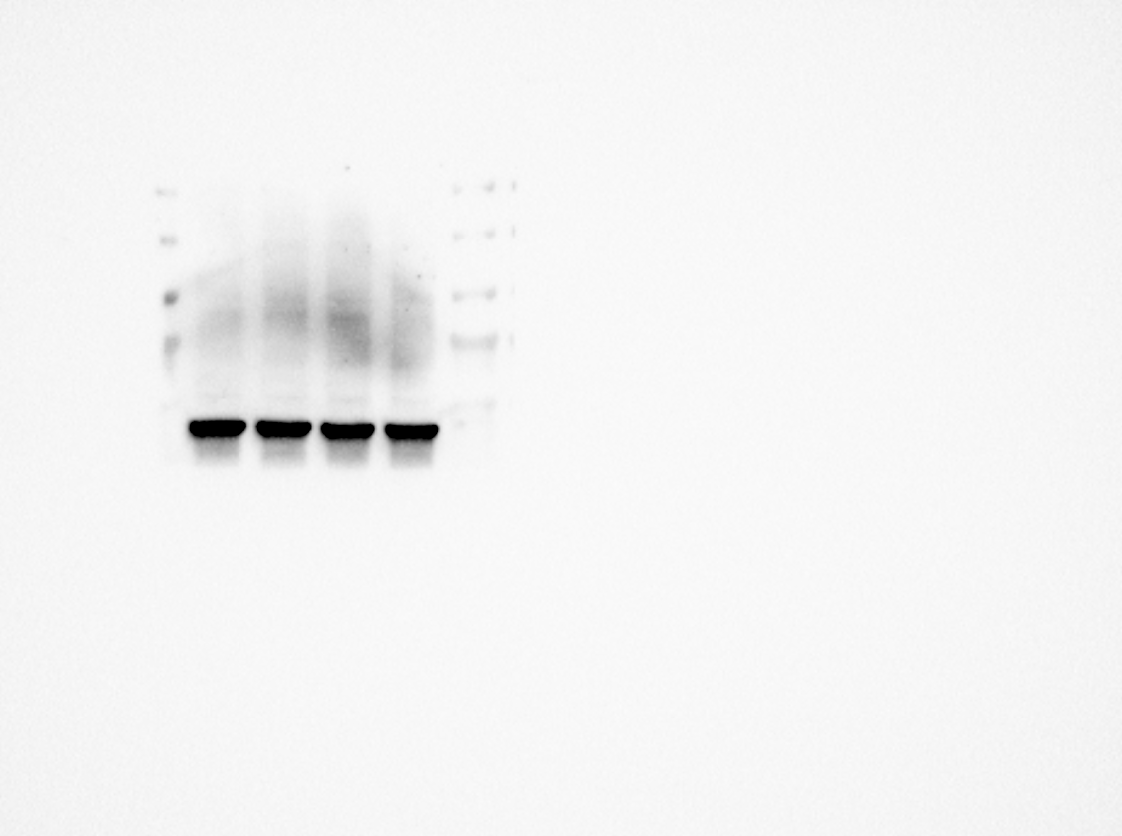

Supplement: Supplementary file 9 — Source Data File [file 41467_2023_43194_MOESM9_ESM.zip › Source Data File/Raw Data of Western blot/Supplementary Figure 2/replicate 1_display in Supplementary Figure2/eIF2alpha/loading control-alpha Tubulin/basal_T0_T1_T3.tif]

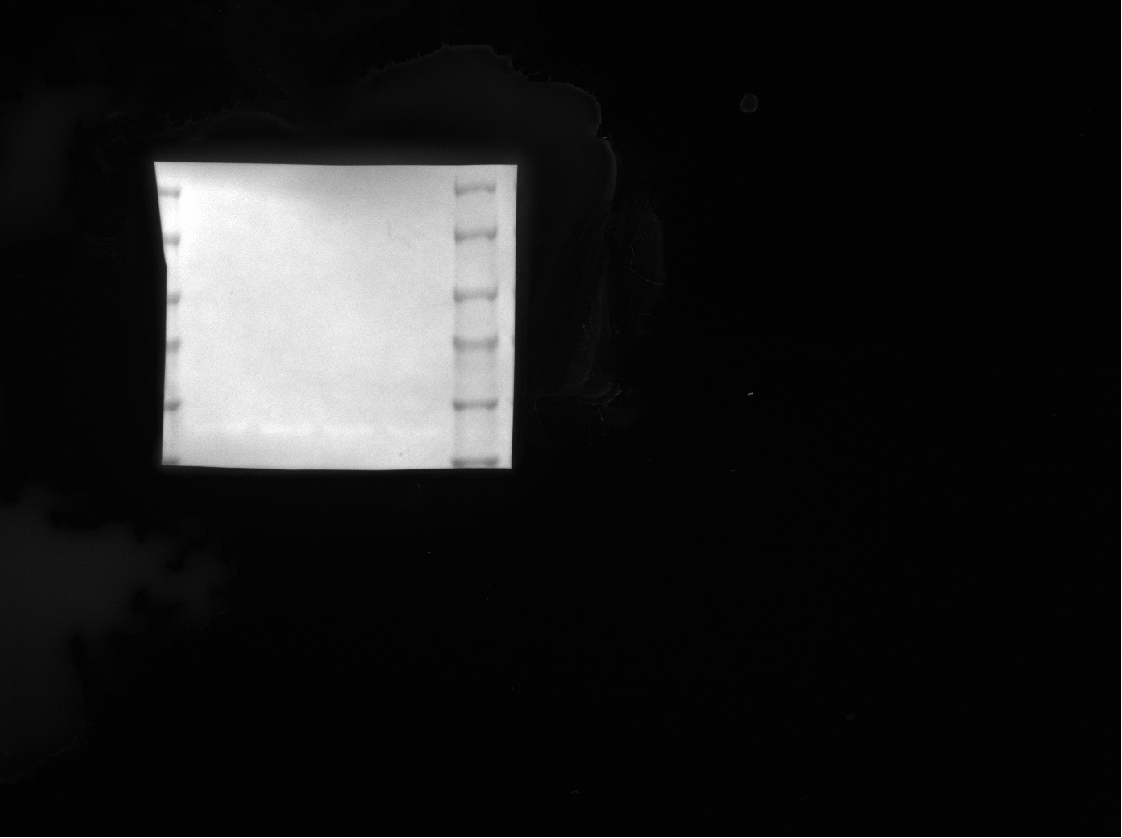

Supplement: Supplementary file 9 — Source Data File [file 41467_2023_43194_MOESM9_ESM.zip › Source Data File/Raw Data of Western blot/Supplementary Figure 2/replicate 1_display in Supplementary Figure2/eIF2alpha/loading control-alpha Tubulin/marker.tif]

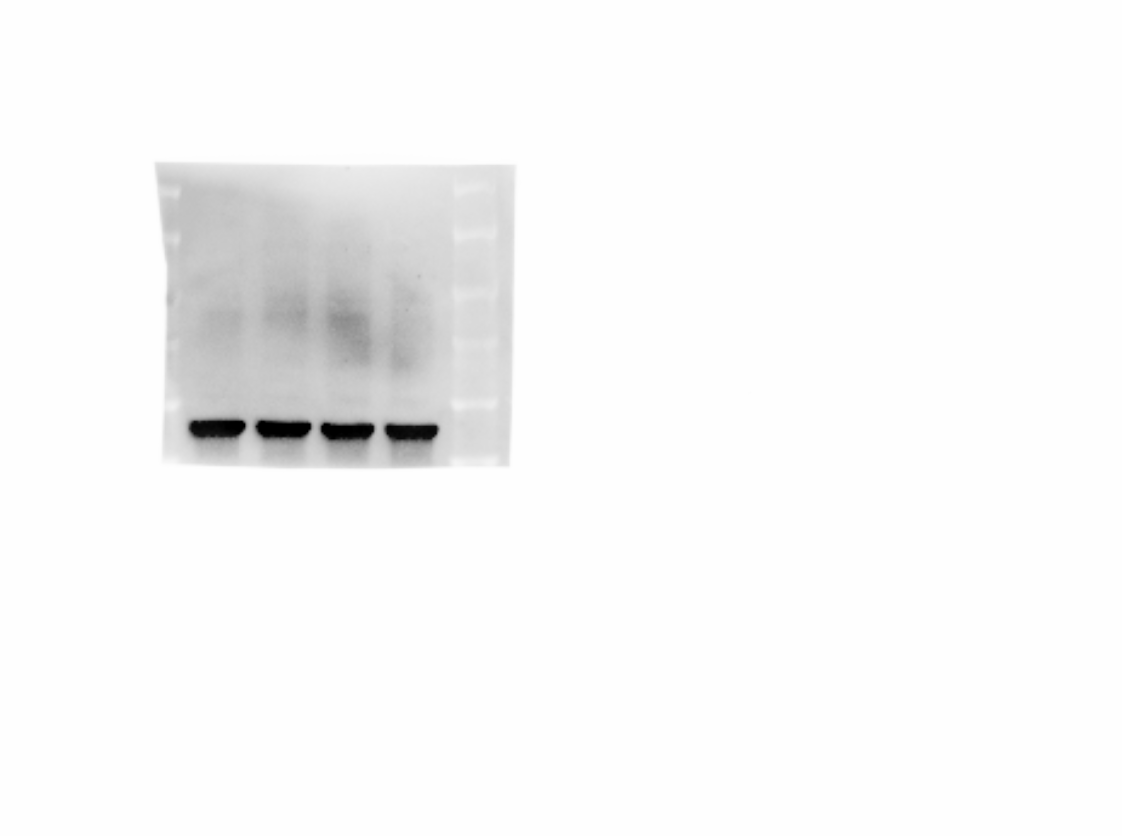

Supplement: Supplementary file 9 — Source Data File [file 41467_2023_43194_MOESM9_ESM.zip › Source Data File/Raw Data of Western blot/Supplementary Figure 2/replicate 1_display in Supplementary Figure2/eIF2alpha/loading control-alpha Tubulin/merge.tif]

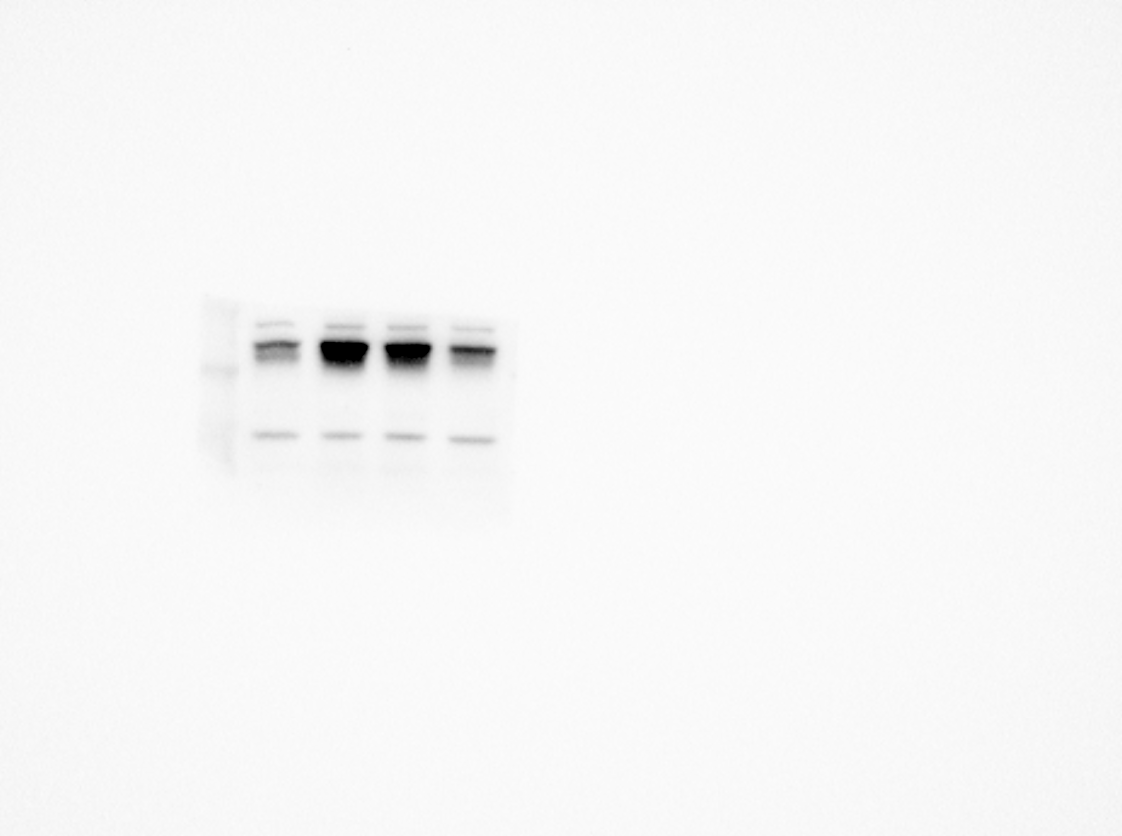

Supplement: Supplementary file 9 — Source Data File [file 41467_2023_43194_MOESM9_ESM.zip › Source Data File/Raw Data of Western blot/Supplementary Figure 2/replicate 2/eIF2alpha-phorspho/eIF2alpha-phorspho/basal_T0_T1_T3.tif]

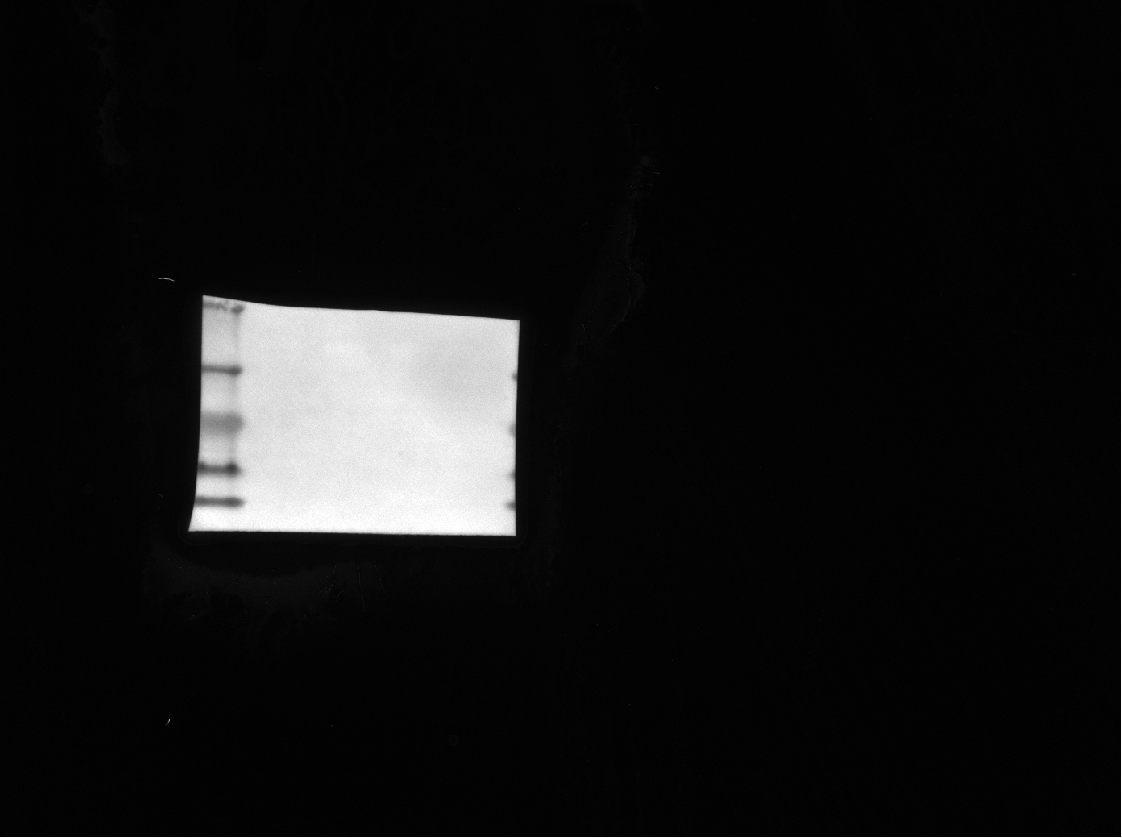

Supplement: Supplementary file 9 — Source Data File [file 41467_2023_43194_MOESM9_ESM.zip › Source Data File/Raw Data of Western blot/Supplementary Figure 2/replicate 2/eIF2alpha-phorspho/eIF2alpha-phorspho/marker.tif]

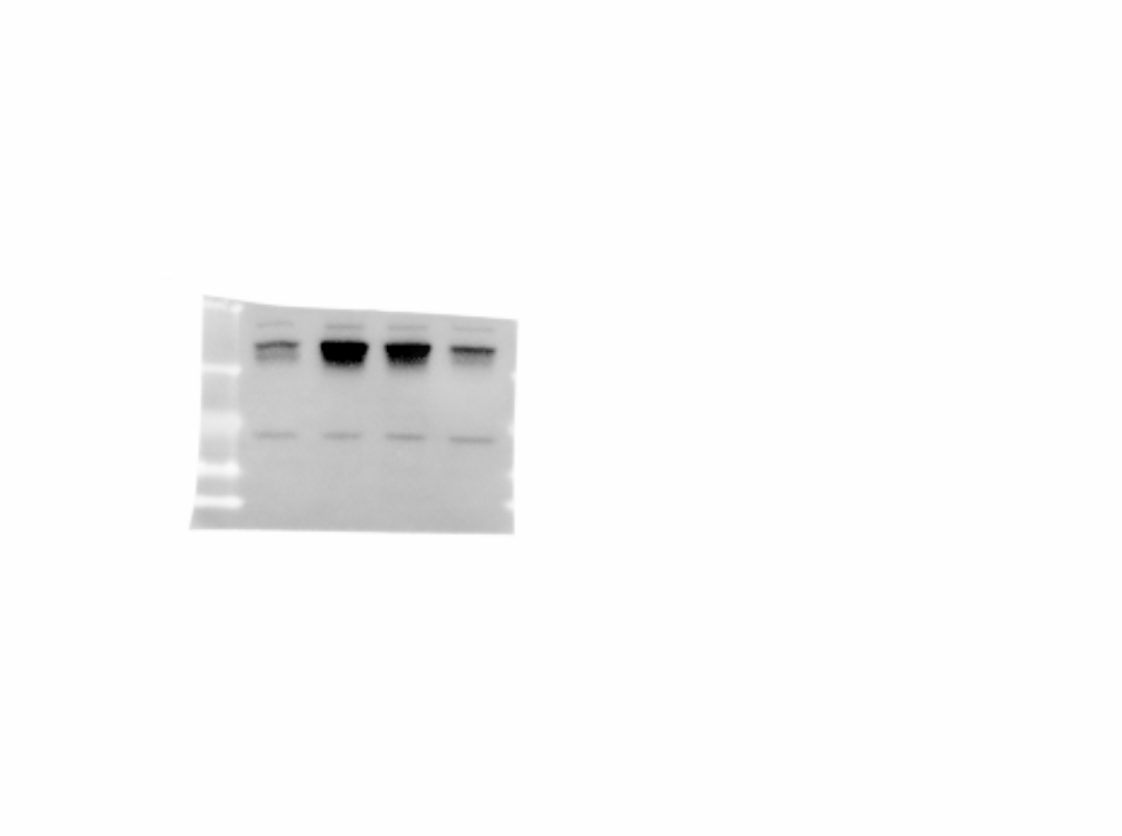

Supplement: Supplementary file 9 — Source Data File [file 41467_2023_43194_MOESM9_ESM.zip › Source Data File/Raw Data of Western blot/Supplementary Figure 2/replicate 2/eIF2alpha-phorspho/eIF2alpha-phorspho/merge.tif]

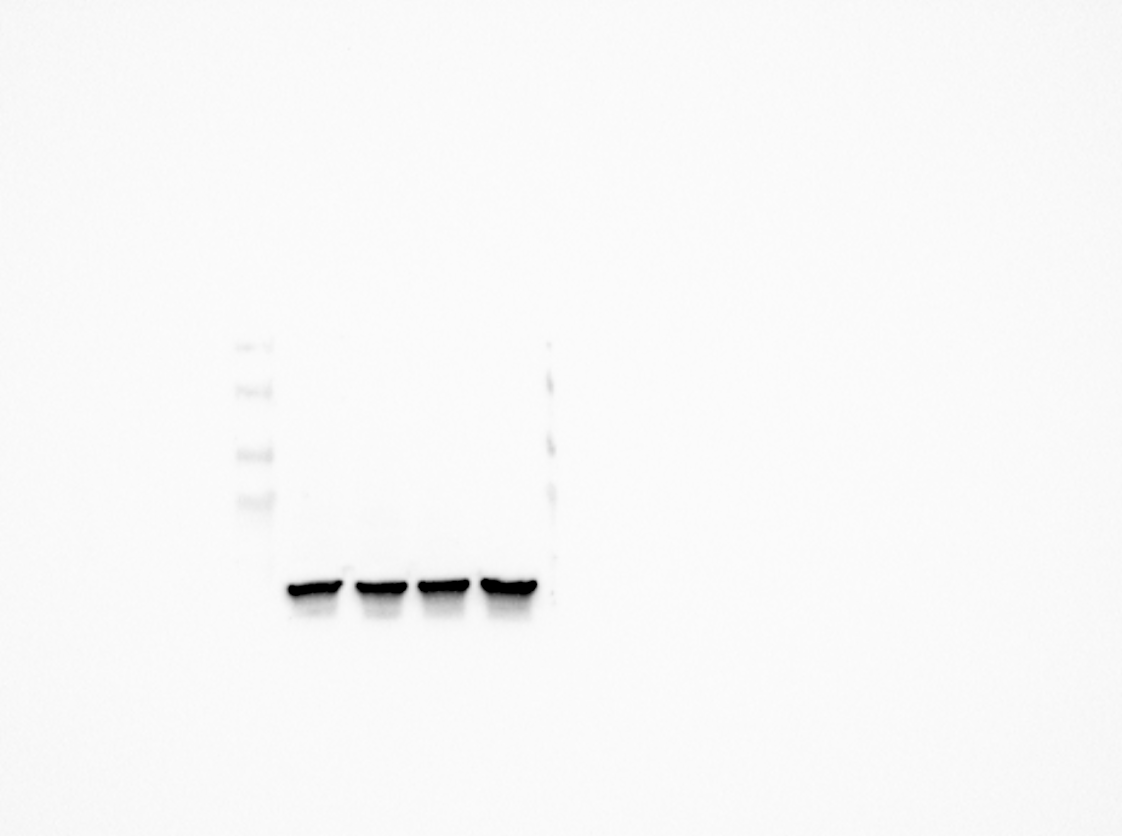

Supplement: Supplementary file 9 — Source Data File [file 41467_2023_43194_MOESM9_ESM.zip › Source Data File/Raw Data of Western blot/Supplementary Figure 2/replicate 2/eIF2alpha-phorspho/loading control-alpha Tubulin/basal_T0_T1_T3.tif]

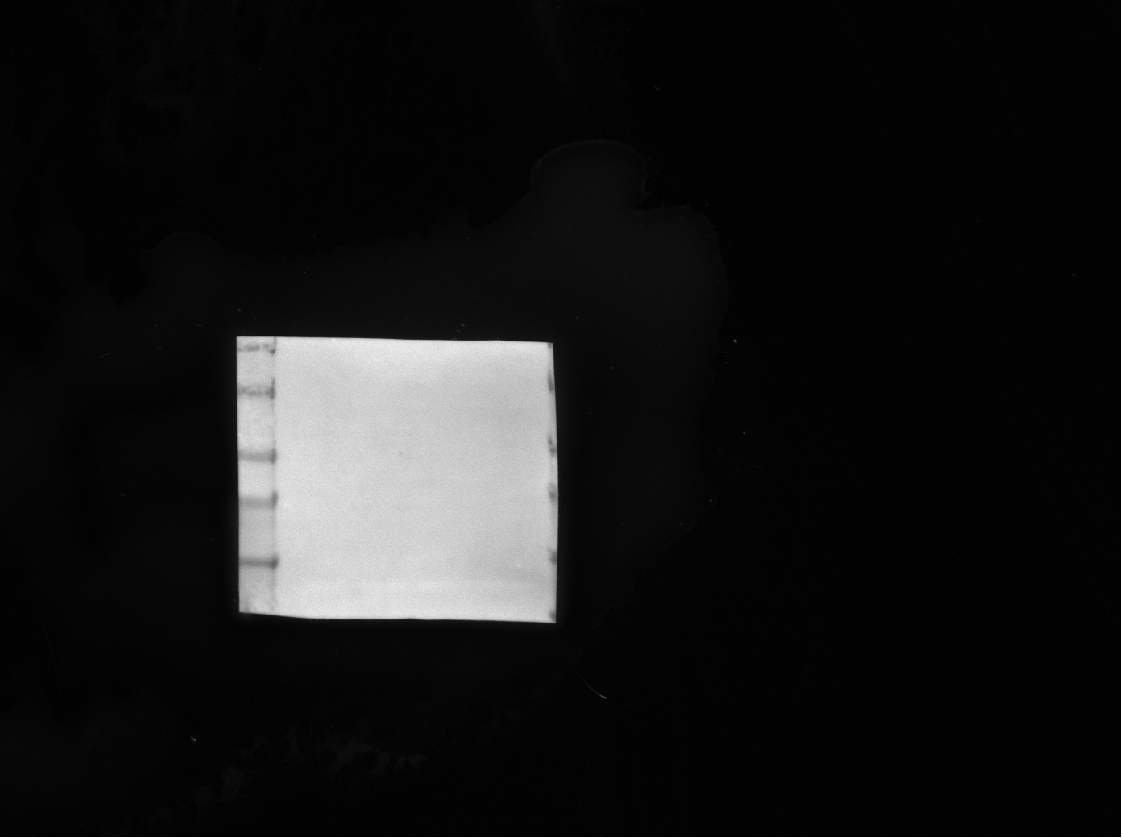

Supplement: Supplementary file 9 — Source Data File [file 41467_2023_43194_MOESM9_ESM.zip › Source Data File/Raw Data of Western blot/Supplementary Figure 2/replicate 2/eIF2alpha-phorspho/loading control-alpha Tubulin/marker.tif]

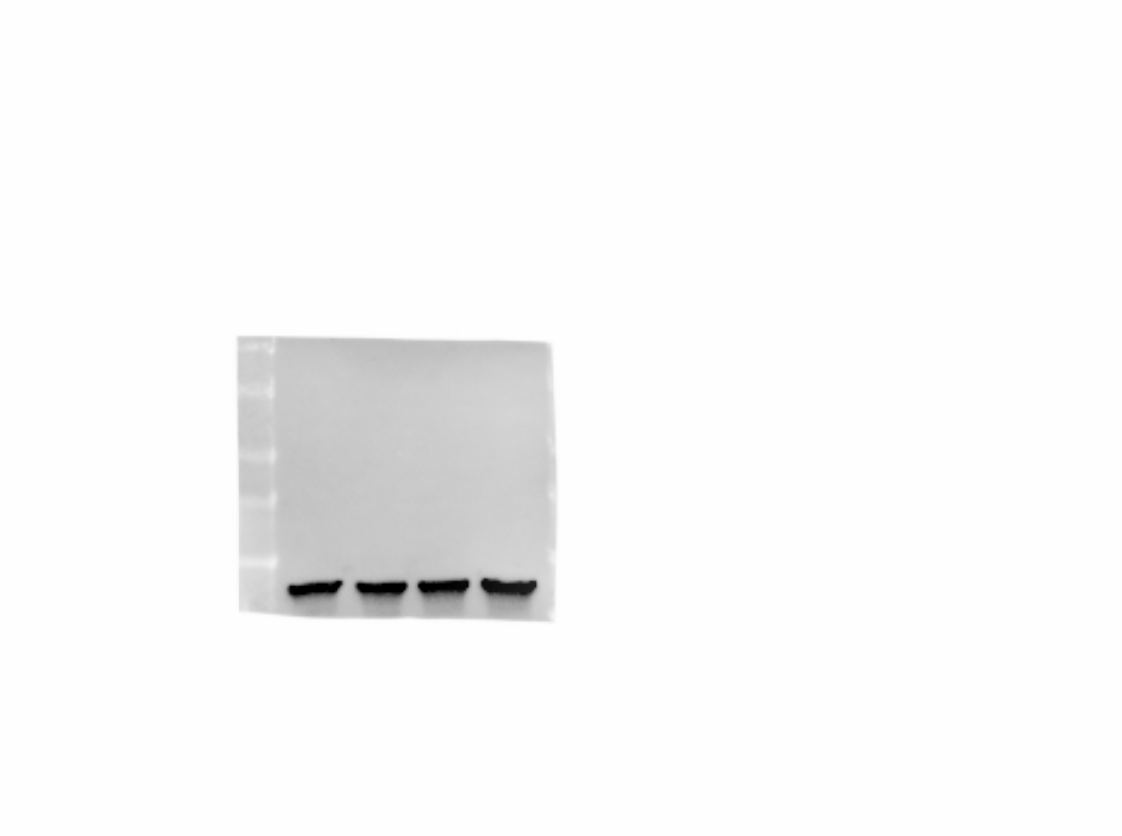

Supplement: Supplementary file 9 — Source Data File [file 41467_2023_43194_MOESM9_ESM.zip › Source Data File/Raw Data of Western blot/Supplementary Figure 2/replicate 2/eIF2alpha-phorspho/loading control-alpha Tubulin/merge.tif]

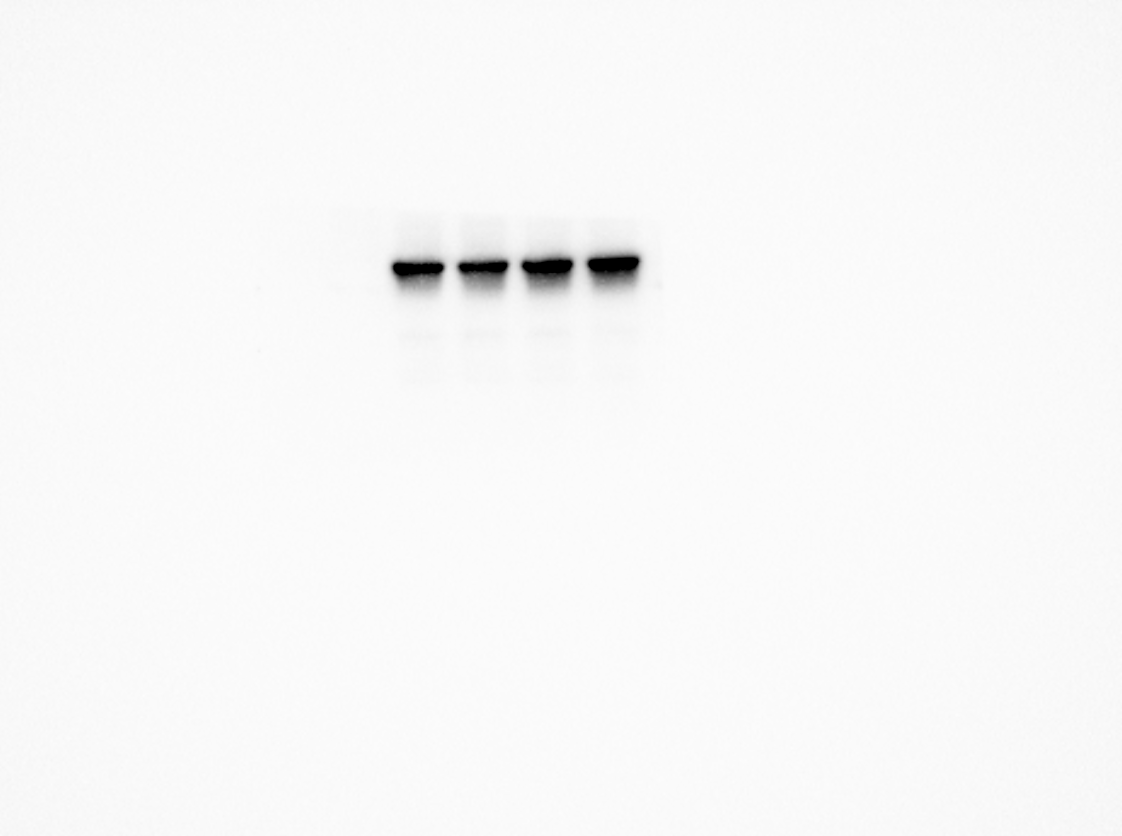

Supplement: Supplementary file 9 — Source Data File [file 41467_2023_43194_MOESM9_ESM.zip › Source Data File/Raw Data of Western blot/Supplementary Figure 2/replicate 2/eIF2alpha/eIF2alpha/basal_T0_T1_T3.tif]

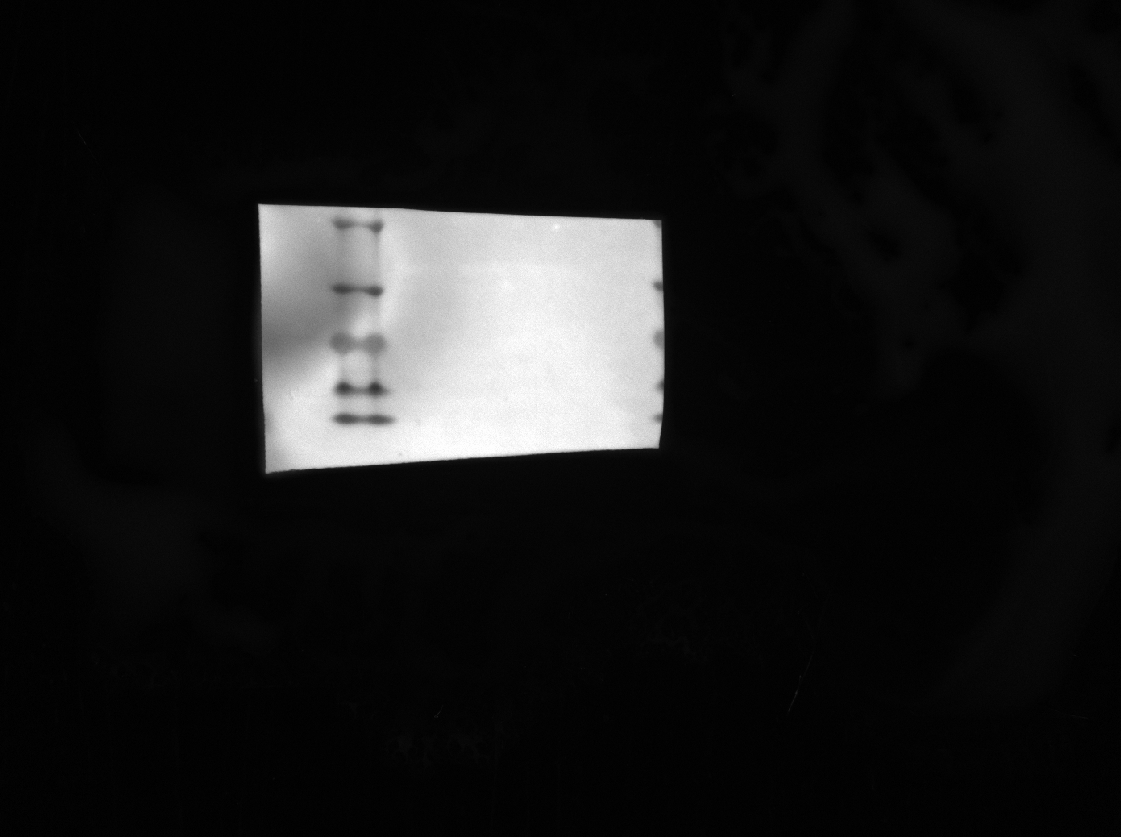

Supplement: Supplementary file 9 — Source Data File [file 41467_2023_43194_MOESM9_ESM.zip › Source Data File/Raw Data of Western blot/Supplementary Figure 2/replicate 2/eIF2alpha/eIF2alpha/marker.tif]

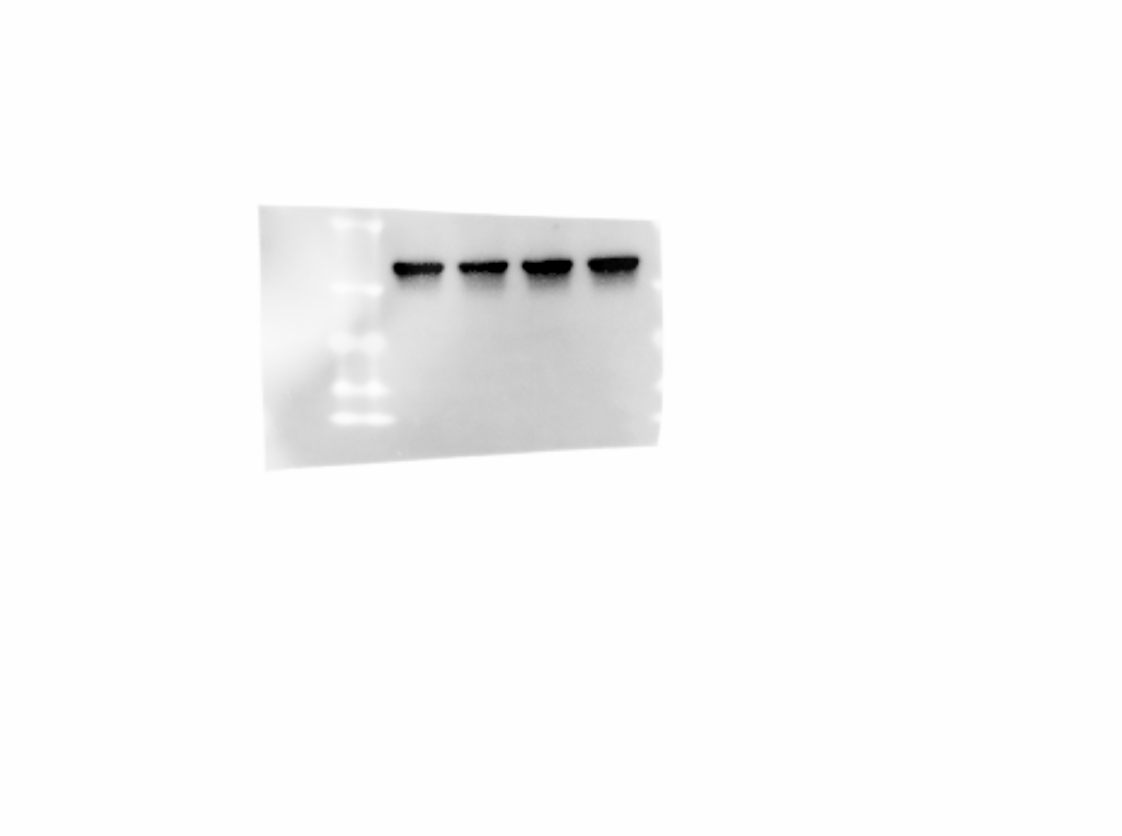

Supplement: Supplementary file 9 — Source Data File [file 41467_2023_43194_MOESM9_ESM.zip › Source Data File/Raw Data of Western blot/Supplementary Figure 2/replicate 2/eIF2alpha/eIF2alpha/merge.tif]

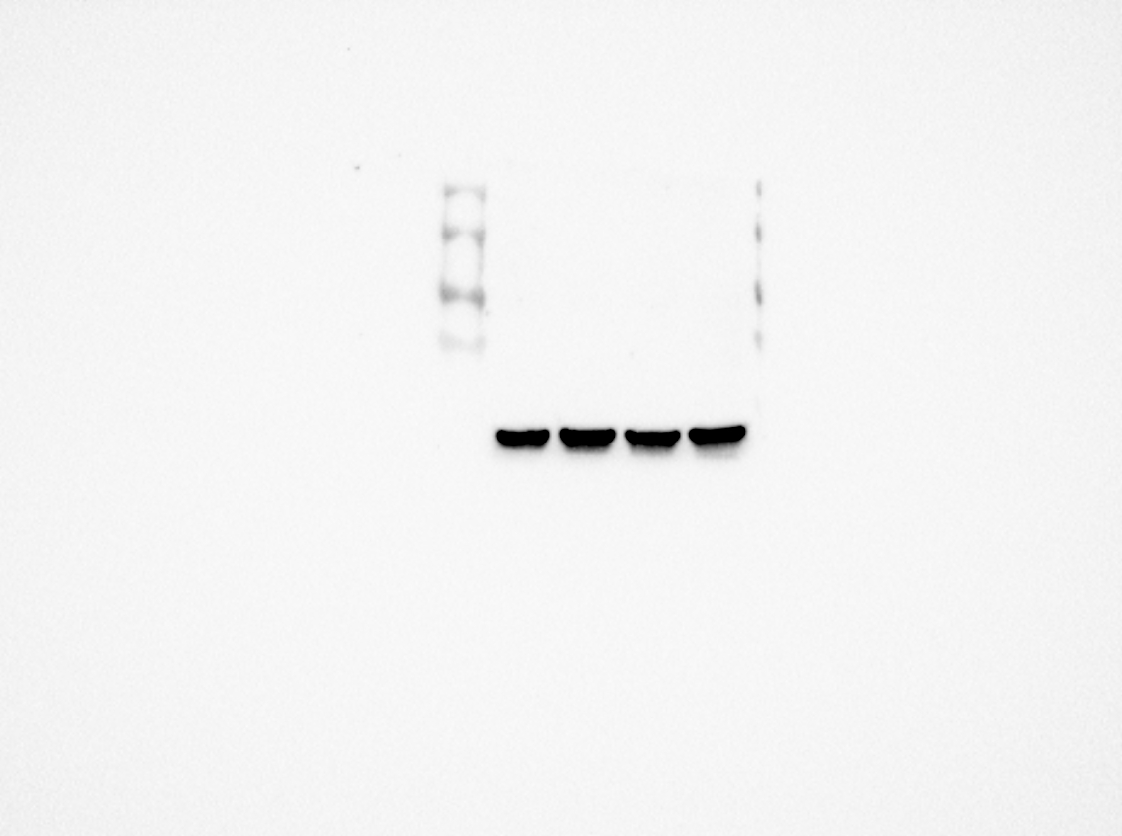

Supplement: Supplementary file 9 — Source Data File [file 41467_2023_43194_MOESM9_ESM.zip › Source Data File/Raw Data of Western blot/Supplementary Figure 2/replicate 2/eIF2alpha/loading control-alpha Tubulin/basal_T0_T1_T3.tif]

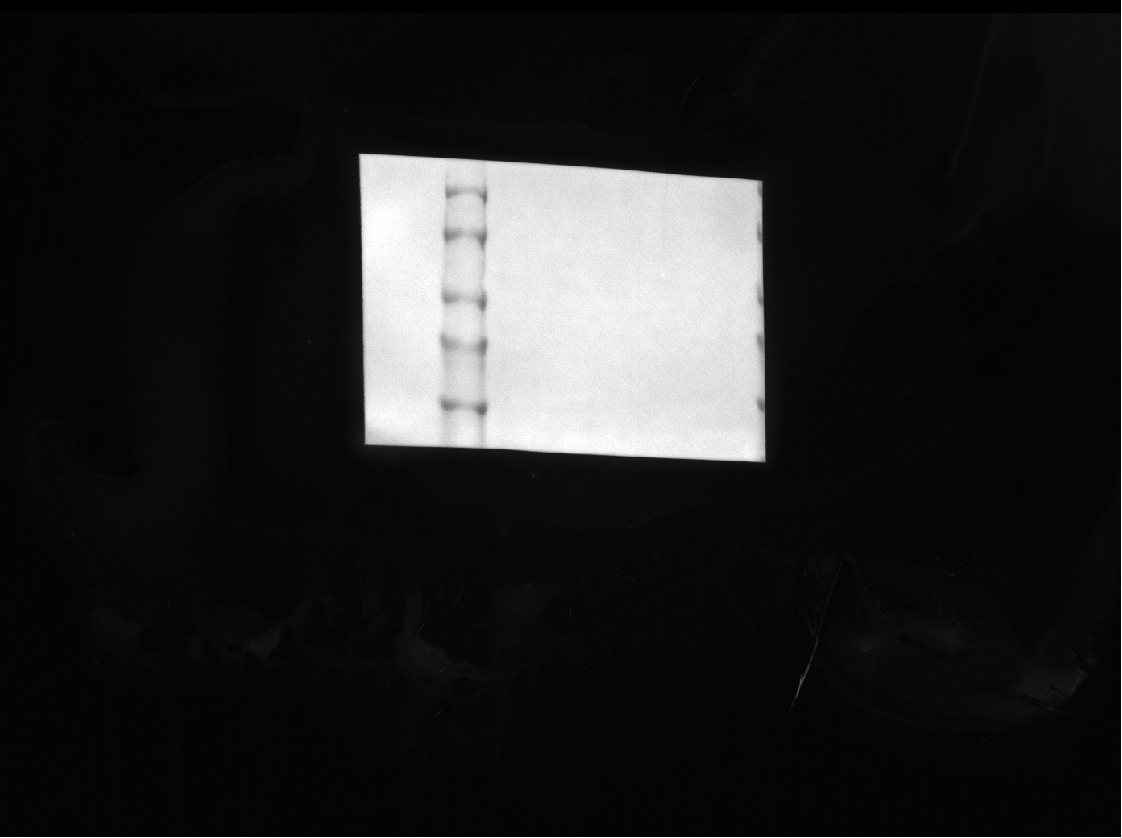

Supplement: Supplementary file 9 — Source Data File [file 41467_2023_43194_MOESM9_ESM.zip › Source Data File/Raw Data of Western blot/Supplementary Figure 2/replicate 2/eIF2alpha/loading control-alpha Tubulin/marker.tif]

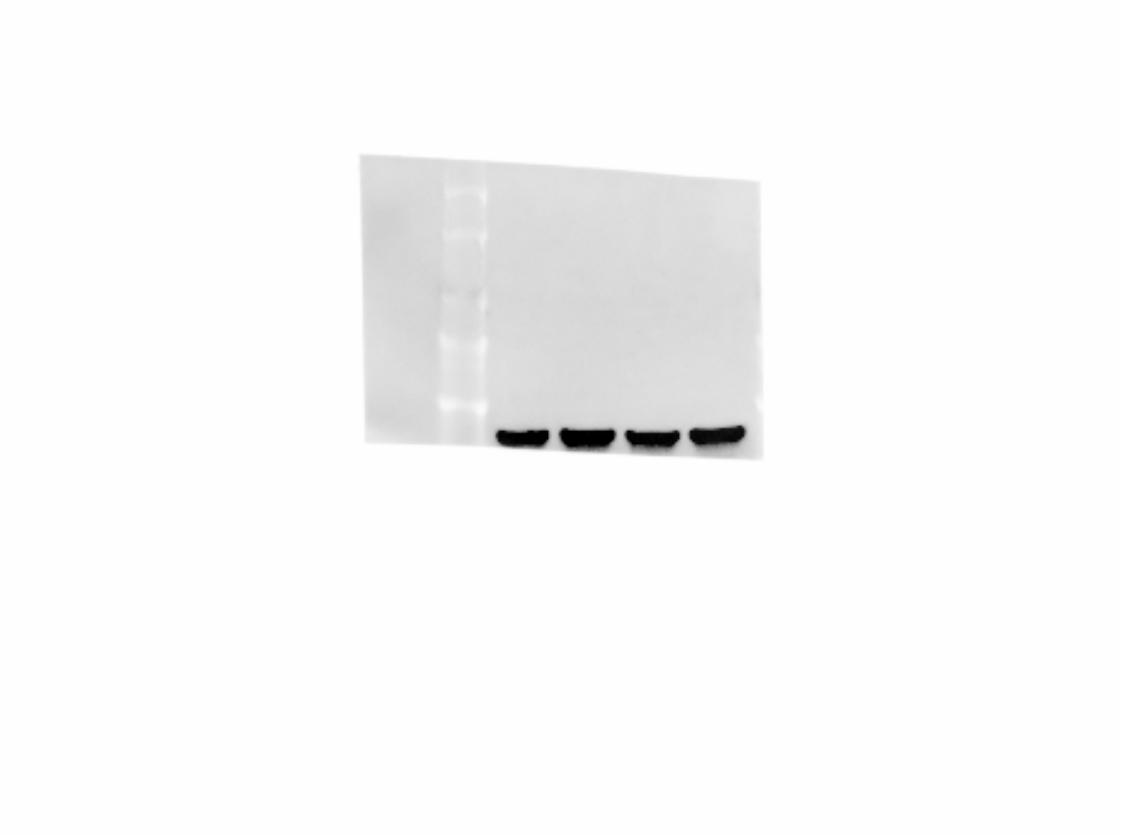

Supplement: Supplementary file 9 — Source Data File [file 41467_2023_43194_MOESM9_ESM.zip › Source Data File/Raw Data of Western blot/Supplementary Figure 2/replicate 2/eIF2alpha/loading control-alpha Tubulin/merge.tif]

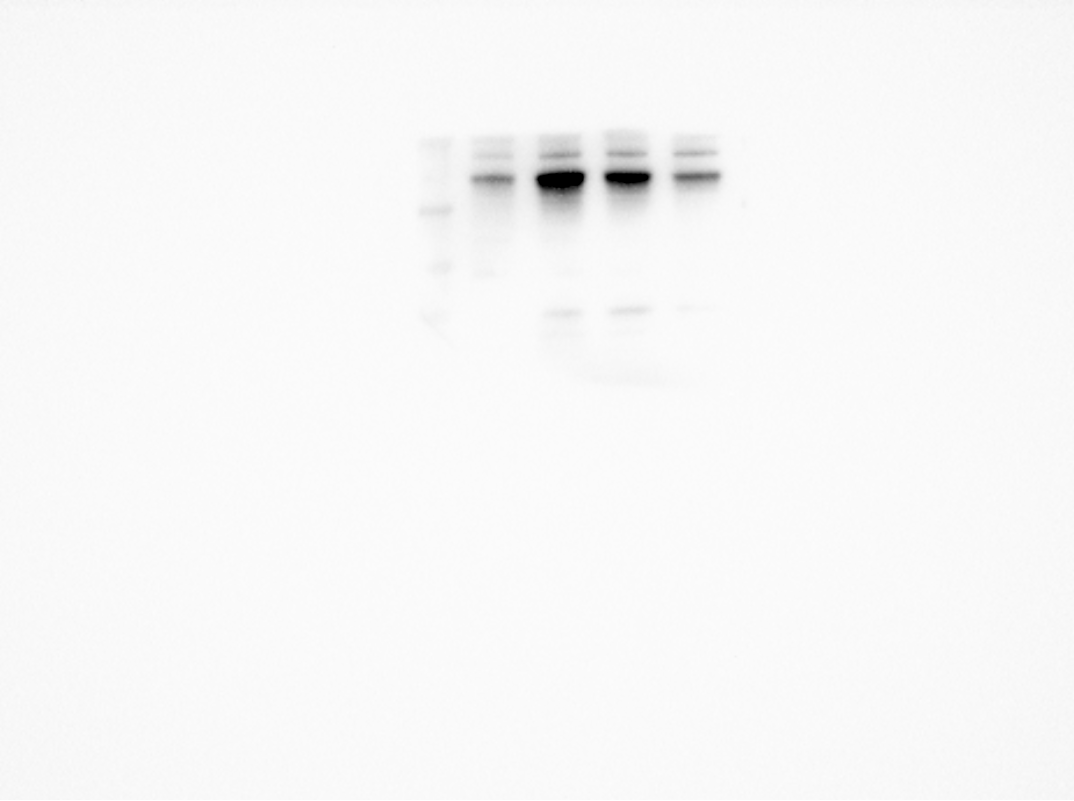

Supplement: Supplementary file 9 — Source Data File [file 41467_2023_43194_MOESM9_ESM.zip › Source Data File/Raw Data of Western blot/Supplementary Figure 2/replicate 3/eIF2alpha-phorspho/eIF2alpha-phorspho/basal_T0_T1_T3.tif]

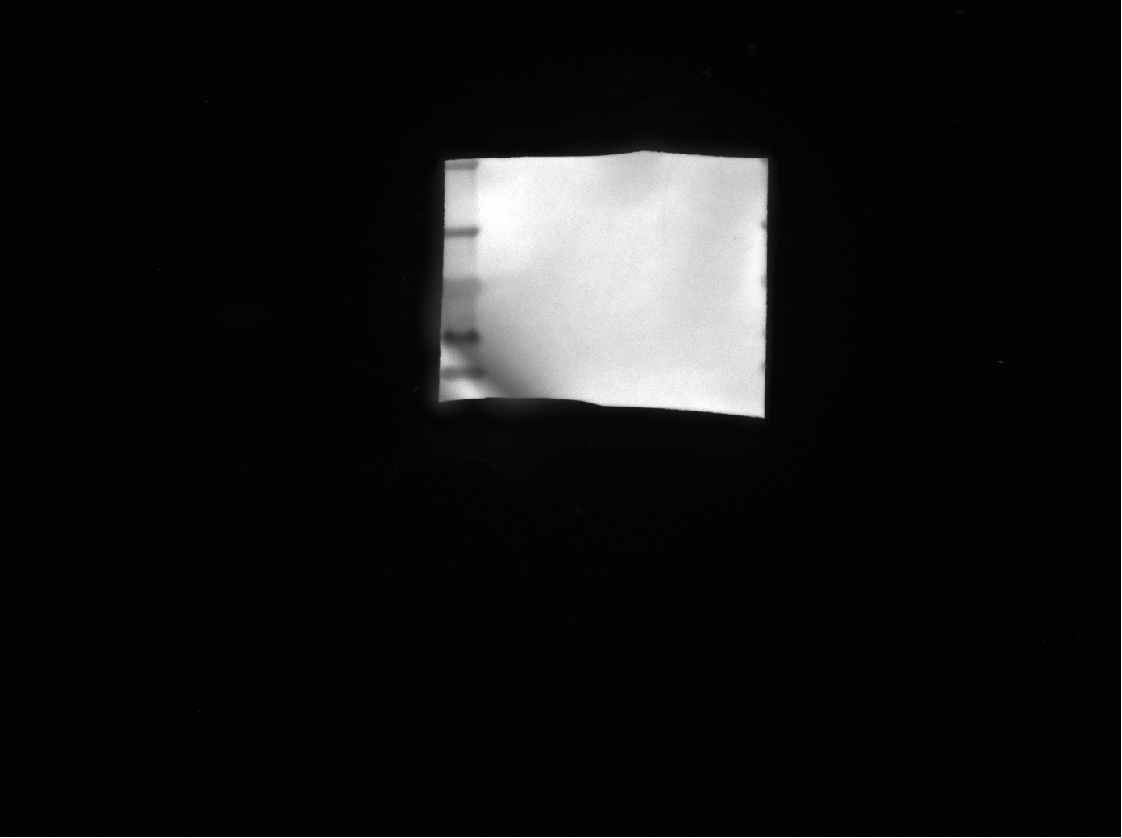

Supplement: Supplementary file 9 — Source Data File [file 41467_2023_43194_MOESM9_ESM.zip › Source Data File/Raw Data of Western blot/Supplementary Figure 2/replicate 3/eIF2alpha-phorspho/eIF2alpha-phorspho/marker.tif]

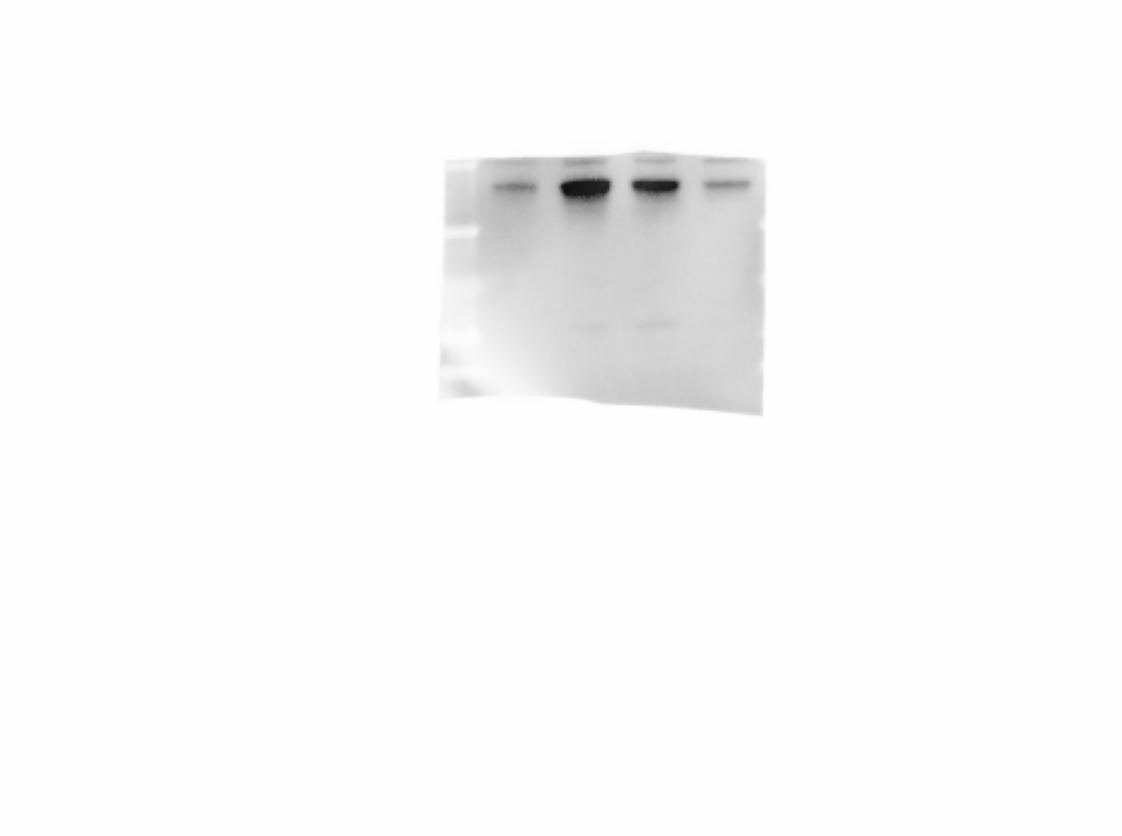

Supplement: Supplementary file 9 — Source Data File [file 41467_2023_43194_MOESM9_ESM.zip › Source Data File/Raw Data of Western blot/Supplementary Figure 2/replicate 3/eIF2alpha-phorspho/eIF2alpha-phorspho/merge.tif]

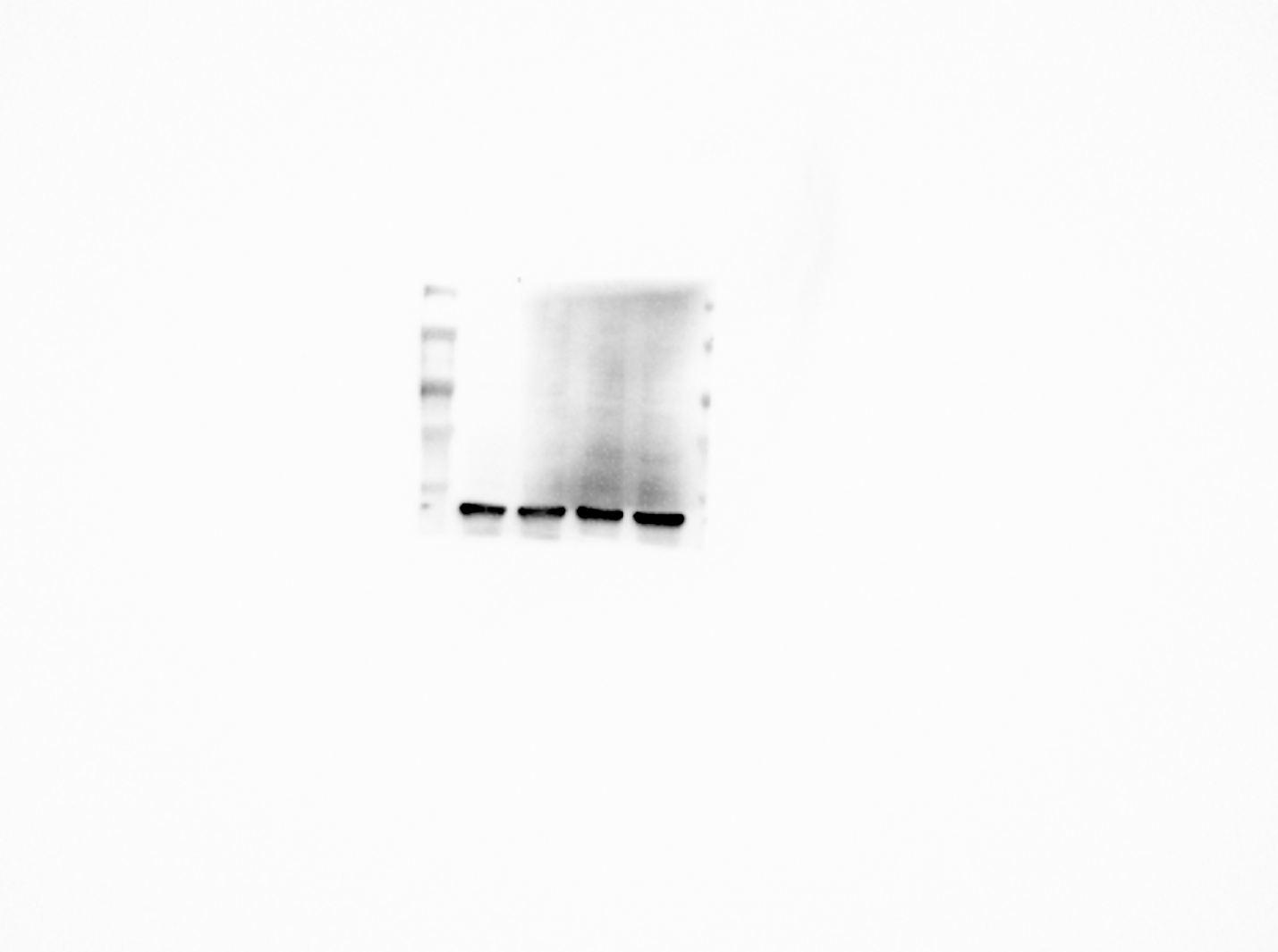

Supplement: Supplementary file 9 — Source Data File [file 41467_2023_43194_MOESM9_ESM.zip › Source Data File/Raw Data of Western blot/Supplementary Figure 2/replicate 3/eIF2alpha-phorspho/loading control-alpha Tubulin/basal_T0_T1_T3.tif]

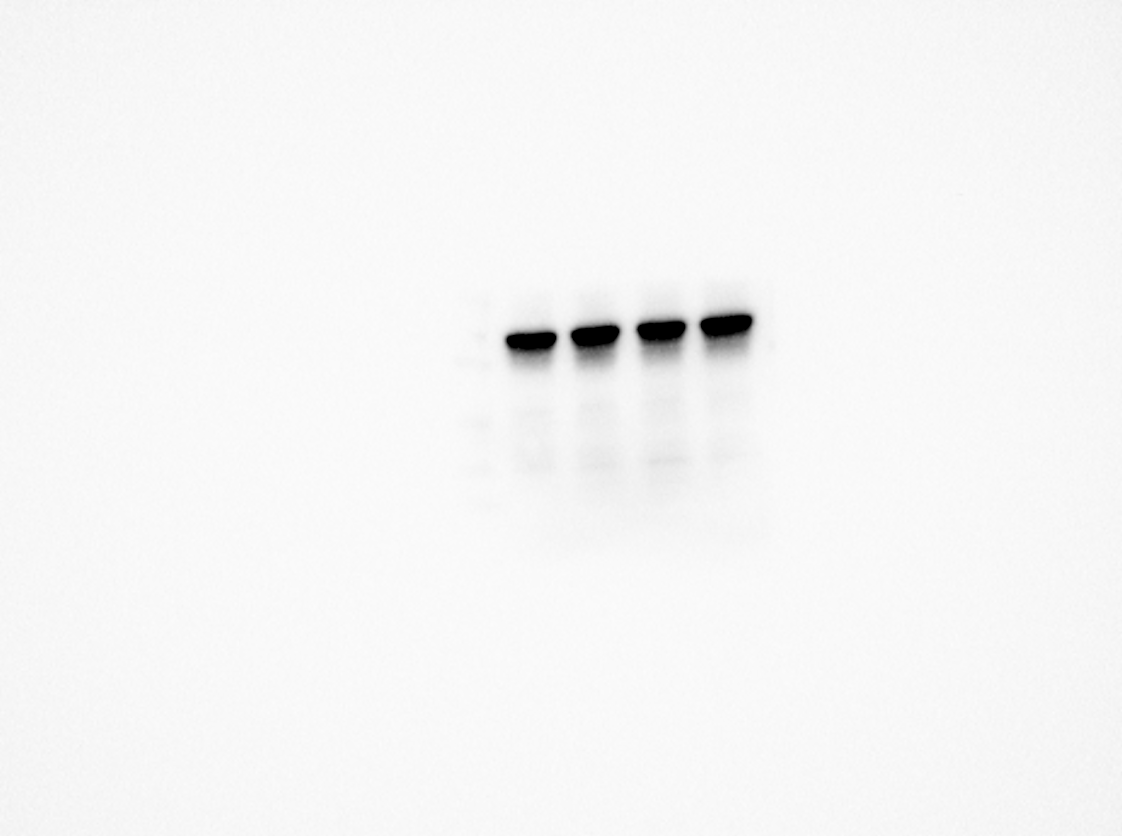

Supplement: Supplementary file 9 — Source Data File [file 41467_2023_43194_MOESM9_ESM.zip › Source Data File/Raw Data of Western blot/Supplementary Figure 2/replicate 3/eIF2alpha/eIF2alpha/basal_T0_T1_T3.tif]

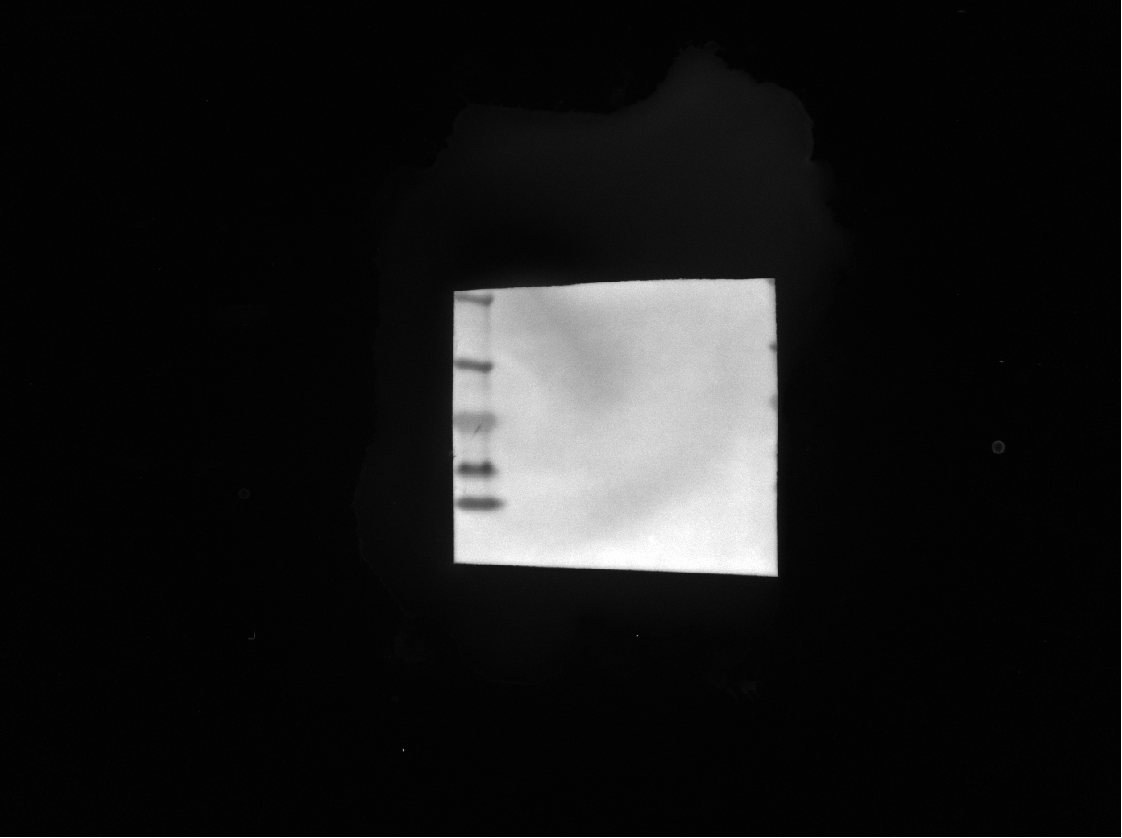

Supplement: Supplementary file 9 — Source Data File [file 41467_2023_43194_MOESM9_ESM.zip › Source Data File/Raw Data of Western blot/Supplementary Figure 2/replicate 3/eIF2alpha/eIF2alpha/marker.tif]

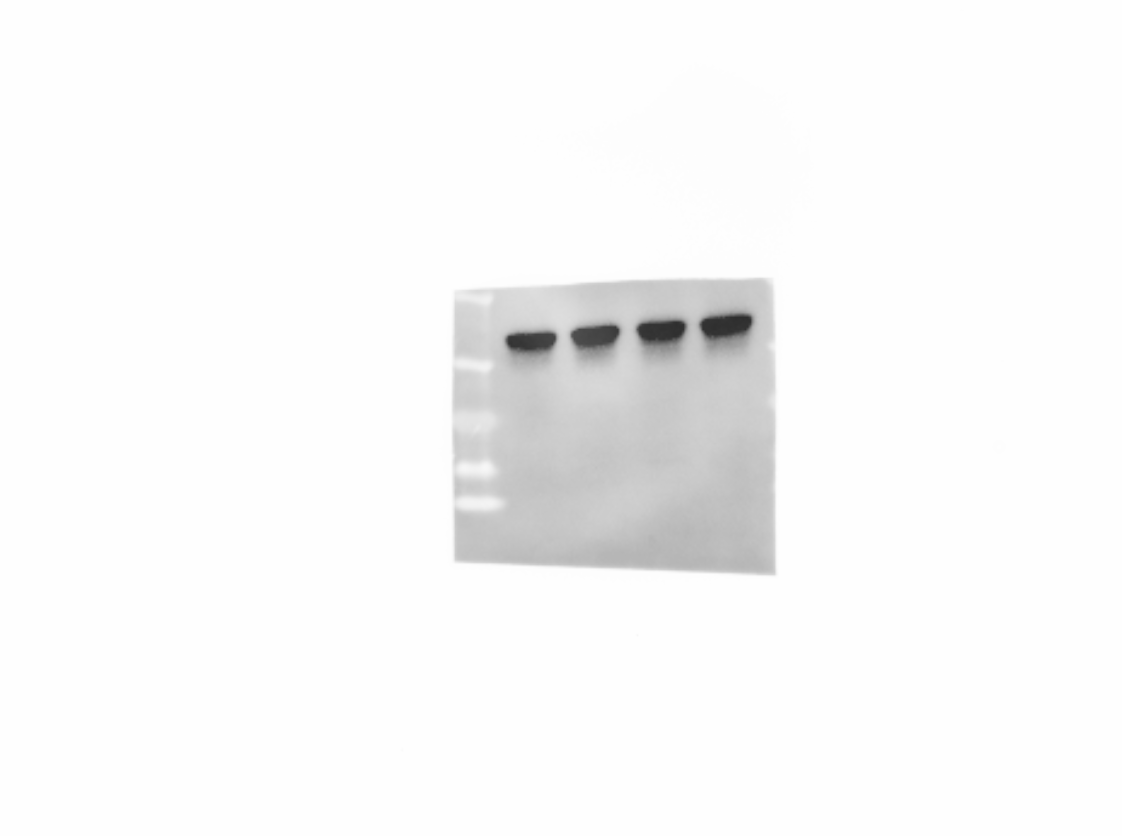

Supplement: Supplementary file 9 — Source Data File [file 41467_2023_43194_MOESM9_ESM.zip › Source Data File/Raw Data of Western blot/Supplementary Figure 2/replicate 3/eIF2alpha/eIF2alpha/merge.tif]

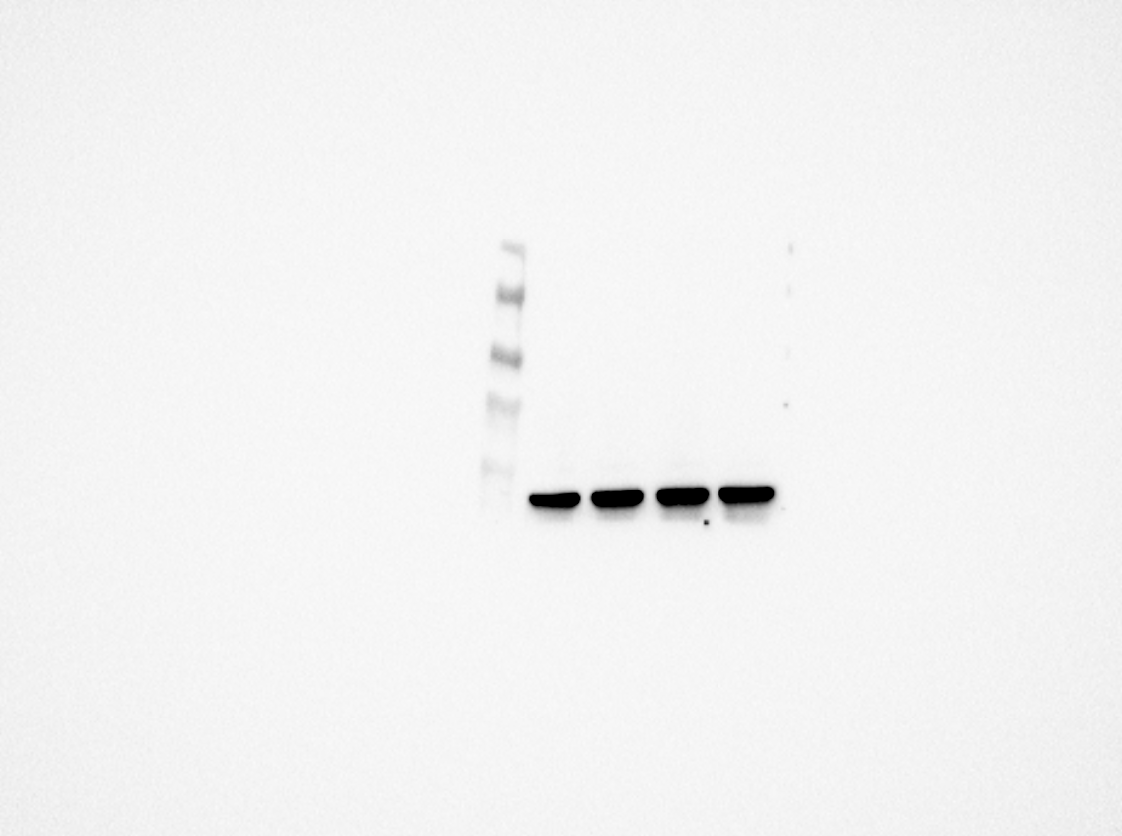

Supplement: Supplementary file 9 — Source Data File [file 41467_2023_43194_MOESM9_ESM.zip › Source Data File/Raw Data of Western blot/Supplementary Figure 2/replicate 3/eIF2alpha/loading control-alpha Tubulin/basal_T0_T1_T3.tif]

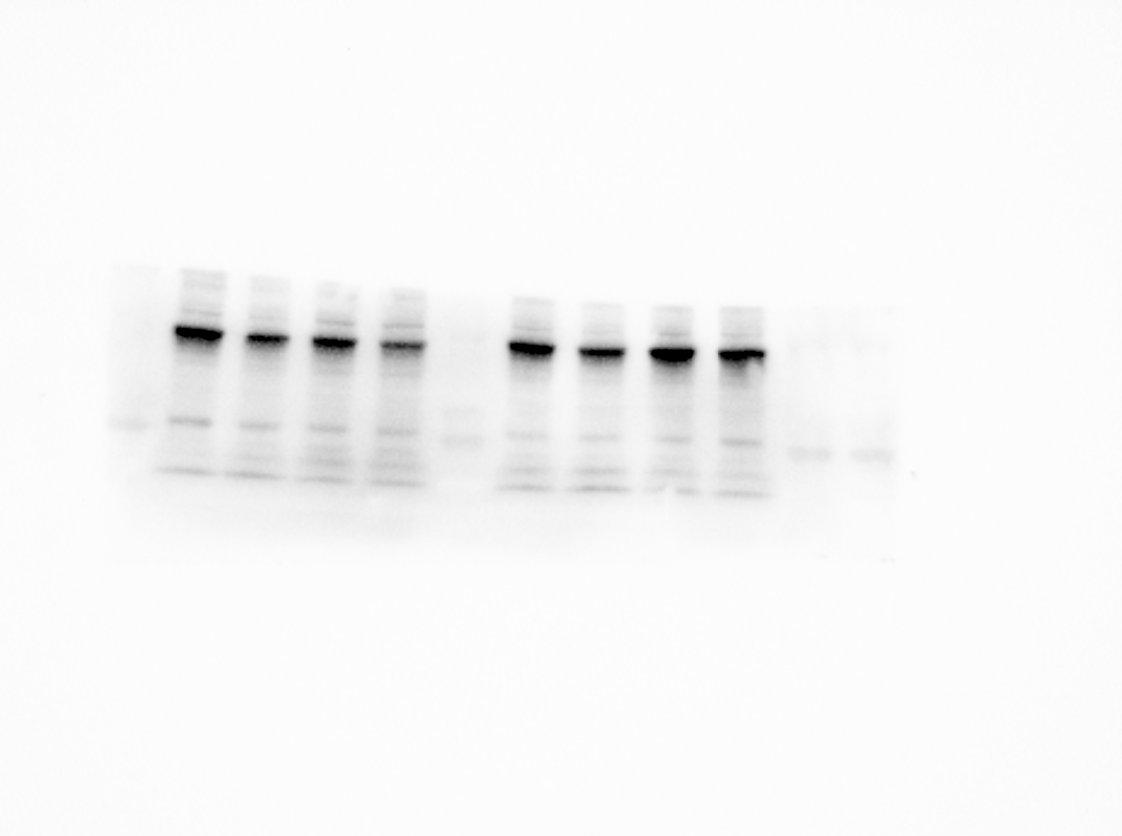

Supplement: Supplementary file 9 — Source Data File [file 41467_2023_43194_MOESM9_ESM.zip › Source Data File/Raw Data of Western blot/Supplementary Figure 5/replicate 1-display in Supplementary Figure 5/eIF2alpha-phorspho/eIF2alpha-phorspho/four lane on the left side_label Noprobe Nolight blank.tif]

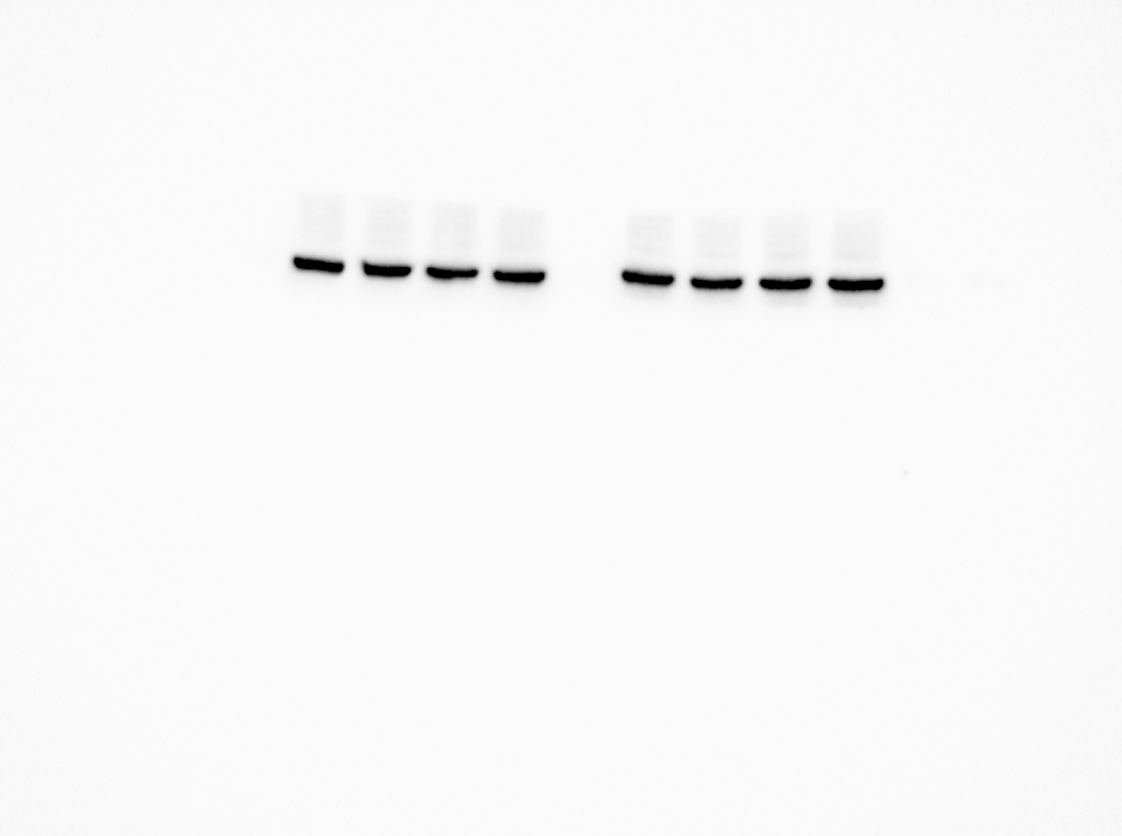

Supplement: Supplementary file 9 — Source Data File [file 41467_2023_43194_MOESM9_ESM.zip › Source Data File/Raw Data of Western blot/Supplementary Figure 5/replicate 1-display in Supplementary Figure 5/eIF2alpha-phorspho/loading control-alpha Tubulin/four lane on the left side_label Noprobe Nolight blank.tif]

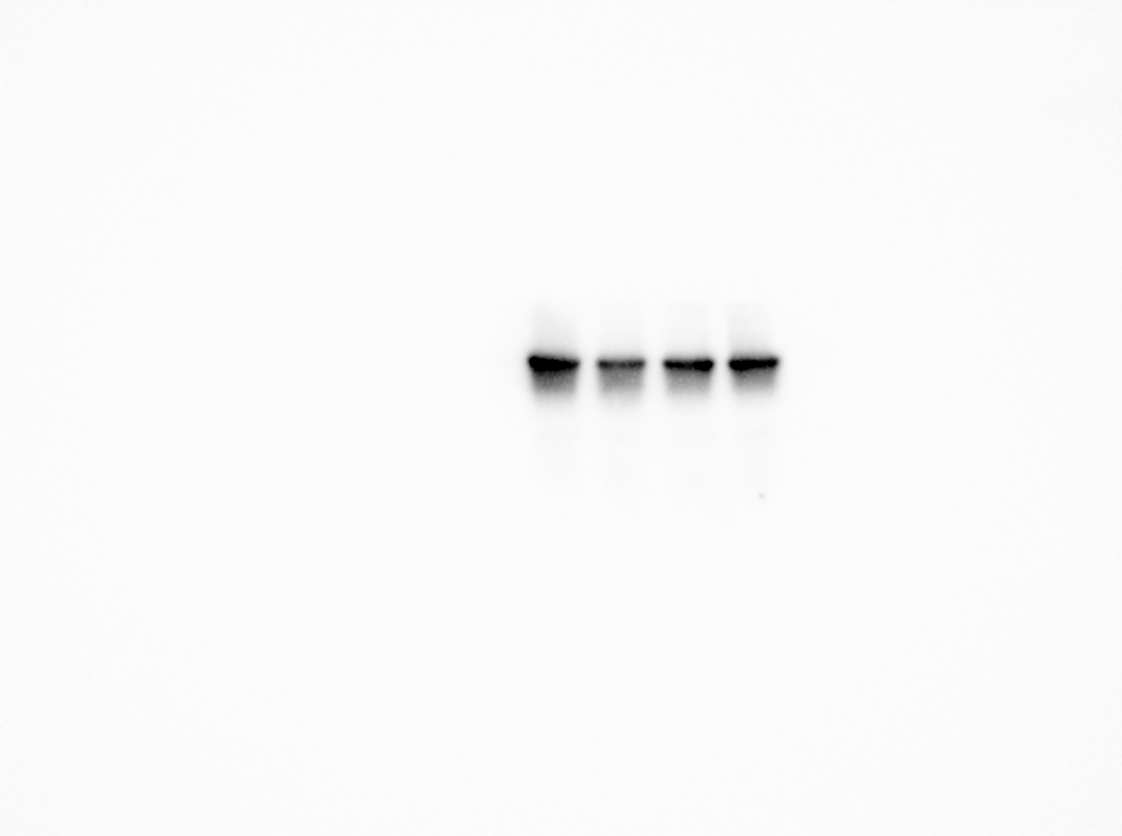

Supplement: Supplementary file 9 — Source Data File [file 41467_2023_43194_MOESM9_ESM.zip › Source Data File/Raw Data of Western blot/Supplementary Figure 5/replicate 1-display in Supplementary Figure 5/eIF2alpha/eIF2alpha/label_Noprobe_Nolight_blank.tif]

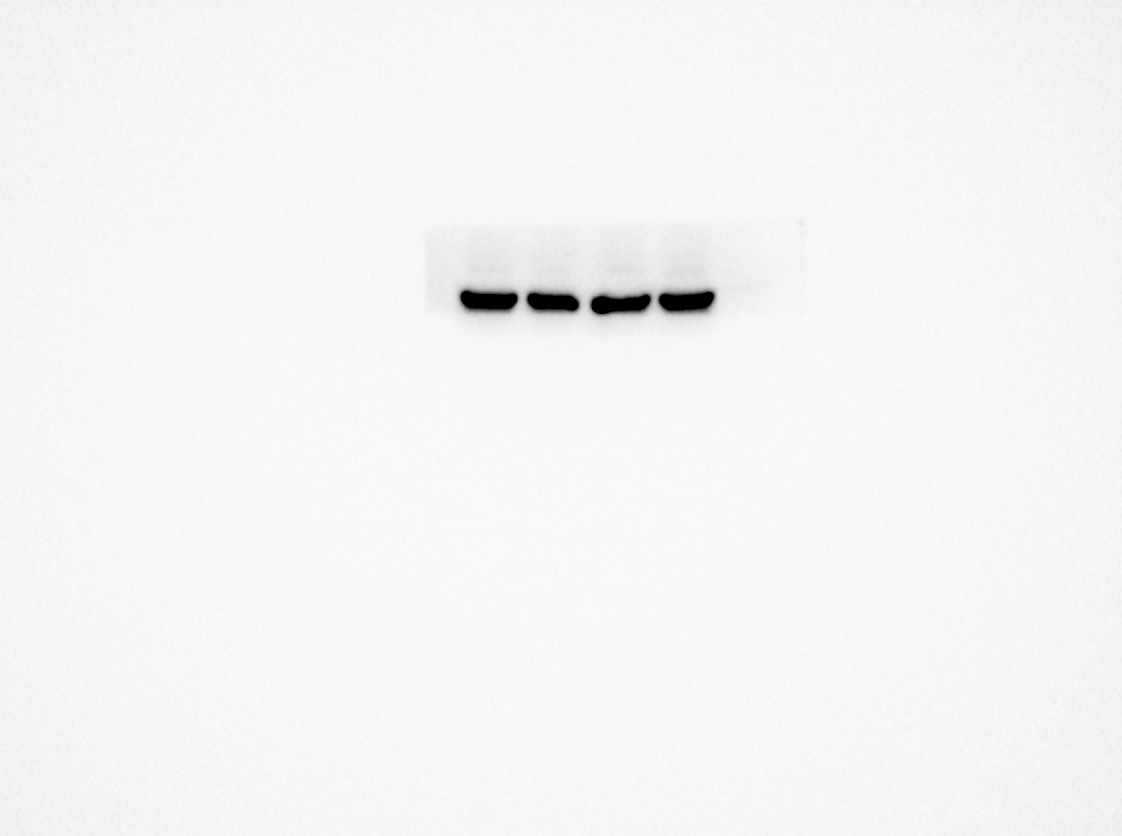

Supplement: Supplementary file 9 — Source Data File [file 41467_2023_43194_MOESM9_ESM.zip › Source Data File/Raw Data of Western blot/Supplementary Figure 5/replicate 1-display in Supplementary Figure 5/eIF2alpha/loading control-alpha Tubulin/label_noprobe_nolight_blank.tif]

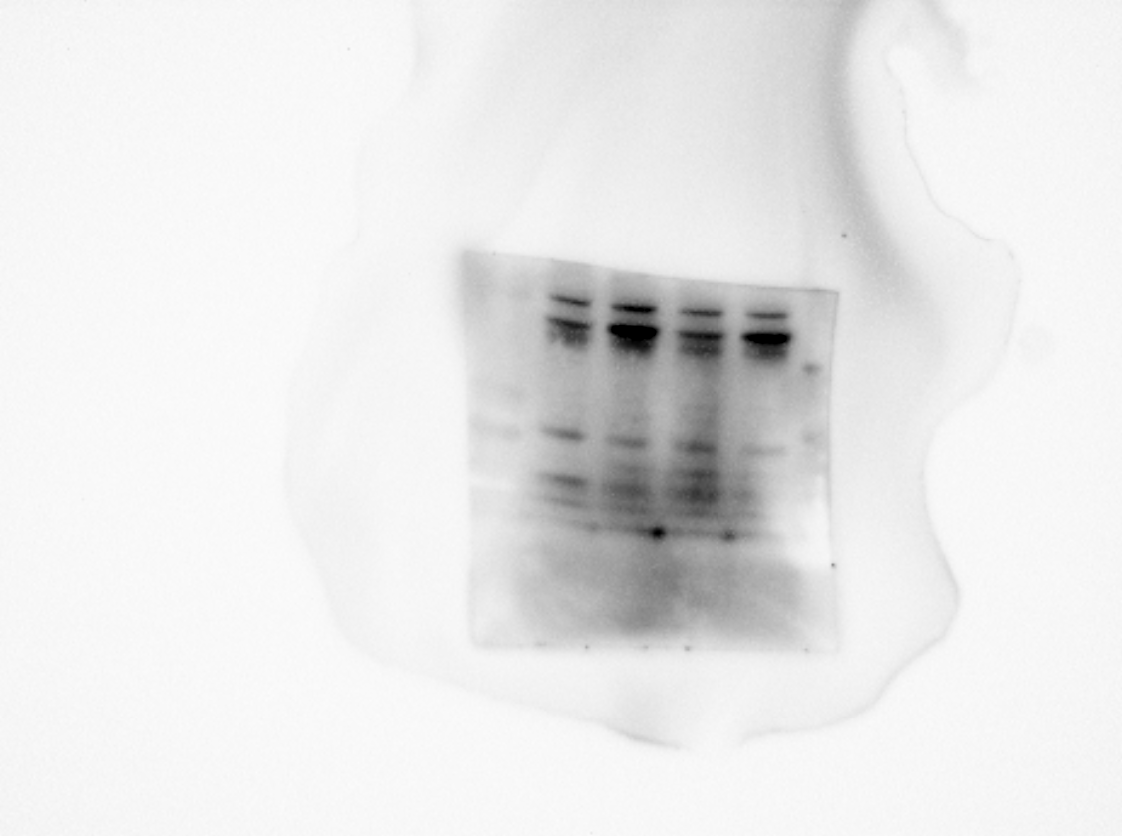

Supplement: Supplementary file 9 — Source Data File [file 41467_2023_43194_MOESM9_ESM.zip › Source Data File/Raw Data of Western blot/Supplementary Figure 5/replicate 2/eIF2alpha-phorspho/eIF2alpha-phorspho/blank_label_noPA_nolight.tif]

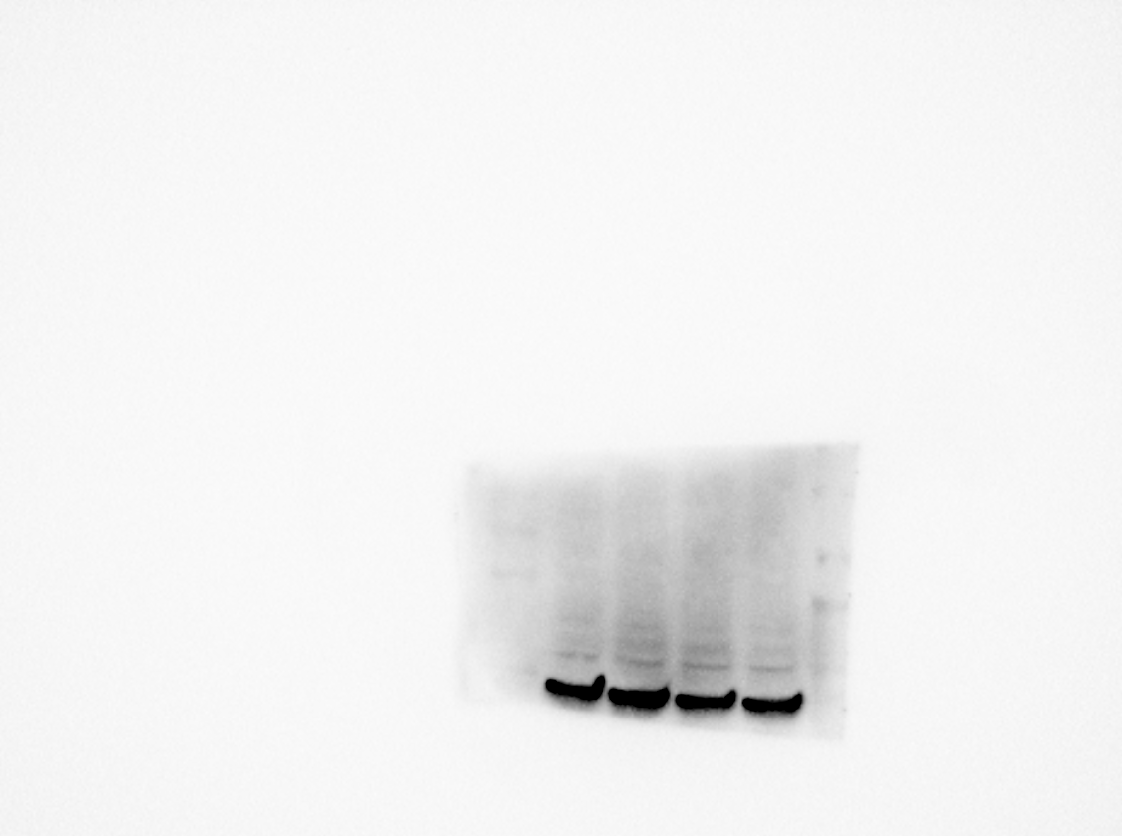

Supplement: Supplementary file 9 — Source Data File [file 41467_2023_43194_MOESM9_ESM.zip › Source Data File/Raw Data of Western blot/Supplementary Figure 5/replicate 2/eIF2alpha-phorspho/loading control-alpha Tubulin/blank_label_noPA_nolight.tif]

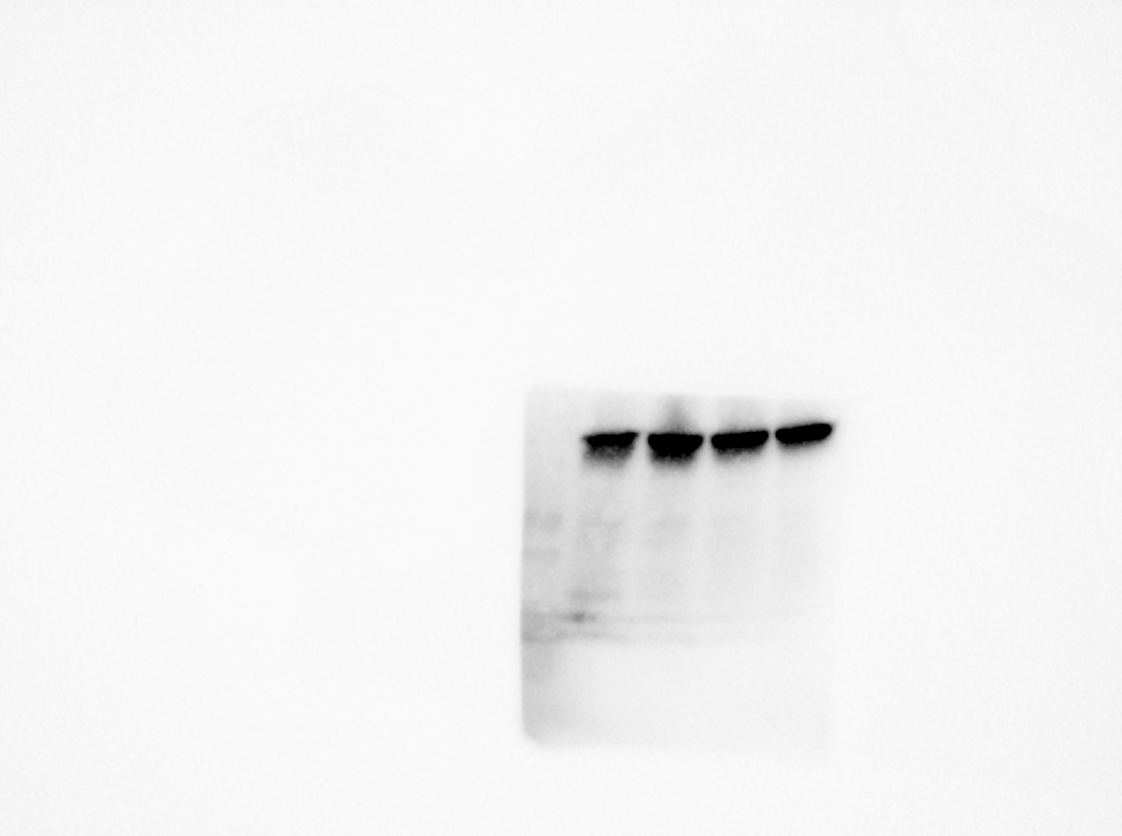

Supplement: Supplementary file 9 — Source Data File [file 41467_2023_43194_MOESM9_ESM.zip › Source Data File/Raw Data of Western blot/Supplementary Figure 5/replicate 2/eIF2alpha/eIF2alpha/blank_label_noPA_nolight.tif]

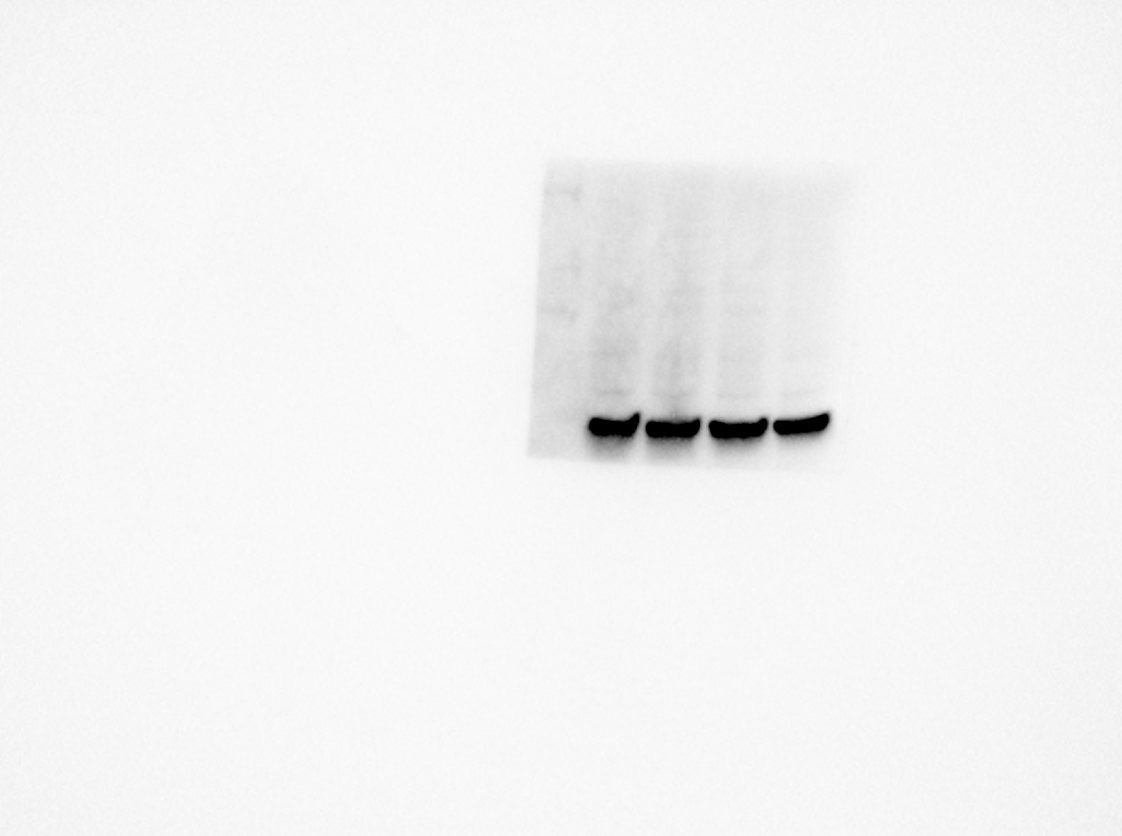

Supplement: Supplementary file 9 — Source Data File [file 41467_2023_43194_MOESM9_ESM.zip › Source Data File/Raw Data of Western blot/Supplementary Figure 5/replicate 2/eIF2alpha/loading control-alpha Tubulin/blank_label_noPA_nolight.tif]

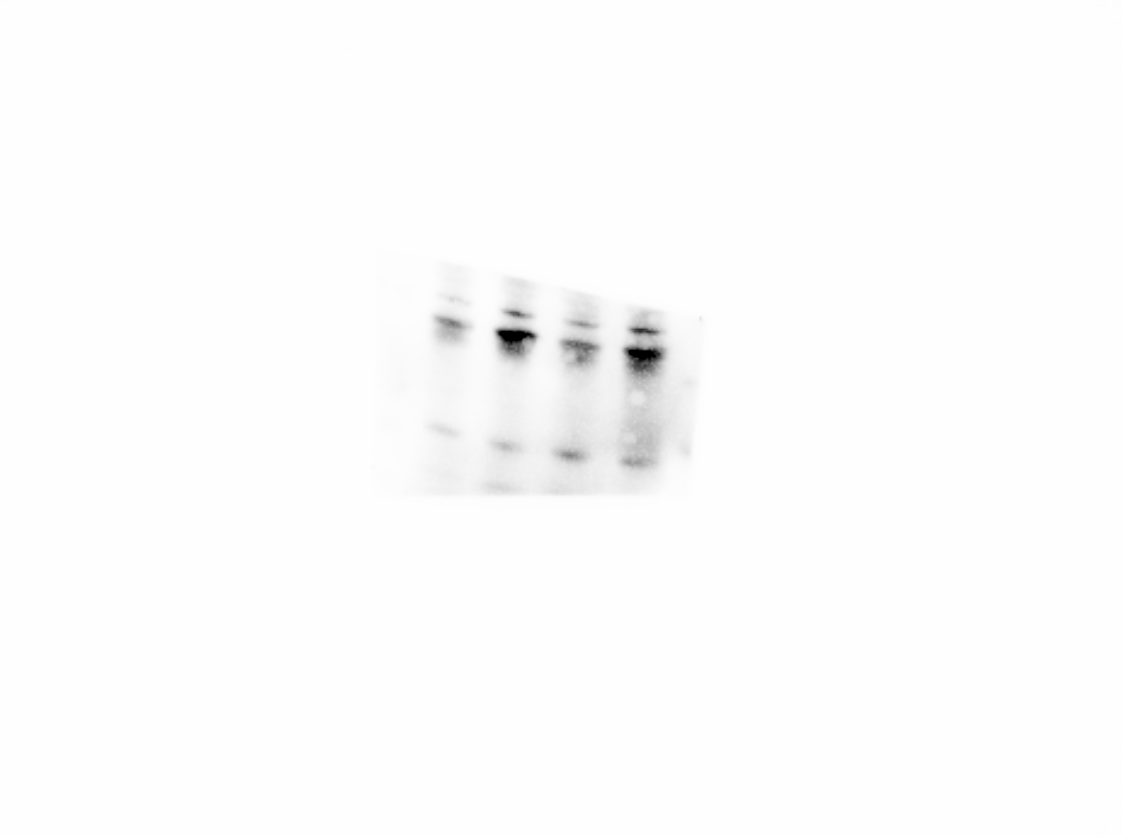

Supplement: Supplementary file 9 — Source Data File [file 41467_2023_43194_MOESM9_ESM.zip › Source Data File/Raw Data of Western blot/Supplementary Figure 5/replicate 3/eIF2alpha phorspho/eIF2alpha phorspho/blank_label_noPA_nolight.tif]

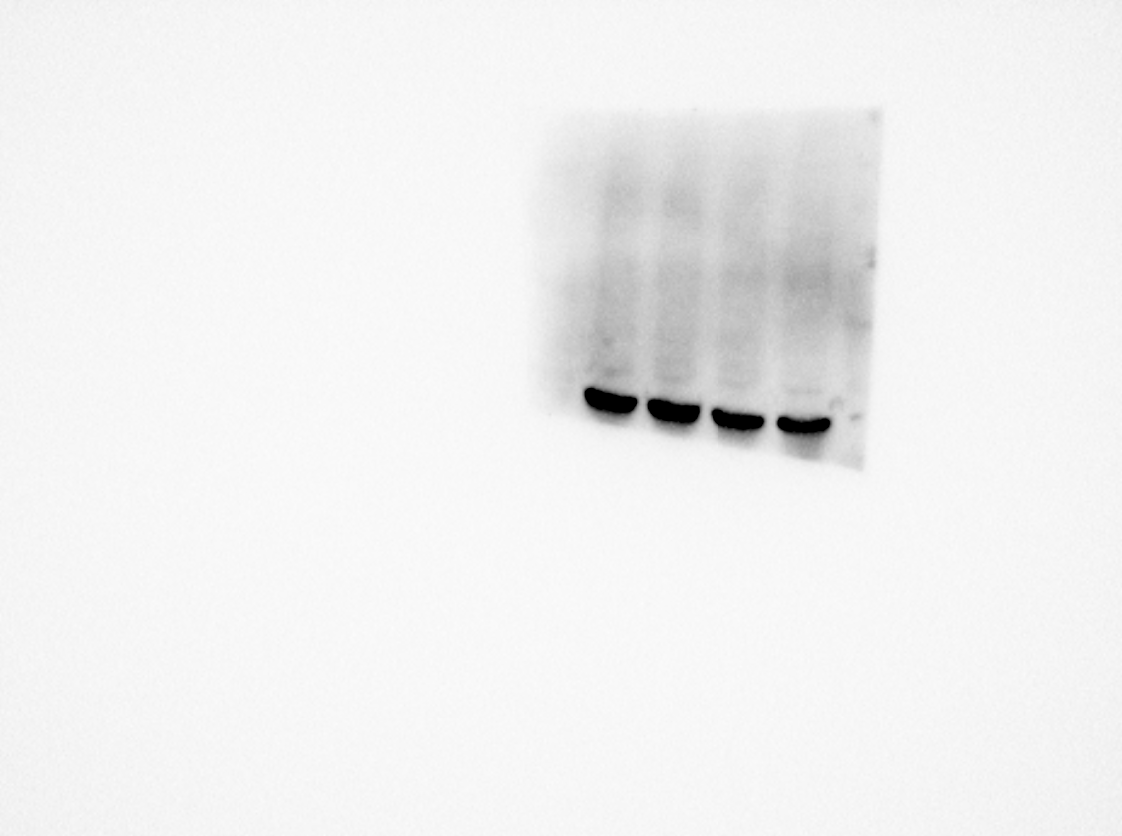

Supplement: Supplementary file 9 — Source Data File [file 41467_2023_43194_MOESM9_ESM.zip › Source Data File/Raw Data of Western blot/Supplementary Figure 5/replicate 3/eIF2alpha phorspho/loading control-alpha Tubulin/blank_label_noPA_nolight Tubulin.tif]

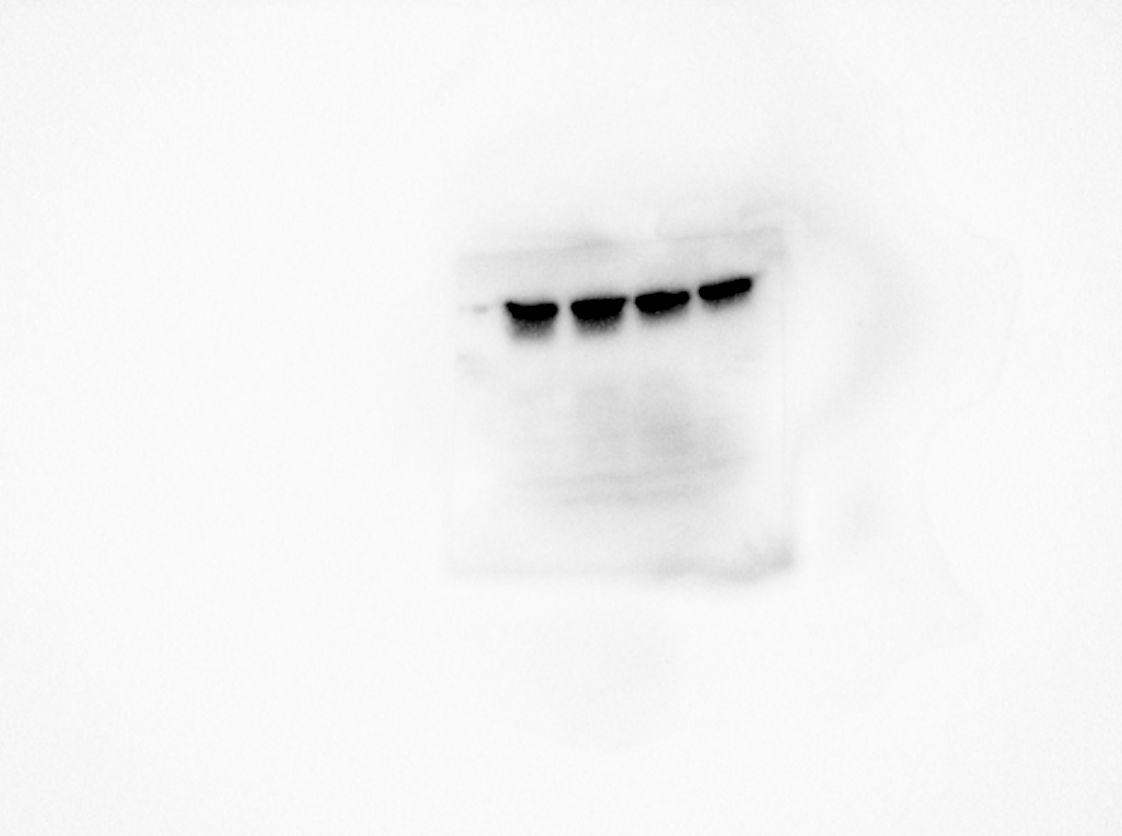

Supplement: Supplementary file 9 — Source Data File [file 41467_2023_43194_MOESM9_ESM.zip › Source Data File/Raw Data of Western blot/Supplementary Figure 5/replicate 3/eIF2alpha/eIF2alpha/blank_label_noPA_nolight-version2.tif]

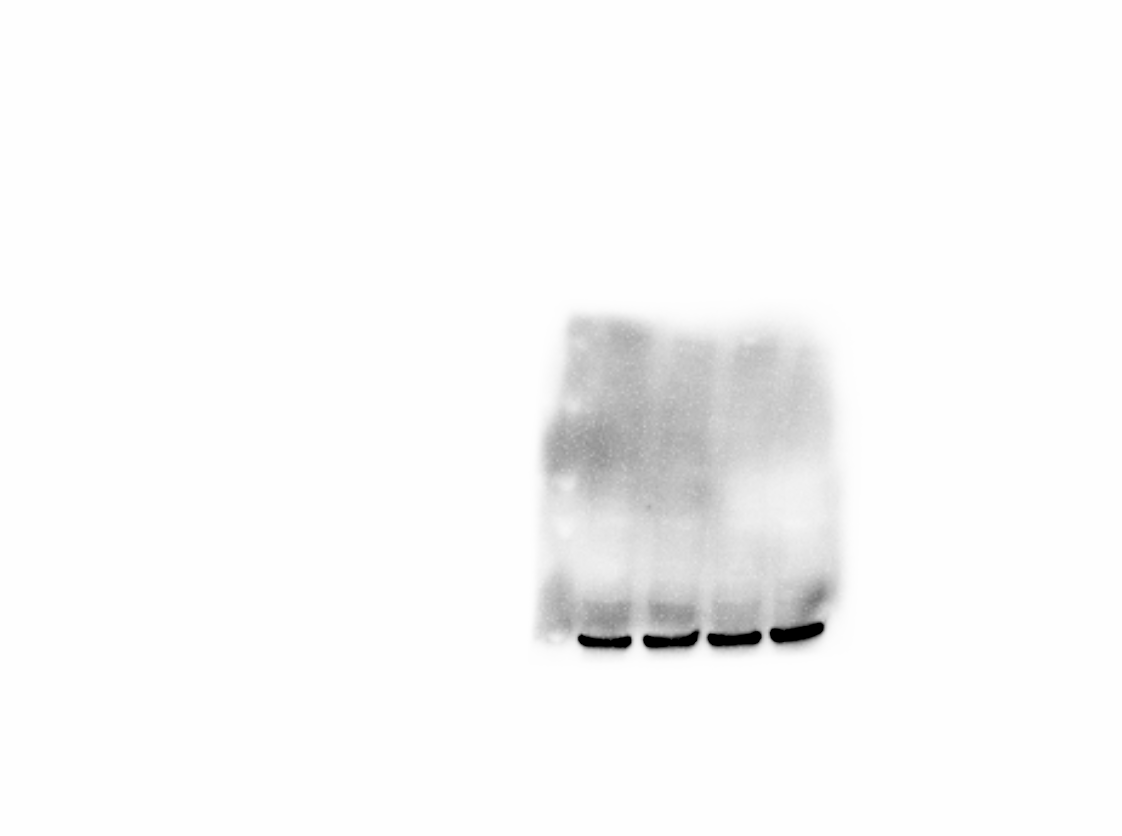

Supplement: Supplementary file 9 — Source Data File [file 41467_2023_43194_MOESM9_ESM.zip › Source Data File/Raw Data of Western blot/Supplementary Figure 5/replicate 3/eIF2alpha/loading control-alpha Tubulin/blank_label_noPA_nolight Tubulin.tif]

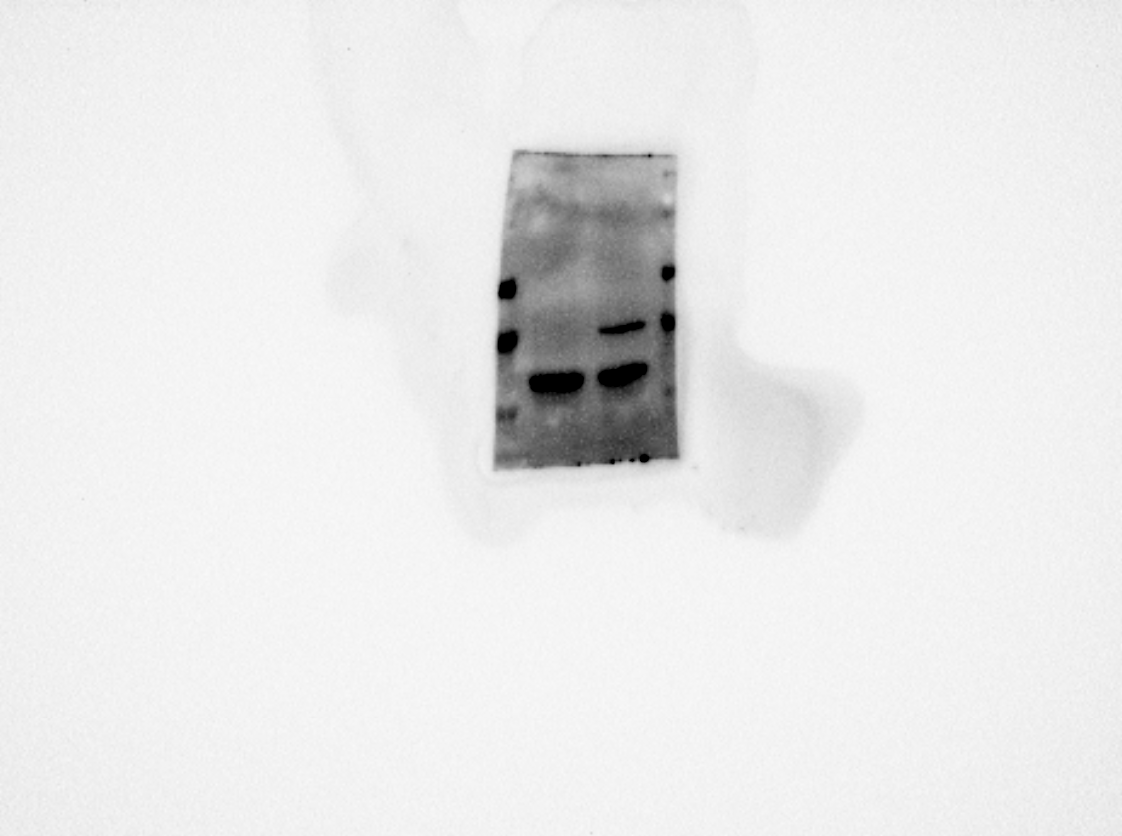

Supplement: Supplementary file 9 — Source Data File [file 41467_2023_43194_MOESM9_ESM.zip › Source Data File/Raw Data of Western blot/Supplementary Figure 8/replicate 1-display in Supplementary Figure 8/G3BP1/U2OS WT_G3BP1-mS U2OS.tif]

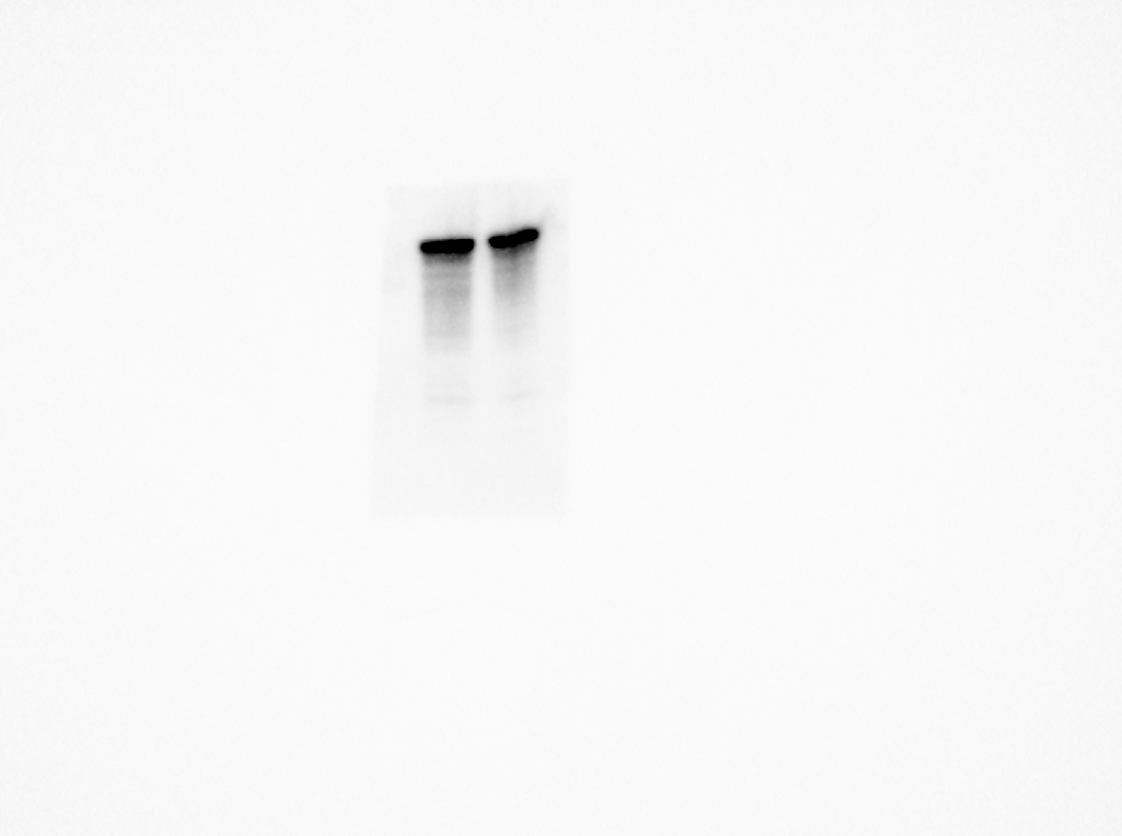

Supplement: Supplementary file 9 — Source Data File [file 41467_2023_43194_MOESM9_ESM.zip › Source Data File/Raw Data of Western blot/Supplementary Figure 8/replicate 1-display in Supplementary Figure 8/loading control-eIF2alpha/U2OS WT_G3BP1-mS U2OS.tif]

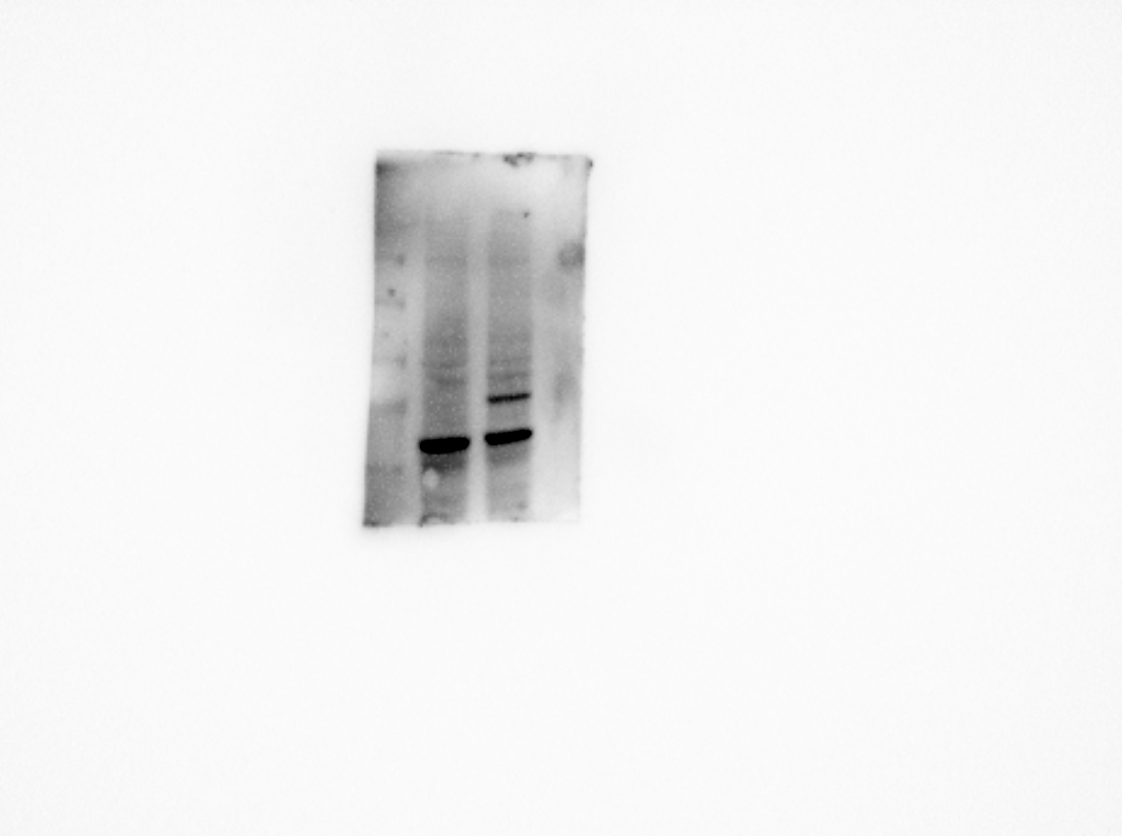

Supplement: Supplementary file 9 — Source Data File [file 41467_2023_43194_MOESM9_ESM.zip › Source Data File/Raw Data of Western blot/Supplementary Figure 8/replicate 2/G3BP1/U2OS WT_G3BP1-mS U2OS.tif]

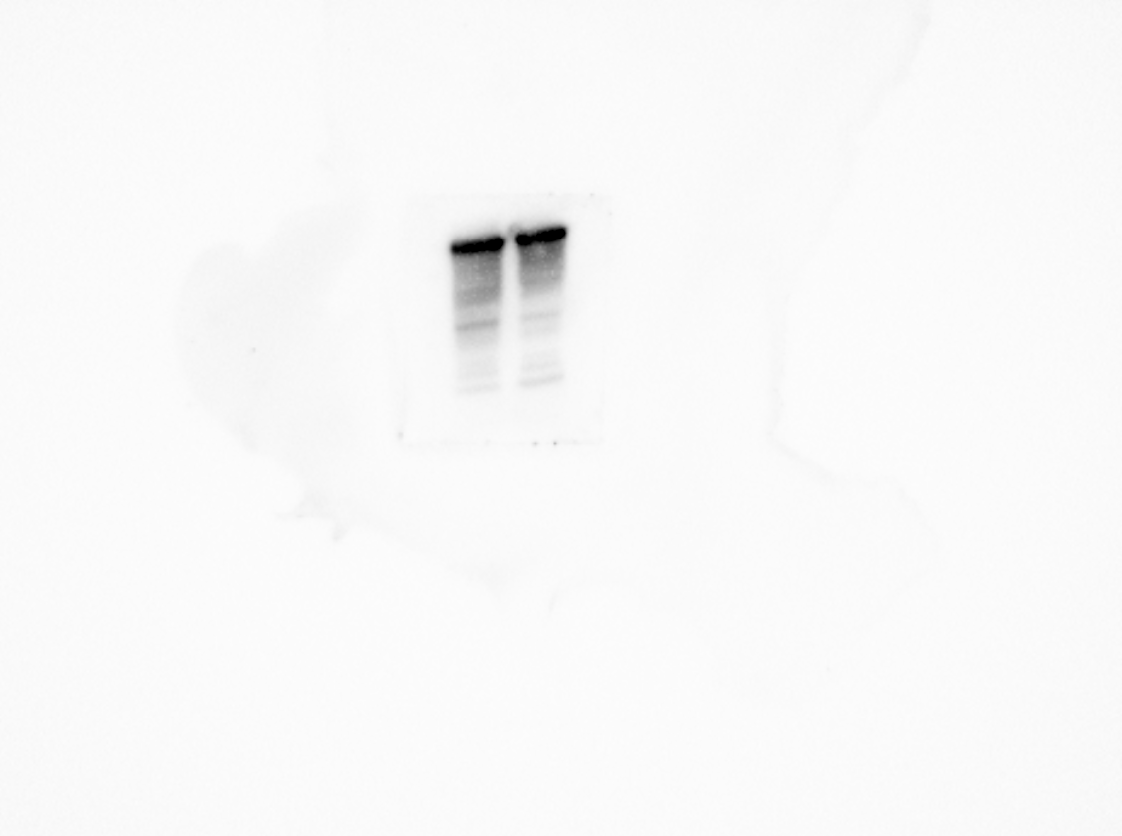

Supplement: Supplementary file 9 — Source Data File [file 41467_2023_43194_MOESM9_ESM.zip › Source Data File/Raw Data of Western blot/Supplementary Figure 8/replicate 2/loading control-eIF2alpha/U2OS WT_G3BP1-mS U2OS.tif]

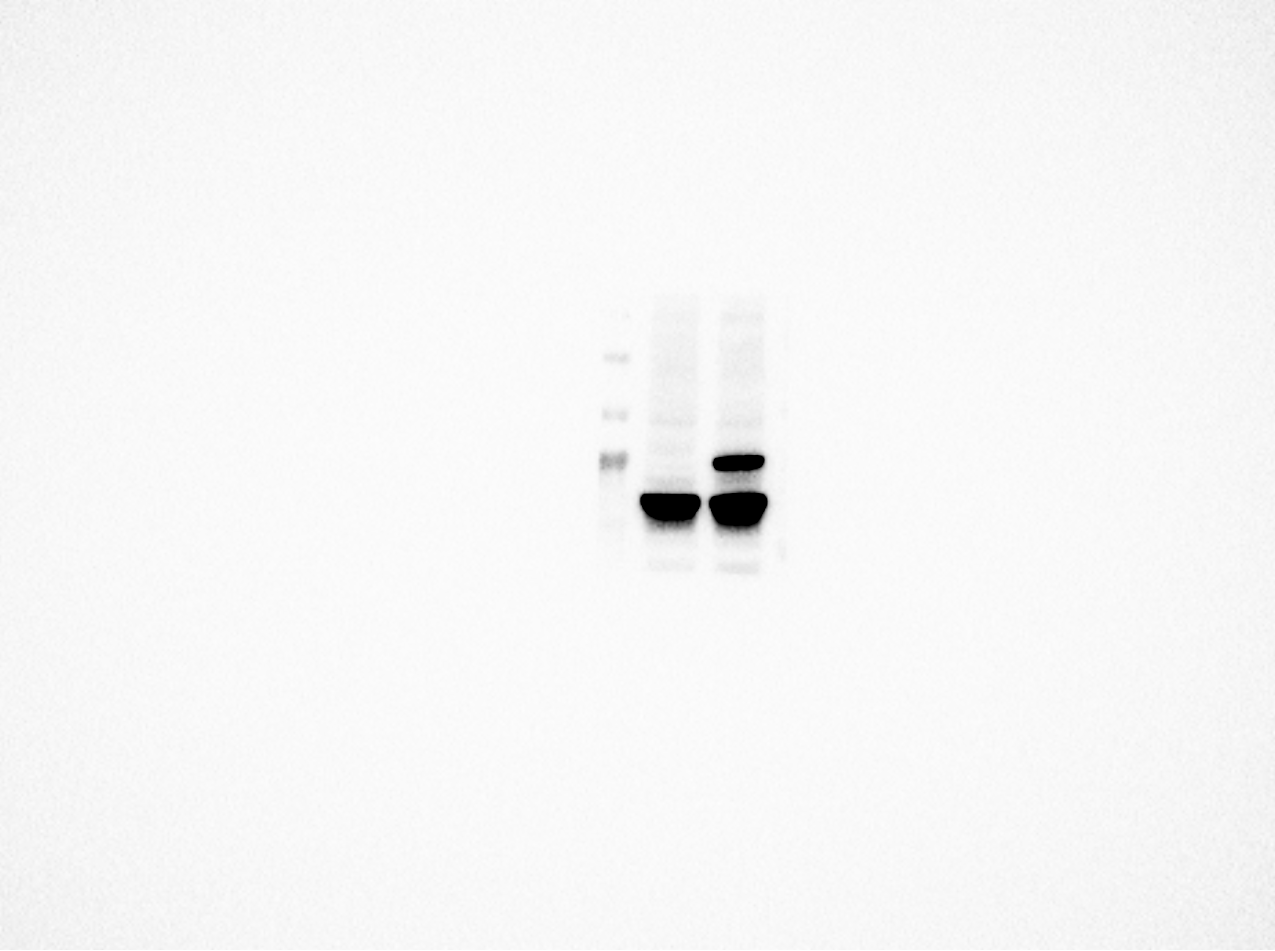

Supplement: Supplementary file 9 — Source Data File [file 41467_2023_43194_MOESM9_ESM.zip › Source Data File/Raw Data of Western blot/Supplementary Figure 8/replicate 3/G3BP1/U2OS WT_G3BP1-mS U2OS.tif]

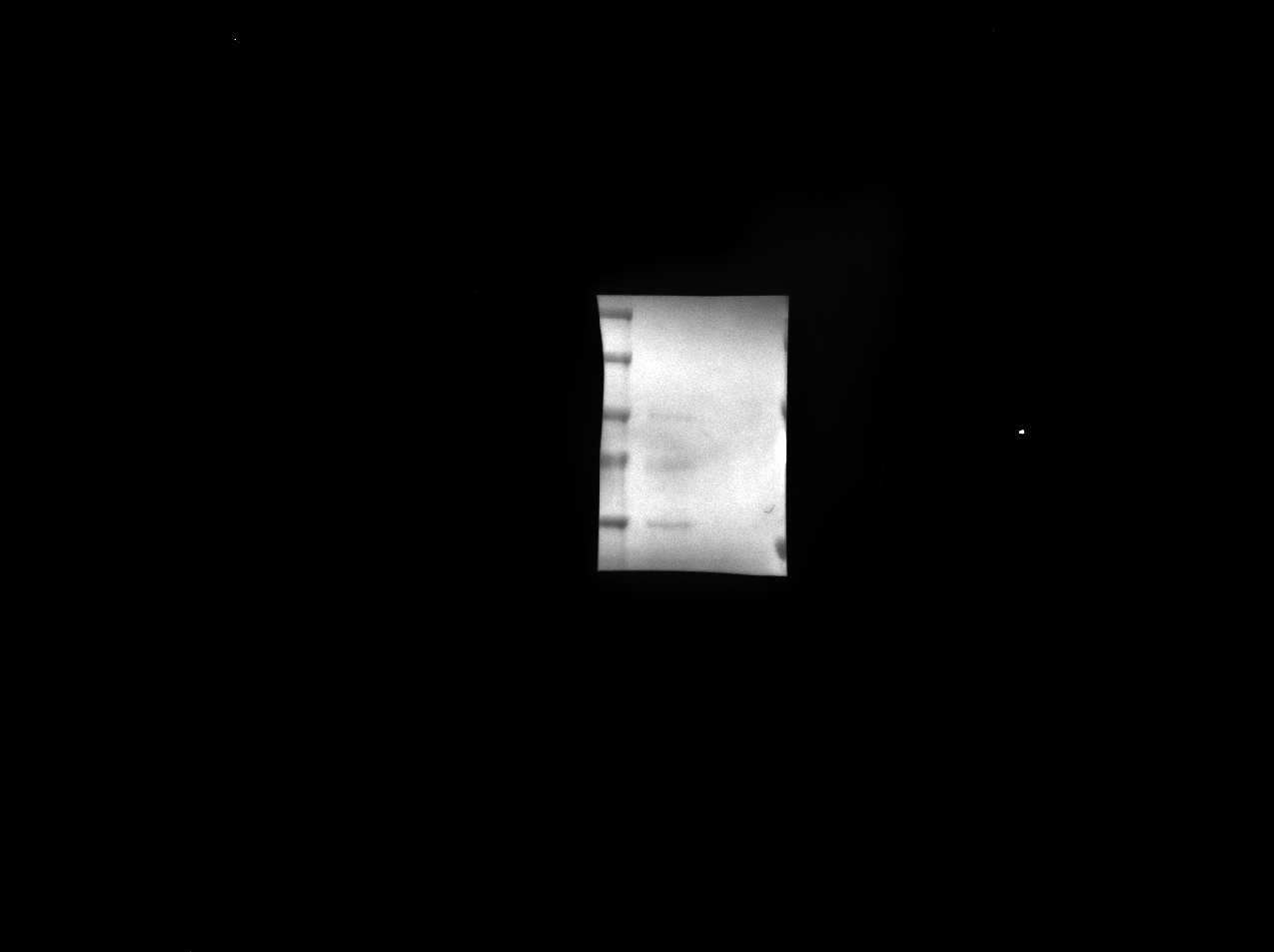

Supplement: Supplementary file 9 — Source Data File [file 41467_2023_43194_MOESM9_ESM.zip › Source Data File/Raw Data of Western blot/Supplementary Figure 8/replicate 3/G3BP1/marker.tif]

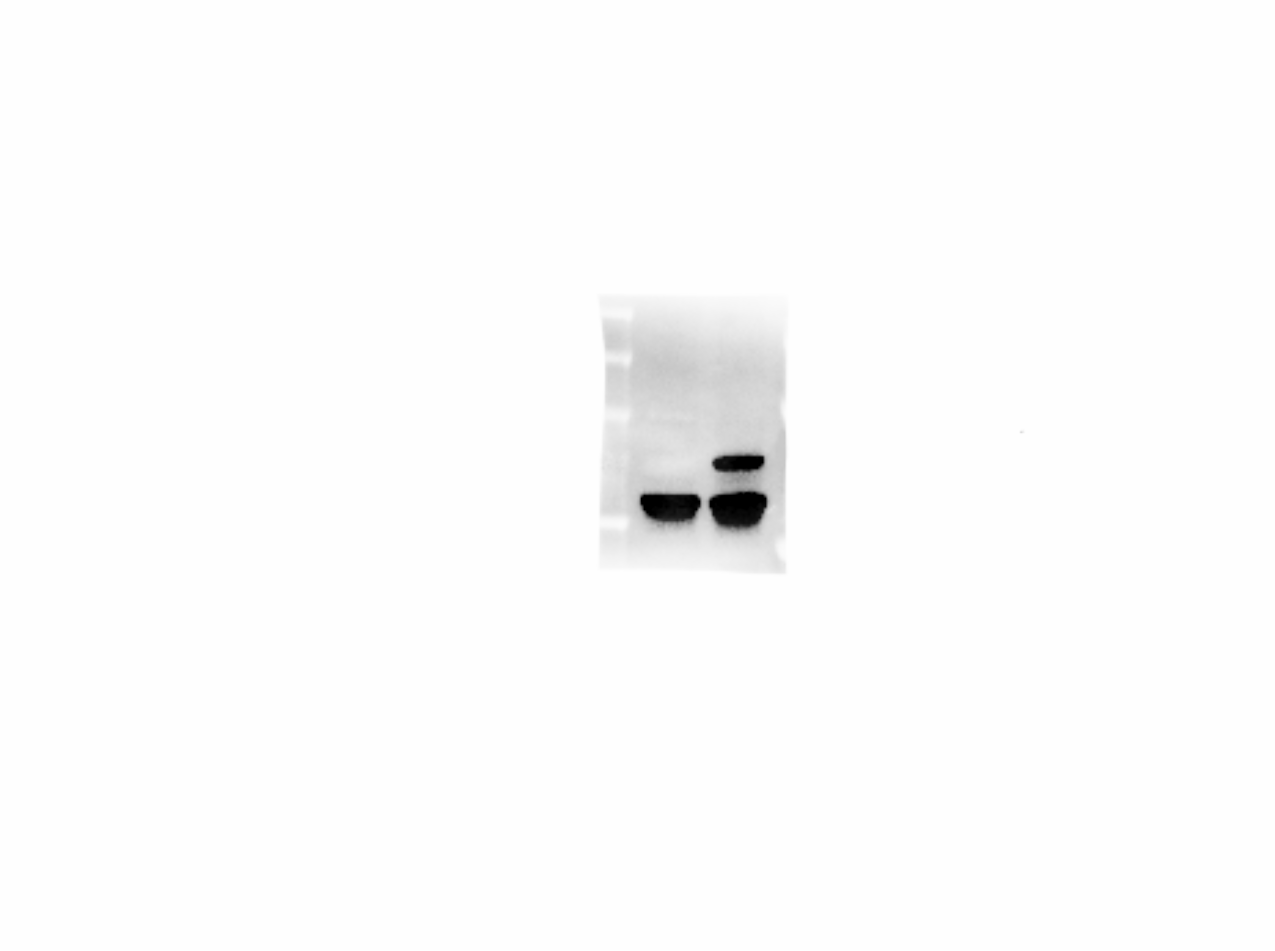

Supplement: Supplementary file 9 — Source Data File [file 41467_2023_43194_MOESM9_ESM.zip › Source Data File/Raw Data of Western blot/Supplementary Figure 8/replicate 3/G3BP1/merge.tif]

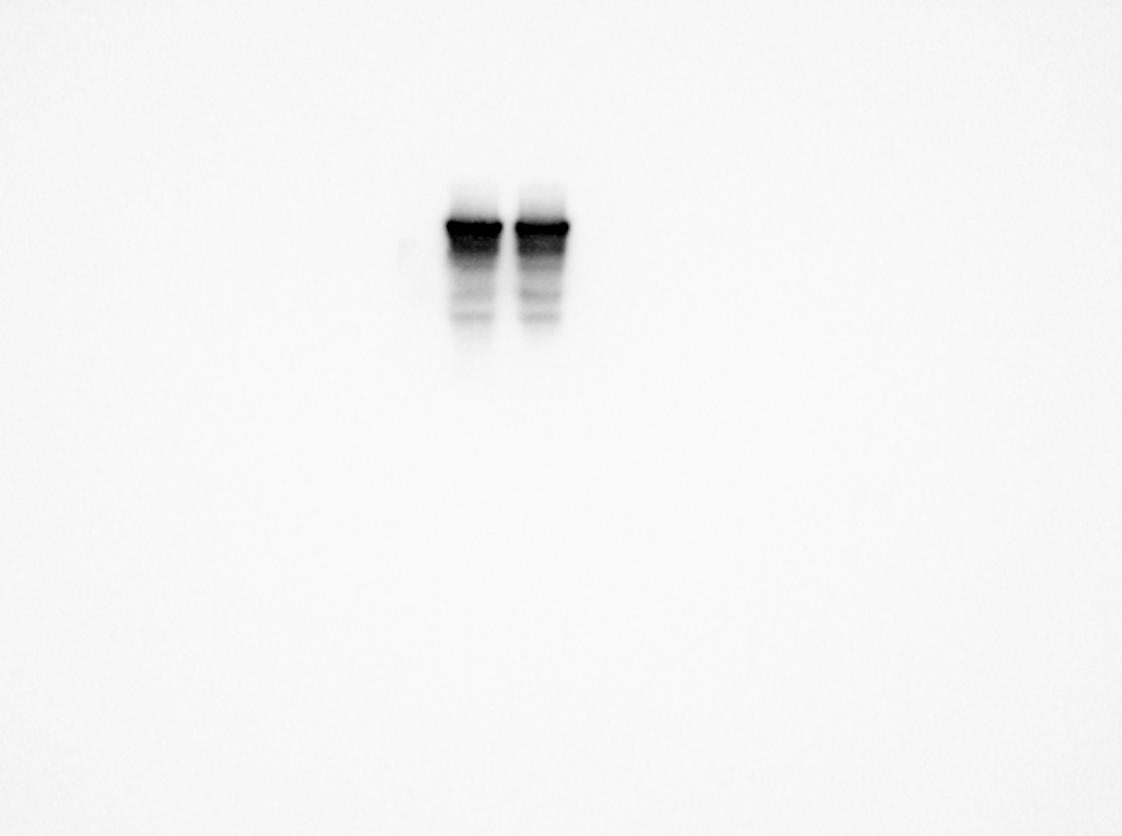

Supplement: Supplementary file 9 — Source Data File [file 41467_2023_43194_MOESM9_ESM.zip › Source Data File/Raw Data of Western blot/Supplementary Figure 8/replicate 3/loading control-eIF2alpha/U2OS WT_G3BP1-mS U2OS.tif]
